# Dynamic growth of slip surfaces in catastrophic landslides

**DOI:** 10.1098/rspa.2015.0758

**Published:** 2016-01

**Authors:** Leonid N. Germanovich, Sihyun Kim, Alexander M. Puzrin

**Affiliations:** 1 Georgia Tech, Atlanta, GA 30332-0355, USA; 2 Bradley University, Peoria, IL 61625, USA; 3 ETH-Zurich, Zurich 8093, Switzerland

**Keywords:** landslide, shear band, dynamic rupture, slip surface, submarine and subaerial slides, Gaviota and Humboldt slides

## Abstract

This work considers a landslide caused by the shear band that emerges along the potential slip (rupture) surface. The material above the band slides downwards, causing the band to grow along the slope. This growth may first be stable (progressive), but eventually becomes dynamic (catastrophic). The landslide body acquires a finite velocity before it separates from the substrata. The corresponding initial-boundary value problem for a dynamic shear band is formulated within the framework of Palmer & Rice's (*Proc. R. Soc. Lond. A* **332**, 527–548. (doi:10.1098/rspa.1973.0040)) approach, which is generalized to the dynamic case. We obtain the exact, closed-form solution for the band velocity and slip rate. This solution assesses when the slope fails owing to a limiting condition near the propagating tip of the shear band. Our results are applicable to both submarine and subaerial landslides of this type. It appears that neglecting dynamic (inertia) effects can lead to a significant underestimation of the slide size, and that the volumes of catastrophic slides can exceed the volumes of progressive slides by nearly a factor of 2. As examples, we consider the Gaviota and Humboldt slides offshore of California, and discuss landslides in normally consolidated sediments and sensitive clays. In particular, it is conceivable that Humboldt slide is unfinished and may still displace a large volume of sediments, which could generate a considerable tsunami. We show that in the case of submarine slides, the effect of water resistance on the shear band dynamics may frequently be limited during the slope failure stage. For a varying slope angle, we formulate a condition of slide cessation.

## 1. Introduction

**Figure 1.**
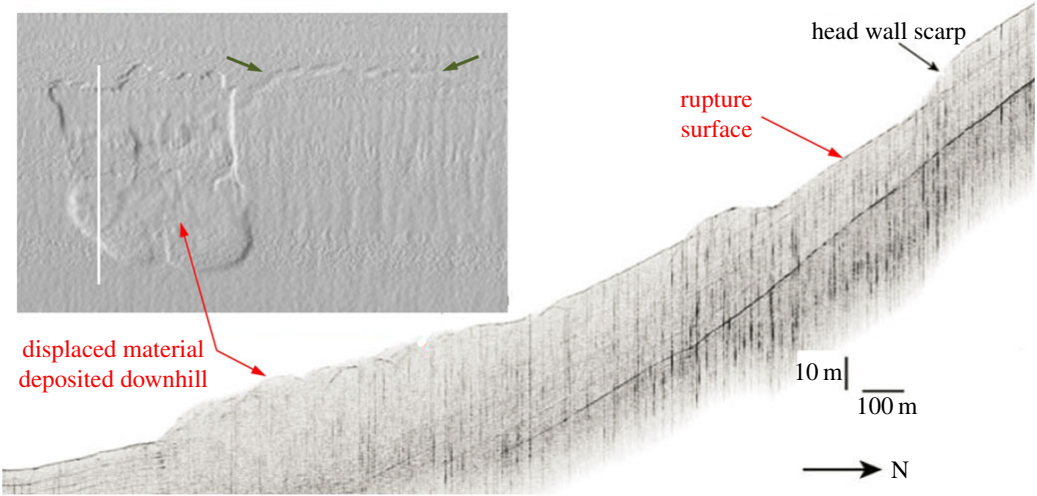
Bathymetry (inset) and seismic profile (along the white line in the inset) images of the Gaviota slide (34°22′ N, 120°06′ W) in the Santa Barbara basin (modified from [12,13]). The slope (‘headscarp’) crack (indicated by arrows, spaced by ≈2.5 km, in the inset) is interpreted as the site of slide initiation owing to the shear surface that starts at the crack and develops downhill subparallel to the slope surface [13].

The term ‘landslide’ refers to a broad range of processes that result in gravity-assisted, downslope movement of slope-forming materials. Landslide occurrence, morphology, distribution, scale and consequences have been discussed in detail [1–8]. This work considers slides caused by a shear band that develops below the sliding mass along the potential slip (rupture) surface in long slopes [1,5,9,10]. Within the band, the shear strength drops owing to the softening behaviour of the particulate material (e.g. [5] and references herein). The sediment above this weakened zone moves downwards, causing the shear band to propagate and create the rupture surface. When the shear band reaches a sufficiently large size, the propagation becomes dynamic (fast) [1,11], which produces a finite slide velocity already before the slide separates from the substrata and moves downslope (figure 1).

When the shear band begins growing dynamically, the induced wave propagates in the overlaying layer away from the band tip and unloads the layer near the tip. Hence, the failure in the dynamic case is expected to take place for a larger band length than in the static case. Therefore, the static approach can result in an underestimation of the displaced volume. Underestimating the displaced volume and initial velocity of the slide body could, in turn, result in underestimating the slide damage and tsunami potentials. Thus, it is important to evaluate the dimensions and velocities of a landslide body and the corresponding shear band.

The analysis in this work is based on the energy balance approach of Palmer & Rice [1], which is the basis for understanding the progressive (quasi-stable) shear band growth in natural slopes [9,10,13–22]. For a shear band to propagate, the energy surplus produced in the body during an incremental propagation must exceed the energy required for this propagation. The analysis of the band propagation in a submerged slope, consisting of normally consolidated clays, has shown that a relatively short initial weakness zone [23,24] or progressively propagating shear band [19] is sufficient to cause a full-scale landslide [10,15].

Within the framework of Palmer & Rice's [1] approach, we formulate an initial-boundary value problem for a dynamic shear band propagating along the slope. We obtain the exact, closed-form solution for the band and landslide velocities as well as for the spatial and temporal distributions of strain and slip rate. This solution assesses when the slope fails owing to a limiting condition (e.g. passive failure) near the propagating tip of the shear band. Our results are applicable to both submarine and subaerial landslides of this type. The dynamic version of Palmer & Rice's [1] model of slip surfaces in overconsolidated sediments is a particular case of our formulation.

In this work, the terms ‘rupture surface’ and ‘slip surface’ are synonymous to the basal detachment boundary created by shear band growth. In addition, the terms ‘shear band’ and ‘rupture’ are synonymous to ‘mode II crack’ or ‘in-plane shear fracture’. The term ‘progressive’ is used as the opposite to ‘instantaneous’ or ‘catastrophic’ and without regard to the direction of band growth (upslope or downslope). Progressive growth is quasi-stable [5,19] in contrast with the catastrophic growth when dynamic (inertia) effects are important.

Conceptually, a slide develops from the quasi-equilibrium state of the slope material and involves ‘failure’ and ‘post-failure’ stages [3,25]. During the failure stage, a continuous rupture surface develops in the slope. This stage ends with ‘(global) slope failure’, when the sliding body is separated from the underlying sediment. The separated body moves outward and downhill during the post-failure stage (figure 1). This work considers the dynamic phase of shear band growth during the failure stage. As an example, we analyse the Gaviota (figure 1) and Humboldt slides, and discuss landslides in normally consolidated sediments and sensitive clays.

## 2. Shear band in an infinite slope

### (a) One-dimensional model

**Figure 2.**
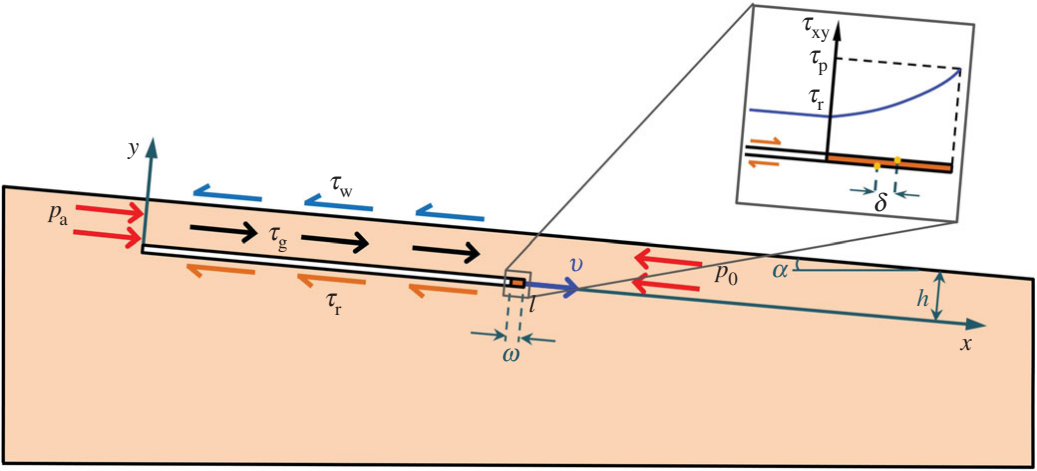
Shear band in an infinite slope. The slab above the slip surface is already deformed during the stable (progressive) stage of the band growth, whereas the sliding velocity appears in the dynamic stage that begins at *t*=0.

Landslides may develop by the shear band propagating in upslope or downslope directions (or both) [16,26]. Consider a shear band of length *l* at depth *h*, parallel to the surface of the infinite slope [19], which is inclined at angle *α* to the horizontal (figure 2). The band propagates down the slope and parallel to the slope surface. Upslope propagation is discussed in §8a. The nomenclature of symbols is given in the electronic supplementary material, appendix A.

Various mechanisms such as earthquake-triggered liquefaction [27], methane-hydrate decomposition [8,28,29], excess pore pressure (i.e. pressure above hydrostatic) induced by rapid sedimentation [3,11] and local fluid fluxes [11,30] may cause the initial weak zone [23,24]. The landslide evolution, therefore, may be rather complex. In this work, we simply assume that the dynamic (catastrophic) growth of the shear band is preceded by the quasi-stable (progressive) growth until the band size reaches some *critical length*, *l*_0_. After that, the shear band propagates dynamically along the slope (figure 2), which eventually leads to the slope failure [10,14–16,18,19]. The initial weak zone does not need to be of the observed landslide length, *l*_f_, which may be as large as of the order of 10^2^ km [31]. It is sufficient, instead, that the initial zone reaches the critical length, *l*_0_, which is smaller (possibly, much smaller) than *l*_f_. In the dynamic analysis, *l*_0_ is the initial length of the shear band, which is defined based on the static analysis using the energy balance condition.

We also assume that *l*≫*h*≫*ω*, where *ω* is the size of the process zone (figure 2) at the band tip, *x*=*l*(*t*), where *t* is time. Within this small zone, the shear resistance, *τ*_*xy*_, of the material gradually decreases from the peak, *τ*_p_, to the residual, *τ*_r_, value as a function of the relative slip displacement, *δ* [1]. Everywhere else in the band, the shear resistance is *τ*_r_. At the tip, *x*=*l*+*ω*, of the process zone, the shear resistance is equal to the peak value, *τ*_p_. The downhill component, *τ*_g_, of the gravitational stress is the driving force that causes the material above the band to move downwards. This makes the band propagate along the slope (figure 2), until the slope fails (§5). If the band grows in a submerged slope, then the slope surface above the band slides downward and mobilizes the water resistance, *τ*_w_, on the seafloor (figure 2).

Conditions *l*≫*ω* and *h*≫*ω* represent the asymptote of the small-scale yielding. The slab above the shear band is considered thin when *l*≫*h*. These conditions are relaxed by Viesca & Rice [11], who studied slip surface nucleation and growth driven by locally elevated pore pressure. Condition *h*≫*ω* is also relaxed by Puzrin & Germanovich [10] for a quasi-static shear band propagating both upslope and downslope. In this work, we keep these conditions to simplify the treatment of the dynamic shear band.

Consider the thickness-averaged values [1],

2.1 $$\sigma =\frac{1}{h}\int_{0}^{h}\sigma_{xx}\;\text{d}y,\;u=\frac{1}{h}\int_{0}^{h}u_{x}\;\text{d}y\;\text{and}\;p=\frac{1}{h}\int_{0}^{h}P\;\text{d}y,$$

of the total longitudinal stress, *σ*_*xx*_, displacement, *u*_*x*_ and pore pressure, *P*. Here, (*x*,*y*) is the coordinate set with *x* being the coordinate along the slope (figure 2). We assume that during the relatively short time of the shear band growth, the deformation is undrained at the scale of the thickness of the sliding layer. In general, diffusion at the scales of the band process zone and band thicknesses may or may not be neglected, which affects the sediment strength at these scales. As *u*_*x*_ on the lower band side is negligible [1,32–34] (at least sufficiently far from the tip zone), the thickness-averaged displacement, *u*, in (2.1) represents the relative slip between the band sides.

Before the shear band appears, the thickness-averaged effective stress in the intact slope is *σ*_0_+*p*=−*p*_0_ (*p*_0_>0). Because *p*_0_ typically does not significantly change along a long, mild slope, the slope can be approximated by an infinite slope with constant *p*_0_ [1]. Hereafter, compressive stresses are negative, and displacements and strains are measured with respect to the undeformed state in the infinite slope with homogeneous, longitudinal effective stress, *p*_0_ (figure 2). Pore pressure *P*(*x*,*y*) in the infinite slope is considered hydrostatic (although it may also include excess pressure Δ*P*(*y*)).

Both submarine and subaerial landslides tend to show a combination of brittle-like fractures and plastic deformation (such as creep). These features, however, develop at distinctly different time scales. In this paper, we are concerned with a relatively small, incrementally elastic, undrained deformation of the sliding body at a relatively short timescale of shear band growth. Accordingly, the sediment behaviour in the sliding layer is described, in plane strain, by

2.2 $$\sigma +p=E\frac{\partial u}{\partial x}-p_{0},$$

where *E*=2*G*/(1−*ν*_u_),*G* is the shear modulus, *ν*_u_ is the undrained Poisson ratio and *p* is the thickness-averaged pore pressure prior to the shear band appearance.

### (b) Dynamic motion

Governing equations can now be formulated in terms of the thickness-averaged quantities (2.1). The momentum balance condition results in a wave equation

2.3 $$\frac{\partial^{2}u}{\partial x^{2}}-\frac{1}{c^{2}}\frac{\partial^{2}u}{\partial t^{2}}=-\frac{T}{h},$$

where *c*=(*E*/*ρ*_0_)^1/2^ is the speed of the longitudinal, plain–strain elastic waves in the one-dimensional layer sliding above the shear band (figure 2), *ρ*_0_ is the bulk material density (which accounts for both solid matrix and pore fluid), *T*=τ_*_/*E*>0 is the normalized, distributed, longitudinal load and

2.4 $$\tau_{*}=\tau_{g}-\tau_{b}-\tau_{r}-\tau_{w}$$

is the combined gravitational, *τ*_g_=*ρ*_0_*gh*sin*α*, buoyant, *τ*_b_=*ρ*_w_*gh*sin*α*, frictional, *τ*_r_=*μ*(*ρ*_0_−*ρ*_w_)*gh*cos*α* and viscous (for submerged slopes), *τ*_w_, loads in the slope direction (figure 2). The water density, *ρ*_w_, is the same in the porous space and in the water column.

The initial conditions are

2.5 $$u(x,t)=u_{s}(x)\;\mathrm{and}\;\frac{\partial u}{\partial t}(x,t)=0\;(t\to +0),$$

where the thickness-averaged displacement *u*_*s*_(*x*) is accumulated before the band begins propagating dynamically. The slide (slope) is initially at rest (quasi-stable equilibrium), but has already moved during the progressive (yet relatively fast to be undrained) stage of the deformation process.

Condition *u*(*l*(*t*),*t*)=0 (*t*>0) at the tip, *x*=*l*(*t*), of the propagating shear band can be written as [32,33]

2.6 $$\frac{\partial u}{\partial t}=-v\frac{\partial u}{\partial x}\;(x=l),$$

where *v*=d*l*/d*t* is the velocity of the band tip. Because the model developed here is based on the small strain assumption, |∂*u*/∂*x*|≪1, (2.6) implies that ∂*u*(*l*,*t*)/∂*t*<*v*. Hence, the material at the band tip moves slower than the tip, which is a necessary condition for our model to be physically acceptable. It will be shown formally in §3 that this condition is satisfied.

At the top end, *x*=0, of the sliding slab (figure 2), the sediment undergoes *active* failure [6,19] characterized by the active stress, *p*_*a*_. Per (2.2), the corresponding effective stress *σ*+*p*=−*p*_*a*_ (*p*_*a*_=const.) at *x*=0 can be expressed as a boundary condition in terms of strains

2.7 $$\frac{\partial u}{\partial x}(0,t)=\gamma_{a},$$

where *γ*_*a*_=(*p*_0_−*p*_*a*_)/*E*. Typically, *p*_*a*_<*p*_0_ because of the sediment unloading compared with the infinite slope.

Alternatively, in a sediment with sufficient cohesion, a tensile crack may develop at *x*=0 connecting the shear band with the slope surface. As the water fills the crack (in the case of a submerged slope), the slide body becomes loaded by the hydrostatic pressure, *p*_*h*_, which is the same in the crack and in the sediment near the crack walls. We denote the corresponding zero effective stress at *x*=0 also by *p*_*a*_ (*p*_*a*_=0).

Hence, in either case, *p*_*a*_<*p*_0_ and, according to (2.2), *γ*_*a*_>0. In some highly overconsolidated sediments (and rocks), *γ*_*a*_ may become negative. Yet, it is more likely that *γ*_*a*_≥0; particularly, for submarine slopes that are typically composed of normally consolidated or lightly overconsolidated sediments. Therefore, we further consider *γ*_*a*_≥0. The developed model, however, is also applicable to the case of negative *γ*_*a*_ if |*γ*_*a*_|<*γ*_0_, where *γ*_0_ is the initial tip strain magnitude (defined in §2c), at the onset of dynamic growth.

### (c) Energy balance

In conditions of small-scale yielding, the shear band propagation is controlled by the energy balance at the band tip. For a dynamically propagating band, the energy release rate, *J*, is balanced by the rate, *J*_*c*_, of the energy dissipation at the band tip. Quantity *J*_*c*_ can be interpreted as the ‘apparent’ surface energy for a growing band. It is assumed constant, although in the landslide context, this is not necessarily the case even for small-scale yielding as different weakening mechanisms may take place during the band growth. As noted by Viesca & Rice [11], for example, rapid slip may result in shear heating, which, in turn, may cause thermal pressurization or material decomposition to occur in a sediment [35–37]. The specific nature of the energy dissipation is not important for the model developed in this work, however, and condition *J*=*J*_*c*_ at the propagating band tip, *x*=*l*(*t*), rewrites as [32,33]

2.8 $${\left[\frac{\partial u}{\partial x}(l,t)\right]}^{2}=\frac{\gamma_{c}^{2}}{1-v^{2}/c^{2}}\;(l(t)>l_{0},t>0),$$

where *γ*_*c*_=[2*J*_*c*_/(*hE*)]^1/2^ is the minimal strain level at the tip of the band (when *v*→0), and for a meaningful solution, *v*<*c*. The local energy balance condition (2.8) can also be obtained by employing the global energy balance criterion for the moving (and ‘growing’) one-dimensional layer.

When *t*=0, the initial shear band has a length, *l*_0_, such that at the given level, *T*, of the applied load, it is just about to start propagating (dynamically). This length is defined by (2.8) with *v*=0 and *γ*_*c*_ replaced by *γ*_0_=[2*J*_0_/(*hE*)]^1/2^, which is the strain level required at the tip for the static band to begin propagating. Here, $J_{0}=(\tau_{p}-\tau_{r})\bar{\delta }$ is the surface energy for the static shear band with $\bar{\delta }$ being the characteristic slip at the band tip [1].

Hence, at *t*=0, (2.8) can be replaced by

2.9 $$\gamma_{s}(l_{0})=-\gamma_{0},$$

where *γ*_*s*_(*l*_0_)<0 is the thickness-averaged, static, longitudinal strain at the end, *x*=*l*_0_, of the initial layer above the shear band (figure 2). This strain is defined by

2.10 $$\gamma_{s}(x)=\frac{\text{d}u_{s}}{\text{d}x}=\gamma_{a}-\frac{1}{h}\int_{0}^{x}T(x,0)\;dx,$$

which is obtained by integrating (2.3) with ∂^2^*u*/∂*t*^2^=0 and boundary condition (2.7). The layer is in a state of dynamic motion as the shear band grows, but introducing the auxiliary function (2.10) is handy to characterize the virtual steady state (if the layer were not moving).

Because *J*_0_≠*J*_*c*_, and, hence, *γ*_0_≠*γ*_*c*_, comparing (2.9) and (2.8) shows that at *t*=+0, the band tip instantaneously acquires some finite propagation velocity, *v*_0_. Quantity

2.11 $$n=\frac{\gamma_{0}^{2}}{\gamma_{c}^{2}}=\frac{J_{0}}{J_{c}},$$

is called the ‘bluntness’ parameter [32] as applied to open (mode I) cracks. For open fractures, *J*_0_ can be greater than *J*_*c*_ [38–40], and *n*>1 is also used for shear fractures [32,33]. Transition from *J*_0_ to *J*_*c*_ could be addressed, in principle, by employing more accurate friction laws that depend upon the relative slip and/or slip rate [41,42–44]. Then, the material motion and strain at the band tip would initiate from the state of rest [11,45]. Within the framework of our simplified model, this transition time from rest to dynamic motion is considered to be relatively short, and is approximated by the abrupt increase in propagation velocity from zero to some *v*_0_ or/and by the instantaneous strain change at the tip from *γ*_*s*_(*l*_0_) to *γ*(*l*_0_,+0), which is to be found.

The developed model is also applicable to an alternative scenario, when *γ*_0_=*γ*_*c*_, but the residual friction changes on the slip surface as a result of slip (§8c). In this case, the shear band accelerates rapidly, but there is no initial velocity jump (i.e. *v*_0_=0).

### (d) Initial-boundary value problem

Equations (2.3)–(2.7) can be rewritten in terms of the longitudinal strain, *γ*(*x*,*t*)=∂*u*/∂*x*, slip rate, *η*(*x*,*t*)=∂*u*/∂*t* and fracture length, *l*(*t*), as

2.12 $$\begin{array}{rl} \frac{\partial \gamma }{\partial t} & =\frac{\partial \eta }{\partial x},\;\frac{\partial \gamma }{\partial x}+\frac{T}{h}=\frac{1}{c^{2}}\frac{\partial \eta }{\partial t}\;(0<x<l(t),t>0), \end{array}$$

2.13 $$\begin{array}{rl} \gamma (x,0) & =\gamma_{s}(x),\;\eta (x,0)=0,\;l(0)=l_{0}\;(0<x<l_{0}) \end{array}$$

2.14 $$\begin{array}{rl} \text{and}\;\gamma (0,t) & =\gamma_{a},\;\eta (l,t)=-v(t)\gamma (l,t)\;(t>0). \end{array}$$

To close the set of equations (2.12)–(2.14), we also use an additional condition at the tip of the propagating shear band that combines (2.8) and (2.9) into

2.15 $$\frac{\gamma^{2}}{\gamma_{0}^{2}}=\left\{ \begin{array}{ll} 1 & \left(x=l_{0},t=0\right)\\ \frac{1}{n\left(1-v^{2}/c^{2}\right)} & (x=l(t),t>0), \end{array}\right.$$

where *n* is given by (2.11). In this one-dimensional model, the ‘band tip’ is understood as the end, *x*=*l*(*t*), of the sliding layer. The initial value *l*_0_ of *l*(*t*) is defined by (2.9) and (2.10). In §4, *l*_0_ is expressed explicitly for a particular type of dependence *T*(*x*,*t*).

Set (2.12) of the first-order differential equations is hyperbolic and equivalent to [46,47]

**Figure 3.**
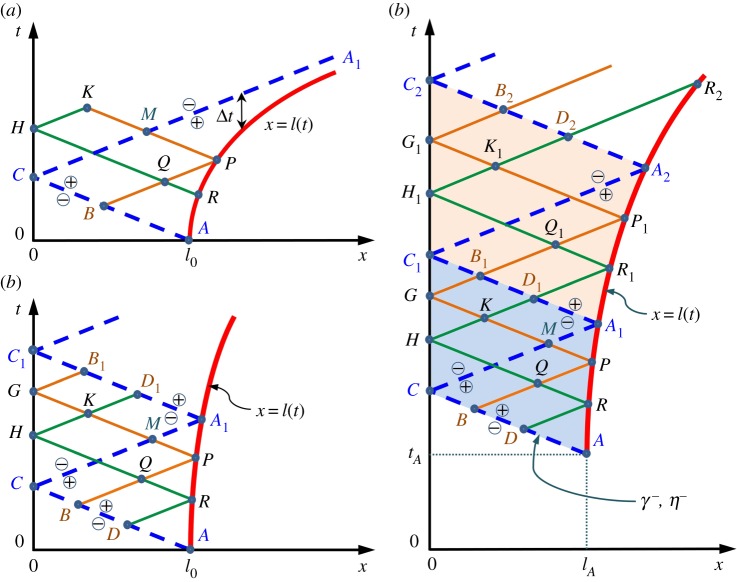
Characteristic lines in the (*x*,*t*) plane. The discontinuity (dashed lines) and waves (thin, solid, straight lines) travel in the layer sliding above the shear band (figure 2), as the band tip, *x*=l(*t*) (bold, solid, curved lines), propagates. (*a*) The discontinuity, which originates at the initial tip position, *x*=l_0_, and then reflects from *x*=0, does not ever reach the band tip again (*A*_1_ is the infinite point in this case). (*b*) The discontinuity reaches the band tip at point *A*_1_, where it reflects and propagates back to *x*=0. (*c*) Two consecutive steps of the recurrence process are represented by regions *ACC*_1_*A*_1_ and *A*_1_*C*_1_*C*_2_*A*_2_. The discontinuity either initiates at point *A* (if l_*A*_=l_0_, *t*_*A*_=0; step shown in (*b*)) or it reflects from point *A* (if l_*A*_>l_0_, *t*_*A*_>0). Function *δ*^−^ (equation (3.19)) on the characteristic line, *AC*, is either specified by the initial conditions (3.2) (if l_*A*_=l_0_, *t*_*A*_=0) or it is found during the previous recurrence step (if l_*A*_>l_0_, *t*_*A*_>0). This function defines all unknowns at the current recurrence step (in *ACC*_1_*A*_1_), including function *δ*^−^ on *A*_1_*C*_1_. The latter function defines the solution at the next step (in *A*_1_*C*_1_*C*_2_*A*_2_).

2.16 $$\frac{\text{d}}{\text{d}x}\left(\gamma \pm \frac{\eta }{c}\right)=-\frac{T}{h}\;\left(\frac{\text{d}t}{\text{d}x}=\mp \frac{1}{c}\right),$$

where d/d*x*=∂/∂*x*+(∂*t*/∂*x*)∂/∂*t* is the total derivative. Quantities γ±η/c+(1/h)∫T dx do not change along the ‘characteristic’ lines d*t*/d*x*=±1/*c* in plane (*x*,*t*) (figure 3). Partial differential equations (2.12), therefore, are reduced to ordinary differential equations (2.16) on characteristics.

According to (2.15), at *t*=0, the tip strain changes from its static, *γ*_*s*_(*l*_0_)=−*γ*_0_, to dynamic, $\gamma (l_{0},+0)=-\gamma_{c}{\left(1-v_{0}^{2}/c^{2}\right)}^{-1/2},$ value. In a hyperbolic system, such an instantaneous change in the boundary condition causes a discontinuity [46–48] that propagates with velocity *c* from the shear band tip, *x*=*l*_0_, towards the other boundary, *x*=0 (figure 2). Henceforward, term ‘discontinuity’ is reserved for this discontinuity in the thickness-averaged quantities, *γ* and *η*, rather than for the shear displacement discontinuity on the shear band itself.

At time *t*=*l*_0_/*c*, the discontinuity reflects from the slide end, *x*=0, and propagates back towards the band tip, *x*=*l*(*t*). In the simplest case, it does not reach the moving tip anymore (figure 3*a*). If it does, it reflects at the tip and propagates again towards *x*=0 (figure 3*b*). Within the framework of elastic theory, this process can continue indefinitely, although it is also possible that the discontinuity will cease reaching the band tip after a number of reflections. Because in the physical space, the discontinuity propagates with velocity *c*, on plane (*x*, *t*), it moves along the ‘discontinuity characteristics’ (figure 3). In the following, we look for functions *γ*(*x*,*t*) and *η*(*x*,*t*) that are continuous and have continuous first-order derivatives in the domains between the discontinuity characteristics (e.g. between lines *AC* and *CA*_1_, or *CA*_1_ and *A*_1_*C*_1_, in figure 3). The values of *η* and *γ* on these characteristics are discontinuous with the jump condition [46,47]

2.17 $$\eta^{+}-\eta^{-}=\pm c(\gamma^{+}-\gamma^{-})\;\left(\frac{\text{d}t}{\text{d}x}=\mp \frac{1}{c}\right),$$

where *η*^±^ and *γ*^±^ are the limits of *η*(*x*,*t*) and *γ*(*x*,*t*), respectively, obtained by approaching the discontinuity characteristics from different sides (indicated by pluses and minuses in figure 3).

As noted above, from the physical standpoint, the appearance of the discontinuity is a result of the simplified description (2.15) of the static-to-dynamic transition of the shear band. Such discontinuities are typical for this type of models [32,33,46,49], and the obtained solutions can be reasonably acceptable if the discontinuities are not too large and do not grow with time (§§3c and 4).

## 3. Solution

### (a) Band growth velocity and slip rate

We first consider the simplest case when the discontinuity emitted from the band tip, *x*=*l*_0_, reflects from *x*=0, but does not reach the band tip again (figure 3*a*). Integrating (2.16) along the characteristic line *BP* (figure 3*a*) results in

3.1 $$\gamma (P)-\frac{\eta (P)}{c}-\gamma^{+}(B)+\frac{\eta^{+}(B)}{c}=-\frac{1}{h}\int_{x_{B}}^{l_{P}}T\left(x,t_{B}+\frac{x-x_{B}}{c}\right)\text{d}x,$$

where *γ*^+^ (*B*) and *η*^+^(*B*) are the limits of *γ*(*x*,*t*) and *η*(*x*,*t*), respectively, when (*x*,*t*)→*B* from inside of *ACA*_1_ (figure 3*a*). To simplify notations, hereafter, *f*(*B*) means the value *f*(*x*_*B*_, *t*_*B*_) of function *f* in point *B*(*x*_*B*_, *t*_*B*_).

As the discontinuity propagates along *AC* (figure 3*a*), *γ*^−^(*B*) and *η*^−^(*B*) on this line are simply the initial static strain and material velocity (slip rate), respectively. They are undisturbed until the discontinuity reaches *x*_*B*_ at time *t*_*B*_=(*l*_0_−*x*_*B*_)/*c*. Therefore,

3.2 $$\gamma^{-}(B)=\gamma_{s}(x_{B})\;\text{and}\;\eta^{-}(B)=0,$$

and the jump condition (2.17) on the characteristic line *AC* becomes

3.3 $$\gamma^{+}(B)-\frac{\eta^{+}(B)}{c}=\gamma_{s}(x_{B}).$$

As point *P*(*l*_*P*_, *t*_*P*_) represents the propagating tip on plane (*x*, *t*), the second conditions in (2.14) and (2.15) apply. Substituting these conditions and (3.3) into (3.1), results in equation

3.4 $$\sqrt{\frac{1+v/c}{1-v/c}}=-\frac{\delta_{s}(x_{B},l_{P})}{\gamma_{c}},\;\delta_{s}(x_{B},l_{P})=\gamma_{s}(x_{B})-\frac{1}{h}\int_{x_{B}}^{l_{P}}T\left(x,t_{P}+\frac{x-l_{P}}{c}\right)\text{d}x,$$

for the band tip velocity, *v*=d*l*_*P*_/d*t*. This equation is to be solved for *l*_*P*_(*t*) using the initial condition *l*_*P*_(0)=*l*_0_. However, as *τ*_w_ in *T*=τ_*_/*E*=(*τ*_g_−*τ*_b_−*τ*_r_−*τ*_w_)/*E* depends upon slip velocity, it is not known *a priori* and needs to be found in the solution. Although the method of characteristics is suitable for finding *τ*_w_, it turns out that in most cases, the effect of *τ*_w_ is insignificant at the failure stage of the slide development (e.g. §8b). Therefore, we simplify the solution by assuming hereafter that *τ*_w_ in (2.4) is negligible (or independent of time). Then, *T*(*x*,*t*)=*T*(*x*) and using (2.10), we see that *δ*_*s*_(*x*_*B*_,*l*_*P*_)=*γ*_*s*_(*l*_*P*_) in (3.4). Hence, (3.4) rewrites as

3.5 $$\frac{v(l)}{c}=\frac{\gamma_{s}^{2}(l)-\gamma_{c}^{2}}{\gamma_{s}^{2}(l)+\gamma_{c}^{2}}\;(l_{0}\leq l<l_{A_{1}}),$$

where *l*=*l*_*P*_ for the arbitrary point *P* on the line, *x*=*l*(*t*), of the band tip locations (figure 3*a*).

Using (3.5) and solving equation d*l*/d*t*=*v*(*l*) with condition *l*(0)=*l*_0_, we find the arrival time

3.6 $$t(l)=\int_{l_{0}}^{l}\frac{\text{d}l}{v(l)}=\frac{l+l_{0}}{c}-\Delta t(l),\;\Delta t(x)=\frac{2}{c}\left[l_{0}-\gamma_{c}^{2}\int_{l_{0}}^{x}\frac{\text{d}l}{\gamma_{s}^{2}(l)-\gamma_{c}^{2}}\right],$$

of the band tip in location *x*=*l*. This expression also gives an implicit relation for *l*(*t*). The difference between the arrival times of the discontinuity and the band tip at point *x* is Δ*t* (figure 3*a*).

For *x*=*l*, equation (2.10), can be rewritten as

3.7 $$\gamma_{s}(l)=\gamma_{a}-\frac{l}{h}\bar{T}(l)=-\gamma_{0}-I(l),\;\bar{T}(l)=\frac{1}{l}\int_{0}^{l}T(x)\;dx\;\text{and}\;I(l)=\frac{1}{h}\int_{l_{0}}^{l}T(x)\;dx,$$

where $\bar{T}(l)$ is the average value of *T*(*x*) over the band of length, *l*. As *T*(*x*)>0, it follows from (3.7) that *γ*_*s*_(*l*)<0 for any *l*≥*l*_0_. Therefore, *δ*_*s*_(*x*_*B*_,*l*_*P*_)<0 in (3.4). Similarly, as *T*(*x*)>0, integral *I*(*l*) in (3.7) increases monotonically with *l* and so does the function *γ*^2^_*s*_(*l*) (as *γ*_0_>0). Initially, $\gamma_{s}^{2}(l_{0})=\gamma_{0}^{2}>\gamma_{c}^{2}$, so that $\gamma_{s}^{2}(l)>\gamma_{c}^{2}$ for any *l*≥*l*_0_. Hence, it follows from (3.5) that 0<*v*(*l*)<*c* and *v*(*l*) also grows monotonically with *l*. Finally, because of the monotonic increase of *γ*^2^_*s*_(*l*) with *l*, *t*(*l*) in (3.6) also monotonically increases with *l*. Hence, (3.5) and (3.6) give a physically meaningful solution for the dynamic growth of the shear band.

When the discontinuity does not reach the crack tip in physical space (figure 2), the corresponding characteristic line *CA*_1_ does not cross line *x*=*l*(*t*) of the tip location on the (*x*,*t*) plane (figure 3*a*). In this case, the mathematical limit of l→∞ is possible in (3.5) and (3.6), resulting in *v*→*c* as l→∞ and in l→∞ as t→∞. In reality, the shear band cannot become infinitely large. This issue will be addressed in §5. Until then, we formally allow *l* to be unbounded.

To obtain the strain, *γ*_*t*_, and slip rate, *η*_*t*_, at the band tip, we insert (3.5) into the second relation in (2.15) and use the second (compatibility) condition at the tip in (2.14). This gives

3.8 $$\gamma_{t}(l)=\frac{\gamma_{s}^{2}(l)+\gamma_{c}^{2}}{2\gamma_{s}(l)}\;\text{and}\;\frac{\eta_{t}(l)}{c}=-\frac{\gamma_{s}^{2}(l)-\gamma_{c}^{2}}{2\gamma_{s}(l)},$$

where, as shown above, *γ*^2^_*s*_(*l*) monotonically increases with *l*≥*l*_0_. Therefore, both |*γ*_*t*_(*l*)| and *η*_*t*_(*l*) increase as the shear band grows (*η*_*t*_(*l*)>0 as *γ*_*s*_(*l*)<0). Furthermore, comparing denominators in (3.5) and (3.8)) and noting that for *γ*_*c*_<−*γ*_*s*_(*l*)<1, inequality $\gamma_{s}^{2}(l)+\gamma_{c}^{2}<-2\gamma_{s}(l)$ is always satisfied, we see that *η*_*t*_(*l*)<*v*(*l*). Therefore, the model is physically consistent in the sense that the material at the band tip moves slower than the band tip itself.

When *t*→0 and *l*→*l*_0_, equations (3.5) and (3.8) yield at the initial moment

3.9 $$v(l_{0})=c\frac{n-1}{n+1},\;\eta_{t}(l_{0})=c\gamma_{0}\frac{n-1}{2n}\;\text{and}\;\gamma_{t}(l_{0})=-\gamma_{0}\frac{n+1}{2n},$$

where the last two expressions can also be obtained from (3.11) below with *t*_*Q*_→0 and *x*_*Q*_→*l*_0_.

The strain, *γ*, and slip rate, *η*, at the arbitrary point, *Q*(*x*_*Q*_,*t*_*Q*_), in domain *ACA*_1_ (i.e. below *CA*_1_ in figure 3*a*), are found by integrating (2.16) along characteristics *RQ* and *BQ*. The result is

3.10 $$\begin{array}{r} \gamma (Q)+\frac{\eta (Q)}{c}-\gamma (R)-\frac{\eta (R)}{c}=-\frac{1}{h}\int_{l_{R}}^{x_{Q}}T(x)\;\text{d}x\\ \text{and}\;\;\gamma (Q)-\frac{\eta (Q)}{c}-\gamma^{+}(B)+\frac{\eta^{+}(B)}{c}=-\frac{1}{h}\int_{x_{B}}^{x_{Q}}T(x)\;\text{d}x, \end{array}\}$$

where *γ*(*R*)=*γ*_*t*_(*l*_*R*_) and *η*(*R*)=*η*_*t*_(*l*_*R*_), because point *R*(*l*_*R*_, *t*_*R*_) is located on the band tip line, *x*=*l*(*t*) (figure 3*a*). Hence, combining (3.10) with (3.3) and using (3.8), we obtain that at point *Q*,

3.11 $$\gamma (Q)=\gamma_{s}(x_{Q})+\frac{\eta_{t}(l_{R})}{c}\;\text{and}\;\eta (Q)=\eta_{t}(l_{R}),$$

where *η*_*t*_(*l*_*R*_) is defined by (3.8) with *l*=*l*_*R*_. Because *QR* is a characteristic line with *R* being at the band tip (figure 3*a*), points *Q* and *R* are related by

3.12 $$l_{R}=x_{Q}-c(t_{R}-t_{Q})\;\text{and}\;t_{R}=t(l_{R}),$$

where function *t*(*l*) is given by (3.6). Substituting the first equation in (3.12) into the second defines *t*_*R*_(*x*_*Q*_, *t*_*Q*_). Then, the first equation in (3.12) gives *l*_*R*_(*x*_*Q*_, *t*_*Q*_) used in (3.11).

Finally, finding *γ* and *η* at the arbitrary point *K*(*x*_*K*_, *t*_*K*_) above line *CA*_1_ (figure 3*a*) can be done by integrating (2.16) along the characteristic lines *PK*, *HK* and *RH*. Lines *PK* and *RH* cross the discontinuity line *CA*_1_ (figure 3*a*). Given conditions (2.17), crossing the discontinuity line does not affect the result of the integration, however. Hence, similar to (3.10), we have

3.13 $$\begin{array}{r} \gamma (K)+\frac{\eta (K)}{c}-\gamma (P)-\frac{\eta (P)}{c}=-\frac{1}{h}\int_{l_{P}}^{x_{K}}T(x)\;\text{d}x,\\ \gamma (H)+\frac{\eta (H)}{c}-\gamma (R)-\frac{\eta (R)}{c}=-\frac{1}{h}\int_{l_{R}}^{x_{H}}T(x)\;\text{d}x\\ \text{and}\;\;\gamma (K)-\frac{\eta (K)}{c}-\gamma (H)+\frac{\eta (H)}{c}=-\frac{1}{h}\int_{x_{H}}^{x_{K}}T(x)\;\text{d}x, \end{array}\}$$

where *γ*(*R*)=*γ*_*t*_(*l*_*R*_), *η*(*R*)=*η*_*t*_(*l*_*R*_) and *γ*(*H*)=*γ*_*a*_ (per (2.14) as *x*_*H*_=0). Excluding *η*(*H*) then yields

3.14 $$\gamma (K)=\gamma_{s}(x_{K})+\frac{\eta_{t}(l_{P})-\eta_{t}(l_{R})}{c}\;\text{and}\;\eta (K)=\eta_{t}(l_{P})+\eta_{t}(l_{R}),$$

where *η*_*t*_(*l*_*P*_) and *η*_*t*_(*l*_*R*_) are defined by (3.8) written for *l*=*l*_*P*_ and *l*=*l*_*R*_, respectively. Using *t*(*l*) defined in (3.6), points *P* and *R* are related to point *K* in (3.14) by

3.15 $$\begin{array}{r} t_{H}=t_{K}-x_{K}/c,\;l_{R}=c(t_{H}-t_{R}),\;t_{R}=t(l_{R})\\ \text{and}\;\;l_{P}=x_{K}-c(t_{P}-t_{K}),\;t_{P}=t(l_{P}). \end{array}\}$$

For given *x*_*K*_ and *t*_*K*_, the first three equations give *l*_*R*_(*x*_*K*_, *t*_*K*_) and *t*_*R*_(*x*_*K*_, *t*_*K*_) (along with the auxiliary unknown *t*_*H*_(*x*_*K*_, *t*_*K*_)). The last two equations define *l*_*P*_(*x*_*K*_, *t*_*K*_) and *t*_*P*_(*x*_*K*_, *t*_*K*_).

If the discontinuity reaches the band tip (i.e. Δ*t*<0 as l→∞ in figure 3*a*; see also figure 5*a* in §4), then the consideration for region *ACC*_1_*A*_1_ in figure 3*b* remains identical to that in figure 3*a*. Hence, equations (3.11) with (3.12) and (3.14) with (3.15) can be used in domains *ACA*_1_ and *CA*_1_*C*_1_, respectively. Equations (3.5), (3.6) and (3.8) are valid until the discontinuity, propagating along *CA*_1_, reaches the band tip at point *A*_1_(*l*_*A*_1__,*t*_*A*_1__) (figure 3*b*). Employing again *t*(*l*) in (3.6), *l*_*A*_1__ and *t*_*A*_1__ are defined by

3.16 $$l_{A_{1}}+l_{0}=ct_{A_{1}}\;\text{and}\;t_{A_{1}}=t(l_{A_{1}}).$$

### (b) Recurrence solution

The solution in *ACC*_1_*A*_1_ in figure 3*b* was obtained using the values of *γ*^−^ and *η*^−^ on *AC* given in (3.2). In the same manner, the solution above *ACC*_1_*A*_1_ can be found by using functions *γ*^−^ and *η*^−^ on line *A*_1_*C*_1_ and considering *A*_1_ instead of *A* as a starting point. Functions *γ*^−^ and *η*^−^ are given by (3.14) when approaching *A*_1_*C*_1_ from *CA*_1_*C*_1_. Domains above line *A*_2_*C*_2_ in figure 3*c* can be treated similarly. As the initial values (3.2) of *γ*^−^ and *η*^−^ are known, this consideration shows the existence of the recurrence process depicted in figure 3*c*.

In general, the discontinuity may reach the band tip several times before this process truncates. Consider, therefore, the shear band propagation starting at a time of *t*_*A*_ when it has a length of *l*_*A*_, corresponding to point *A*(*l*_*A*_,*t*_*A*_) in figure 3*c*. In this case, region *ACC*_1_*A*_1_ can be interpreted as a general step in the recurrence process when the discontinuity either initiates at the band tip, *x*=*l*_0_, when *l*_*A*_=*l*_0_, *t*_*A*_=0, or reflects from the tip, *x*=*l*_*A*_, if *l*_*A*_>*l*_0_, *t*_*A*_>0. Our goal is expressing all unknown quantities in *ACC*_1_*A*_1_ through functions *γ*^−^(*x*,*t*) and *η*^−^(*x*, *t*) defined on *AC*. We do not specify *γ*^−^ and *η*^−^ at this point, but only assume that they are known either from the initial conditions (3.2) or computed at the previous step of the recurrence process.

We then use (2.17) instead of (3.3) in (3.1) and find the band growth velocity

3.17 $$\frac{v}{c}\equiv \frac{1}{c}\frac{\text{d}l_{P}}{\text{d}t}=\frac{{\left[\gamma_{s}(l_{P})+\delta^{-}(B)\right]}^{2}-\gamma_{c}^{2}}{{\left[\gamma_{s}(l_{P})+\delta^{-}(B)\right]}^{2}+\gamma_{c}^{2}}\;(l_{A}\leq l_{P}<l_{A_{1}}),$$

where *P* is the arbitrary point on segment *AA*_1_ of line *x*=*l*(*t*) of the band tip locations (figure 3*c*), and we take into account that *T*(*x*,*t*)=*T*(*x*). Points *B* and *P* in (3.17) are related by

3.18 $$x_{B}=\frac{l_{A}+l_{P}-c(t_{P}-t_{A})}{2}\;\text{and}\;t_{B}=t_{A}+\frac{l_{A}-x_{B}}{c},$$

where 0≤*x*_*B*_≤*l*_*A*_ and *t*_*A*_≤*t*_*B*_≤*t*_*A*_+*l*_*A*_/*c*. Henceforth,

3.19 $$\delta^{-}(B)\equiv \delta^{-}(x_{B},t_{B})=-\gamma_{s}(x_{B})+\gamma^{-}(B)-\frac{\eta^{-}(B)}{c}$$

is the value of function *δ*(*x*,*t*)=−*γ*_*s*_(*x*)+*γ*(*x*,*t*)−*η*(*x*,*t*)/*c* as point (*x*, *t*) approaches point *B*(*x*_*B*_, *t*_*B*_) on the discontinuity line *AC* from below *AC* (figure 3*c*).

The solution of the ordinary differential equation in (3.17) with condition *t*(*l*_*A*_)=*t*_*A*_ is denoted as *t*_1_(*l*). Once it is found, (3.17) provides tip velocity *v*(*l*) as a function of band length, *l*.

The strain, *γ*_*t*_, and slip rate, *η*_*t*_, at the band tip, *P*, are given by (2.14), (2.15) and (3.17) as

3.20 $$\gamma_{t}(P)=\frac{\gamma_{c}^{2}+{\left[\gamma_{s}(l_{P})+\delta^{-}(B)\right]}^{2}}{2[\gamma_{s}(l_{P})+\delta^{-}(B)]}\;\text{and}\;\frac{\eta_{t}(P)}{c}=\frac{\gamma_{c}^{2}-{\left[\gamma_{s}(l_{P})+\delta^{-}(B)\right]}^{2}}{2[\gamma_{s}(l_{P})+\delta^{-}(B)]},$$

where functions *x*_*B*_(*l*_*P*_, *t*_*P*_) and *t*_*B*_(*l*_*P*_, *t*_*P*_) are defined by (3.18).

The strain and slip rate at the arbitrary point, *Q*(*x*_*Q*_,*t*_*Q*_), in domain *ACA*_1_ (figure 3*c*) are obtained by combining (3.10) with (2.17) and (3.20). We have

3.21 $$\gamma (Q)=\frac{\eta (Q)}{c}+\delta^{-}(B)+\gamma_{s}(x_{Q})\;\text{and}\;\frac{\eta (Q)}{c}=\frac{\eta_{t}(l_{R})}{c}+\frac{\delta^{-}(D)-\delta^{-}(B)}{2},$$

where *δ*^−^(*B*) and *δ*^−^(*D*) are defined by (3.19). In (3.21), points *B*, *D* and *P* are related to point *Q* by the corresponding characteristic lines (figure 3*c*). That is, functions *l*_*R*_(*x*_*Q*_, *t*_*Q*_) and *t*_*R*_(*x*_*Q*_, *t*_*Q*_) in (3.21) are still defined by (3.12), but with *t*(*l*) replaced by *t*_1_(*l*). Then, for given *x*_*Q*_ and *t*_*Q*_, quantities *x*_*D*_, *t*_*D*_ and *x*_*B*_, *t*_*B*_ are found from

3.22 $$\begin{array}{r} x_{B}=\frac{l_{A}+x_{Q}-c(t_{Q}-t_{A})}{2},\;t_{B}=t_{A}+\frac{l_{A}-x_{B}}{c}\\ \text{and}\;\;x_{D}=\frac{l_{A}+l_{R}-c(t_{R}-t_{A})}{2},\;t_{D}=t_{A}+\frac{l_{A}-x_{D}}{c}. \end{array}\}$$

For the arbitrary point *K*(*x*_*K*_, *t*_*K*_) in *CA*_1_*C*_1_ (figure 3*c*), we again use (3.13), but with *γ*_*t*_ and *η*_*t*_ defined by (3.20) rather than (3.8). We then have

3.23 $$\begin{array}{r} \gamma (K)=\frac{\eta_{t}(l_{P})-\eta_{t}(l_{R})}{c}+\frac{\delta^{-}(B)-\delta^{-}(D)}{2}+\gamma_{s}(x_{K})\\ \text{and}\;\;\frac{\eta (K)}{c}=\frac{\eta_{t}(l_{P})+\eta_{t}(l_{R})}{c}+\frac{\delta^{-}(B)+\delta^{-}(D)}{2}, \end{array}\}$$

where *δ*^−^(*B*), *δ*^−^(*D*) and *η*_*t*_(*l*_*P*_),*η*_*t*_(*l*_*R*_) are defined by (3.19) and (3.20), respectively. In (3.23), points *K* and *B* are connected by characteristics *BP* and *PK,* whereas *K* is connected to *D* by characteristics *DR*, *RH* and *HK* (figure 3*c*). Hence, functions *x*_*D*_(*l*_*R*_, *t*_*R*_), *t*_*D*_(*l*_*R*_, *t*_*R*_) and *x*_*B*_(*l*_*P*_, *t*_*P*_), *t*_*B*_(*l*_*P*_, *t*_*P*_) are defined by (3.22) (last two equations) and (3.18), respectively, whereas *l*_*R*_(*x*_*K*_, *t*_*K*_), *t*_*R*_(*x*_*K*_, *t*_*K*_) and *l*_*P*_(*x*_*K*_, *t*_*K*_), *t*_*P*_(*x*_*K*_, *t*_*K*_) are given by (3.15) (with *t*_1_(*l*) instead of *t*(*l*)). Thus, *x*_*D*_, *t*_*D*_, *x*_*B*_ and *t*_*B*_ are defined for any given *x*_*K*_ and *t*_*K*_ from *CA*_1_*C*_1_.

Equations (3.21) and (3.23) enable finding *γ* and *η* at the arbitrary points in *ACA*_1_ and *CA*_1_*C*_1_, respectively (figure 3*c*). Equations (3.17) and (3.20) can be used until the discontinuity, propagating along *CA*_1_, arrives at the band tip at *A*_1_. Quantities *l*_*A*_1__ and *t*_*A*_1__ are defined by

3.24 $$c(t_{A_{1}}-t_{A})=l_{A_{1}}+l_{A}\;\text{and}\;t_{A_{1}}=t_{1}(l_{A_{1}})$$

instead of (3.16). To close the recurrence process, we note that *δ*^−^(*B*_1_) and *δ*^−^(*D*_1_) are the limits of *δ*(*K*) as *K*→*B*_1_ and *K*→*D*_1_, respectively (figure 3*c*). Hence, for the arbitrary points *D*_1_(*x*_*D*_1__,*t*_*D*_1__) and *B*_1_(*x*_*B*_1__,*t*_*B*_1__) on the discontinuity line *A*_1_*C*_1_ (figure 3*c*), we find from (3.23) and (3.19) that

3.25 $$\delta^{-}(D_{1})=-\delta^{-}(D)-\frac{2\eta_{t}(l_{R})}{c}\;\text{and}\;\delta^{-}(B_{1})=-\delta^{-}(B)-\frac{2\eta_{t}(l_{P})}{c}.$$

Here, points *D*, *R* and *D*_1_ are connected by the characteristics *DR*, *RH* and *HD*_1_, so that

3.26 $$\begin{array}{r} x_{D_{1}}=l_{A_{1}}-c(t_{D_{1}}-t_{A_{1}}),\;x_{D}=\frac{l_{A}+l_{R}-c(t_{R}-t_{A})}{2},\;t_{D}=t_{A}+\frac{l_{A}-x_{D}(l_{R},t_{R})}{c}\\ \text{and}\;\;t_{H}=t_{D_{1}}-\frac{x_{D_{1}}}{c},\;l_{R}=c[t_{H}-t_{R}(t_{H})],\;t_{R}=t_{1}(l_{R}) \end{array}\}$$

with *l*_*A*_1__ and *t*_*A*_1__ defined by (3.24). The first equation in (3.26) relates *x*_*D*_1__ and *t*_*D*_1__, whereas the last three equations give *t*_*H*_(*x*_*D*_1__,*t*_*D*_1__),*l*_*R*_(*x*_*D*_1__,*t*_*D*_1__) and *t*_*R*_(*x*_*D*_1__,*t*_*D*_1__). Once the latter two are found, the remaining two equations in (3.26) define *x*_*D*_(*x*_*D*_1__,*t*_*D*_1__) and *t*_*D*_(*x*_*D*_1__,*t*_*D*_1__) in (3.25). Expressions (3.26) can also be used for *δ*^−^(*B*_1_) in (3.25) by replacing *D*_1_, *H*, *R* and *D* with *B*_1_, *G*, *P* and *B*, respectively (figure 3*c*). More details are given in the electronic supplementary material, appendix B.

At the first step (*l*_*A*_=*l*_0_, *t*_*A*_=0), substituting conditions (3.2) into (3.19) gives

3.27 $$\delta^{-}(B)=\delta^{-}(D)=0,$$

which establishes the recurrence process for finding the solution everywhere. Specifically, (3.25) can be used to find the solution in *A*_1_*C*_1_*C*_2_*A*_2_ (figure 3*c*) directly from (3.17), (3.20), (3.21) and (3.23) simply by renaming the unknowns. For example, at the first step (figure 3*b*), (3.27) reduces the general equations (3.17), (3.20), (3.21) and (3.23) to (3.5), (3.8), (3.11) and (3.14), respectively. Considering *A*_1_ (defined by (3.16)) instead of *A*(*l*_0_, 0) as a starting point and using (3.27) with (3.20) (or (3.8)) in (3.25), we next find that at the second step,

3.28 $$\delta^{-}(D_{1})=-\frac{2\eta_{t}(l_{R})}{c}=\frac{\gamma_{s}^{2}(l_{R})-\gamma_{c}^{2}}{\gamma_{s}(l_{R})}\;\text{and}\;\delta^{-}(B_{1})=-\frac{2\eta_{t}(l_{P})}{c}=\frac{\gamma_{s}^{2}(l_{P})-\gamma_{c}^{2}}{\gamma_{s}(l_{P})}.$$

Then, (3.28) can be used with equations (3.17), (3.20), (3.21) and (3.23), which defines the second term of the recurrence process (i.e. in *A*_1_*C*_1_*C*_2_*A*_2_ in figure 3*c* when *l*_*A*_=*l*_0_ and *t*_*A*_=0 in *ACC*_1_*A*_1_). This process can continue indefinitely or until the recurrence process truncates at the step when Δ*t*≥0 as l→∞. This may happen already at the first step (figure 3*a*).

As common in the method of characteristics for hyperbolic equations [46,47], the solution of the partial differential equations (2.12) is reduced to solving the ordinary differential equation in (3.17). With condition *t*(*l*_*A*_)=*t*_*A*_, this equation has a unique solution, which, in some important cases (e.g. §§3a and 4), can be expressed in closed form. Some conclusions can be derived, however, even without explicitly solving (3.17).

For example, the evolution of the discontinuity in *γ* and *η* as it moves along characteristics *AC* and *CA*_1_ (figure 3) can be assessed by considering the limits of (3.21) and (3.23) when *Q*→*B*, *Q*→*M* and *K*→*M*, *K*→*B*_1_, respectively. In the general case of *l*_*A*_≥*l*_0_, *t*≥0, the corresponding discontinuity values can be written as (electronic supplementary material, appendix C)

3.29 $$\Delta \gamma (B)=\frac{\Delta \eta (B)}{c}=\Delta \gamma (M)=-\frac{\Delta \eta (M)}{c}=\frac{\eta_{t}(l_{A}+0)-\eta^{-}(A)}{c},$$

where Δ*γ*=*γ*^+^−*γ*^−^,Δ*η*=*η*^+^−*η*^−^,*B*∈*AC* and *M*∈*CA*_1_. Because the right-hand side in (3.29) is independent of *B* and *M*, discontinuities of *γ* and *η*/*c* are the same and do not change on characteristics *AC* and *CA*_1_. In other words, the discontinuity magnitude remains constant between reflections from the band tip. This magnitude, however, reduces after each tip reflection (electronic supplementary material, appendix C). Similarly, the band tip velocity, *v*(*l*), jumps when the tip is overtaken by the discontinuity. The velocity jump decreases, however, with each tip reflection, whereas the band velocity increases as the band grows.

Quantity *δ*^−^(*B*_1_)<0 in (3.25) at every step in the recurrence process. Hence, *δ*^−^(*B*)≤0 in (3.17), and the shear band remains subsonic (0<*v*(*l*)<*c*) for any *l*≥*l*_0_ (as *γ*_*s*_(*l*)<0). Because, *v*(*l*)>0, function *l*(*t*), defined by (3.17), monotonically increases with *t*. In addition, *γ*_*t*_(*l*)<0 and *η*_*t*_(*l*)>0 in (3.20), whereas *γ*_*s*_(*l*)<0. Further, the slip rate, *η*, at the arbitrary point in (3.21) and (3.23) is always positive. Finally, the argument used in §3a to show that *η*_*t*_(*l*)<*v*(*l*) for *η*_*t*_(*l*) and *v*(*l*) given by (3.5) and (3.8), respectively, also applies in the general case of (3.17) and (3.20). Thus, the obtained solution checks out from the physical standpoint.

In the following (§§4 and 5), we are mostly interested either in the discontinuity lagging behind the band tip or in the discontinuity reflected once from the tip (i.e. twice from *x*=0). These cases are practically important (e.g. §7) and illustrate all important features of the general solution, obtained above, for an arbitrary number of reflections.

### (c) Dimensionless parameters

The solutions presented above can be written in terms of dimensionless quantities

3.30 $$\Gamma =-\frac{\gamma }{\gamma_{0}},\;\Omega =\frac{\eta }{c\gamma_{0}},\;V=\frac{v}{c},\;\xi =\frac{x}{l_{0}}\;\text{and}\;\tau =\frac{ct}{l_{0}},$$

where the minus sign (in the first equation) corresponds to the normalized compressive strain being positive. It turns out that the obtained solution depends upon only two dimensionless parameters, that is, the ‘bluntness’ number, *n*, and the strain ratio,

3.31 $$\lambda_{*}=\frac{\gamma_{0}h}{T_{0}l_{0}}=\frac{\gamma_{0}}{\gamma_{a}+\gamma_{0}},$$

where $T_{0}=\bar{T}(l_{0})$ and function $\bar{T}(l)$ is defined by (3.7). For *γ*_*a*_≥0 (§2b) and *γ*_0_≥0, the range of parameter λ_*_ is 0≤λ_*_≤1. If 0≤*γ*_*a*_≪*γ*_0_,λ_*_≈1, and for *γ*_*a*_≫*γ*_0_,λ_*_≈0. Hence, below, we consider the range of 0≤λ_*_≤1. It is difficult to further narrow down this range without addressing the specifics of progressive growth of the shear band, until the band reaches the critical length of *l*_0_. As *l*_0_/*h*≫1, (3.31) implies that *γ*_*a*_+*γ*_0_=(*l*_0_/*h*)*T*_0_≫*T*_0_, where typically *T*_0_≪1 (*e*.*g*. §7).

## 4. Homogeneous loading

Homogeneous load distribution,

4.1 $$T(x)=T_{0}=\bar{T}(l_{0})=\text{const.}>0,$$

represents an important particular case [1,11,16–19] when the static strain (3.7) simplifies to

4.2 $$\Gamma_{s}(\lambda )=-\frac{\gamma_{s}(l)}{\gamma_{0}}=1+\frac{T_{0}(l-l_{0})}{h\gamma_{0}}=1+\frac{\lambda -1}{\lambda_{*}},$$

and the initial band length, *l*_0_/*h*=(*γ*_*a*_+*γ*_0_)/*T*_0_, is obtained by using (3.31).

In the normalized formulation (3.30), equation (3.5) rewrites as

4.3 $$V(\lambda )=\frac{\text{d}\lambda }{\text{d}\tau }=\frac{{\left[1+(\lambda -1)/\lambda_{*}\right]}^{2}-1/n}{{\left[1+(\lambda -1)/\lambda_{*}\right]}^{2}+1/n}.$$

Substituting (4.3) into (3.6) and integrating yields

**Figure 4.**
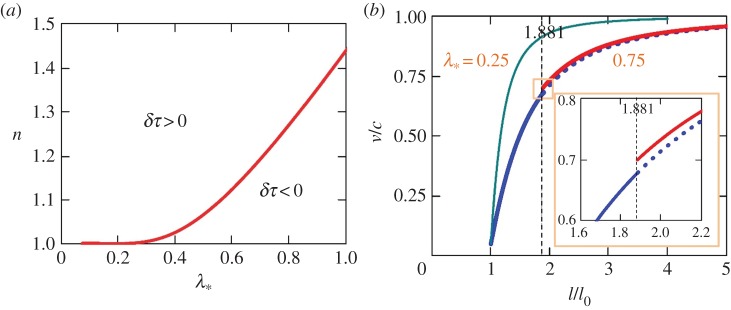
(*a*) Contour *δτ*=0 on the (λ_*_,*n*) plane for 0≤λ_*_≤1 and *n*>1. Regions *δτ*<0 and *δτ*≥0 correspond to the discontinuity that will and will not, respectively, reach the band tip. (*b*) Band growth velocity, *V* =*v*/*c*, as a function of band length, λ=l/l_0_, for *n*=1.1 and λ_*_=0.25 (equation (4.3); thin, green line, the discontinuity never reaches the band tip) and λ_*_=0.75 (bold, solid blue and solid red lines before and after the discontinuity reflects from the tip, respectively). Blue and red lines are plotted using (4.3) and (3.17) (with (3.28) and (4.2)), respectively.

4.4 $$\tau =\int_{1}^{\lambda }\frac{\text{d}\lambda }{V(\lambda )}=\lambda -1+\frac{\lambda_{*}}{\sqrt{n}}\left[\ln\frac{\sqrt{n}+1}{\sqrt{n}-1}+\text{ln}\frac{\lambda -1+\lambda_{*}\left(1-1/\sqrt{n}\right)}{\lambda -1+\lambda_{*}\left(1+1/\sqrt{n}\right)}\right],$$

which defines function *t*(*l*) (or *τ*(λ)) on *AA*_1_ (figure 3) and its inverse, *l*^−1^(*t*). Comparing (4.4) with (3.6) results in $\delta \tau =(c/l_{0}){\text{lim}}_{l\to \infty }\Delta t=2-(\lambda_{*}/\sqrt{n})\text{ ln }(1+2/(\sqrt{n}-1))$. If *δτ*≥0, the discontinuity will be ‘chasing’ the shear band tip, but will never catch up with it. In particular, when *n*≥1.4392, this takes place for the entire range of 0≤λ_*_≤1 (figure 4*a*).

Applying (4.2), expressions (3.8), (3.11) and (3.14) become elementary and, together with (4.3) and (4.4), provide the closed-form solution above line *AA*_1_ in figure 3*a* and in domain *ACC*_1_*A*_1_ in figure 3*b* in the case of homogeneous loading (4.1). The solution in *A*_1_*C*_1_*C*_2_*A*_2_ (figure 3*c* with *l*_*A*_=*l*_0_, *t*_*A*_=0) is then given by (3.17), (3.20), (3.21), (3.23) and (3.28) with *γ*_*s*_(*l*) defined by (4.2).

**Figure 5.**
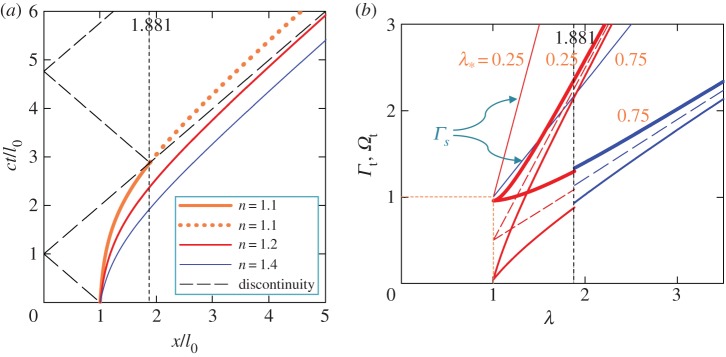
(*a*) Arrival time (equation (4.4)), *τ*=*ct*/l_0_, of the band tip at *ξ*=*x*/l_0_ for λ_*_=0.75 and *n*=1.4 (thin, blue line), *n*=1.2 (red line of medium thickness), and *n*=1.1 (bold, orange line). (*b*) Strain, *Γ*_*t*_=−*γ*_*t*_/*γ*_0_, and slip rate, *Ω*_*t*_=*η*_*t*_/(*cγ*_0_), at the band tip (solid lines, bold and of intermediate thickness, respectively) versus band length, λ=l/l_0_. *Γ*_*t*_ and *Ω*_*t*_ are plotted before (solid, red lines) and after (solid, blue lines) the discontinuity arrives at the tip for *n*=1.1 and λ_*_=0.25 (red lines) and 0.75 (red and blue lines). Thin, solid, red and blue lines show the static strain, *Γ*_*s*_=−*γ*_*s*_/*γ*_0_, for these values of λ_*_, respectively. Dashed lines show asymptotes of *Γ*_*t*_ and *Ω*_*t*_ at λ→∞.

Using (4.4) with λ=*ξ*, the location *ξ* of the shear band tip at time *τ* is plotted in figure 5*a* for λ_*_=0.75 and *n*=1.1, 1.2 and 1.4. To put these values of *n* in perspective, we note that according to (4.3), they correspond to an initial velocity *v*_0_ of the band tip equal to 4.8, 9.1 and 16.7% of *c*, respectively. The corresponding discontinuity characteristic lines are also plotted in figure 5*a*. As can be seen, the reflected discontinuity will catch up with the band tip for *n*=1.1 (at *ξ*_*A*_1__=*x*_*A*_1__/*l*_0_=1.881), but not for *n*=1.4. For *n*=1.2, the curves intersect at *ξ*=8.430, which is beyond the drawing domain in figure 5*a*. For λ_*_=0.75 and *n*≥1.04, the discontinuity reflects from the band tip at *ξ*≥10^3^. The value of *ξ*, where the discontinuity arrives to the band tip becomes larger with increasing *n* and decreasing λ_*_.

For λ_*_=0.75 and *n*=1.1, the velocity, *v*, of the band tip changes abruptly when the discontinuity reflects from the tip (figure 4*b*). The velocity jump, Δ*v*/*c*=2.153×10^−2^, is small though compared with the band velocities before, *v*/*c*=0.6777, and after, *v*/*c*=0.6992, the jump. Velocity *v* quickly approaches *c*, approximately within two or three initial band sizes (figure 4*b*). This observation prompts the existence of the asymptotic solution, where *v* reaches *c* already at *t*=0 (electronic supplementary material, appendix B). It holds for the entire range of 0≤λ_*_≤1 and *n*>1, and *v* approaches *c* faster for smaller λ_*_ and greater *n*. Note that although *v*→*c*, *v* remains smaller than *c*, which is in contrast with the result of Puzrin *et al.* [50]. Their band velocity is *unbounded* in time because they did not account for the emitted waves in their formulation.

According to (3.8), both tip strain, *Γ*_*t*_=−*γ*_*t*_/*γ*_0_, and tip slip rate, *Ω*_*t*_=*η*_*t*_/(*cγ*_0_), are larger for smaller λ_*_, and they both increase with λ starting from *Γ*_*t*_(1)=(*n*+1)/(2*n*) and *Ω*_*t*_(1)=(*n*−1)/(2*n*) (equations (3.9)), which are both independent of λ_*_. For *n*=1.1, the differences between *Γ*_*t*_(1)=0.9545 and 1 and between *Ω*_*t*_(1)=4.545×10^−2^ and 0 are hardly visible in figure 5*b*. As the band grows, both *Γ*_*t*_(λ) and *Ω*_*t*_(λ) monotonically grow approaching the same asymptote and remaining always greater and less than the asymptote, respectively (figure 5*b*). Both strain and slip rate at the band tip experience jumps, Δ*Γ*_*t*_=3.707×10^−2^ and Δ*Ω*_*t*_=5.384×10^−2^, when the tip is overtaken by the discontinuity (at *τ*=2.881). Although, the discontinuity reflects from *x*=0 for the second time (figure 5*a*), it will never reach the band tip again.

**Figure 6.**
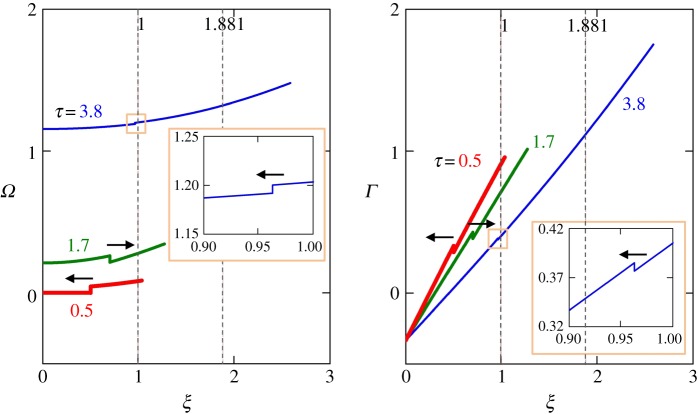
Distributions (for λ_*_=0.75 and *n*=1.1) of (*a*) slip rate, *Ω*=*η*/(*cγ*_0_) and (*b*) strain, *Γ*=−*γ*/*γ*_0_, along the slope, *ξ*=*x*/l_0_, at three times, *τ*=*ct*/l_0_: before the discontinuity has reflected from *x*=0 (*τ*=0.5); after it reflected from *x*=0, but before it reached the band tip (*τ*=1.7) and after the reflection from the tip (*τ*=3.8; discontinuity is shown in the insets). Arrows indicate directions of the movement of the discontinuity, which does not change between tip reflections. The band tip propagation can be seen by observing where the curves end.

Distributions of strain, *Γ*, and slip rate, *Ω*, along the slope are shown in figure 6 for λ_*_=0.75, *n*=1.1 and dimensionless times *τ*=0.5 (i.e. before the discontinuity reaches the slide end, *x*=0) and *τ*=1.7 (i.e. after it reflects from *x*=0 at *τ*=1, but before it arrives at the band tip, *x*=*l*). At time *τ*=2 (not shown in figure 6), the discontinuity passes the initial position *ξ*=1 (or *x*=*l*_0_) of the band tip. By that time, however, the tip has already advanced to the new position of *ξ*=1.387.

Figure 6 also shows distributions of *Γ* and *Ω* along the slope for λ_*_=0.75 and *n*=1.1 at *τ*=3.8, after the discontinuity reflects from the shear band tip, *ξ*=λ_*A*_1__=1.881, at *τ*=*τ*_*A*_1__=2.881. The discontinuity magnitude is Δ_0_/*γ*_0_=4.545×10^−2^ (the same for *Ω* and *Γ* per (3.29)) before the reflection, but reduces more than five times to Δ_1_/*γ*_0_=8.382×10^−3^ after the reflection, and can reduce much more (electronic supplementary material, appendix D). Therefore, the magnitudes of the propagating discontinuities of *Γ* and *Ω* quickly reduce with each reflection from the band tip and quickly become much smaller than the *Γ* and *Ω* magnitudes, which grow with time. This is a general trend (electronic supplementary material, appendix C), which is independent of the choice of parameters and justifies allowing discontinuities in our model.

## 5. Slope failure

As the shear band propagates, the strain magnitude at the tip, *x*=*l*(*t*), increases until it reaches the critical value, *γ*_p_=(*p*_p_−*p*_0_)/*E*>0, when the slope material above the band tip fails. Per (2.2), this strain corresponds to the (passive) failure stress, *σ*(*l*_f_, *t*_f_)=−*p*_p_ (*p*_p_>0). At this point, the shear band can be visualized as turning abruptly towards the surface, which effectively ends its propagation at *x*=*l*_f_ when *t*=*t*_f_. We refer to this event as slope failure, and first obtain *γ*_*t*_(*l*_f_) from (3.8). In this case, the discontinuity has not reflected yet from the band tip (figures 3*b*,*c*), and condition *γ*_*t*_(*l*_f_)=−*γ*_p_ (*γ*_p_>0) of the slope failure can be expressed as

5.1 $$\frac{\gamma_{s}^{2}(l_{f})+\gamma_{c}^{2}}{2\gamma_{s}(l_{f})}=-\gamma_{p}.$$

In turn, (5.1) yields

5.2 $$\gamma_{s}(l_{f})=-\gamma_{p}\pm \sqrt{\gamma_{p}^{2}-\gamma_{c}^{2}},$$

where, in the case of homogeneous loading (4.1), the static strain *γ*_*s*_(*l*_f_)=−*γ*_0_−*T*_0_(*l*_f_−*l*_0_)/*h* is defined by (4.2) with *l*=*l*_f_. Substituting this *γ*_*s*_(*l*_f_) in (5.2), solving the resulting equation for *l*_f_, and using (2.11), we obtain

5.3 $$\frac{l_{f}}{l_{0}}=1+\lambda_{*}\left[\frac{\gamma_{p}}{\gamma_{0}}-1+\sqrt{\frac{\gamma_{p}^{2}}{\gamma_{0}^{2}}-\frac{1}{n}}\right].$$

Here we assumed that the slope failure does not occur for *l*≤*l*_0_, which implies that *γ*_p_>*γ*_0_ (otherwise, the slope failure would occur before the shear band starts propagating dynamically). As *n*>1, the square root in (5.3) is a real number. Because *l*_f_/*l*_0_>1, we chose the minus sign in (5.2), which corresponds to the plus sign before the square root in (5.3).

For the general step of the recurrence process (before or after the discontinuity reaches the band tip), the left-hand side in (5.1) needs to be replaced by the tip strain (3.20) with *l*_*P*_=*l*_f_. Then, expression (5.2) becomes

5.4 $$\gamma_{s}(l_{f})=\delta^{-}(B)-\gamma_{p}\pm \sqrt{\gamma_{p}^{2}-\gamma_{c}^{2}},$$

and the failure length,

5.5 $$\frac{l_{f}}{l_{0}}=1+\lambda_{*}\left[\frac{\gamma_{p}}{\gamma_{0}}-1+\sqrt{\frac{\gamma_{p}^{2}}{\gamma_{0}^{2}}-\frac{1}{n}}+\frac{\delta^{-}(B)}{\gamma_{0}}\right],$$

is obtained by solving (5.4). We selected again the minus sign in (5.4), because choosing plus would result in the mines sign before the square root in (5.5), which, in turn, would correspond to *l*_f_/*l*_0_<1 (as *δ*^−^(*B*)≤0). In (5.5), points *B* and *P* are on the same characteristic line, *BP* (figures 3*b*,*c*) with *l*_*P*_=*l*_f_. At the first recurrence step, *δ*^−^(*B*)=0, and (5.5) results in (5.3). At the second step, *δ*^−^(*B*)/*γ*_0_=*γ*_*s*_(*l*_*P*_)/*γ*_0_−*γ*_0_/(*nγ*_*s*_(*l*_*P*_)) (per (3.28)), where *l*_*P*_ is defined (together with *l*_f_) by equations (5.5) and *t*(*l*_*P*_)+(*l*_*P*_+*l*_f_)/*c*=*t*_1_(*l*_f_).

Parameter *l*_f_ is important because it determines the amount of material available for the slide post-failure stage. It cannot be evaluated based on the conventional limit equilibrium analysis for an infinite slope [2,19], but it can be estimated from the quasi-static analysis by neglecting the dynamic effect and assuming the strain is static at the tip of the growing band [10,15–21]. The corresponding length, *L*_f_, of the static (or progressively propagating) band at failure is obtained from the same tip condition *γ*_*s*_(*L*_f_)=−*γ*_p_. Using (4.2) then results in

5.6 $$\frac{L_{f}}{l_{0}}=1+\lambda_{*}\left(\frac{\gamma_{p}}{\gamma_{0}}-1\right),$$

where *γ*_p_>*γ*_0_ and, therefore, *L*_f_/*l*_0_>1.

**Figure 7.**
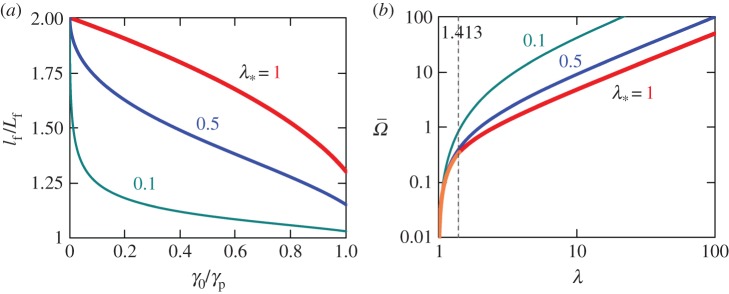
(*a*) Ratio of dynamic and static failure lengths, l_f_/*L*_f_, as a function of *γ*_0_/*γ*_p_ for *n*=1.1. For λ_*_=0.1 and 0.5, the discontinuity does not reach the band tip for all values of *γ*_0_/*γ*_p_. For λ_*_=1, the failure takes place after the discontinuity reflects from the tip (also for all values of *γ*_0_/*γ*_p_). In this case, there is only one reflection from the tip when λ=1.413 and *τ*=2.413. (*b*) Slide velocity, $\bar{\Omega }=\bar{\eta }/(c\gamma_{0})$, as a function of band length, λ=l/l_0_, plotted using (6.7) for *n*=1.1. For λ_*_=0.1 and 0.5, the discontinuity does not reach the band tip. For λ_*_=1, orange and red lines are used before and after the tip reflection, respectively.

For *γ*_p_ close to *γ*_0_, the slope failure takes place before the discontinuity reaches the band tip. In this case, (5.3) yields $l_{f}\text{/}l_{0}\approx 1+\lambda_{*}\sqrt{1-1\text{/}n}.$ Hence, for *n* close to 1, *l*_f_/*l*_0_ is only slightly greater than 1. For a large *n*, however, *l*_f_/*l*_0_≈1+λ_*_, which can be as large as 2 (as 0≤λ_*_≤1). Figure 7*a* shows *l*_f_/*L*_f_ as a function of *γ*_0_/*γ*_p_ plotted using (5.3), (5.5) and (5.6) for *n*=1.1 and λ_*_=0.1, 0.5 and 1. When *γ*_0_/*γ*_p_ is small, *l*_f_/*L*_f_ is also close to 2. For example, *l*_f_/*L*_f_>1.8 if *γ*_0_/*γ*_p_<0.2 and λ_*_>0.468 or *γ*_0_/*γ*_p_<0.1 and λ_*_>0.290. Therefore, parameter λ_*_ is important as it affects *l*_f_/*L*_f_ (figure 7*a*). The effect of *n* on *l*_f_/*L*_f_ is much weaker (§7c).

The reason of *l*_f_ being greater than *L*_f_ is that the elastic wave, emitted from the band tip at *t*=0, unloads the material, which is initially under the static strain, *γ*_*s*_(*x*). This can be seen in figure 5*b*, where both static and dynamic strain distributions along the band are plotted at the same scale. As a result, *γ*_*t*_(*l*)<*γ*_*s*_(*l*) and a larger *l*_f_ is required to satisfy the dynamic failure condition, *γ*_*t*_(*l*_f_)=−*γ*_p_, than the *L*_f_ satisfying the static failure condition *γ*_*s*_(*L*_f_)=−*γ*_p_.

## 6. Slide velocity

To evaluate the slide damage potential (and the potential to generate a tsunami in the case of submarine slides), the slide body velocity can be characterized by the length-averaged slip rate

6.1 $$\bar{\eta }(t)=\frac{1}{l}\int_{0}^{l}\eta (x,t)dx.$$

This quantity is called the ‘slide velocity’. In this section, we obtain $\bar{\eta }$ before the first and second reflection of the discontinuity from the band tip. These cases represent all important features of the general solution for $\bar{\eta }$ for an arbitrary number of reflections (electronic supplementary material, appendix B).

We first integrate the second equation in (2.12) with respect to *x* for *t*=*t*_*B*_≤*l*_0_/*c* and obtain

6.2 $$\frac{1}{c^{2}}\int_{x_{B}}^{l}\frac{\partial \eta (x,t)}{\partial t}\text{d}x=\int_{0}^{l}\frac{\partial \gamma (x,t)}{\partial x}\text{d}x+\frac{1}{h}\int_{0}^{l}T(x)\;\text{d}x,$$

where *x*_*B*_=*l*_0_−*ct* (figures 3*a*,*b*). Changing the order of integration and differentiation in the left-hand side and taking into account that both *l* and *x*_*B*_ are functions of *t* yields

6.3 $$\frac{\partial }{\partial t}\int_{x_{B}}^{l}\eta (x,t)\;\text{d}x=v(l)\eta_{t}(l)+c\eta^{+}(x_{B},t)+c^{2}[\gamma_{t}(l)-\gamma_{s}(l)+\gamma_{s}(x_{B})-\gamma^{+}(x_{B},t)],$$

where we used (2.10), (3.7) and that *γ*(*l*,*t*)=*γ*_*t*_(*l*), *η*(*l*,*t*)=*η*_*t*_(*l*) given by (3.8). Using the jump condition (3.3) and integrating (6.3) with respect to time results in

6.4 $$\int_{0}^{l}\eta (x,t)\;\text{d}x=\int_{l_{0}}^{l}\eta_{t}(l)\;\text{d}l+c^{2}\int_{l_{0}}^{l}\frac{\gamma_{t}(l)-\gamma_{s}(l)}{v(l)}\text{d}l,$$

where d*l*=*v*d*t* and *η*(*x*,*t*)=0 for 0≤*x*<*l*_0_−*ct*. Substituting (6.4) into (6.1) and using (3.5) and (3.8) gives

6.5 $$\frac{\bar{\eta }(l)}{c}=-\frac{1}{l}\int_{l_{0}}^{l}\gamma_{s}(l)dl\;(0<t\leq l_{0}/c).$$

For *l*_0_/*c*<*t*<(*l*_0_+*l*_*A*_1__)/*c*, where *l*_*A*_1__ is defined by (3.16), the integration interval is the horizontal line that intersects line *CA*_1_ (figure 3*b*). Otherwise, consideration is similar to the case of time *t*=*t*_*B*_≤*l*_0_/*c* and results in the same expression (6.5) for $\bar{\eta }(l)$ (electronic supplementary material, appendix B). Therefore, (6.5) is valid for the entire time interval, 0<*t*<(*l*_0_+*l*_*A*_1__)/*c*, before the first discontinuity arrival at the band tip (*A*_1_ in figure 3*b*).

For the time interval between the first and second arrivals of the discontinuity at the band tip (i.e. between points *A*_1_ and *A*_2_ in figure 3*c* when *l*_*A*_=*l*_0_, *t*_*A*_=0), a similar analysis yields

6.6 $$\frac{\bar{\eta }(l)}{c}=-\frac{1}{l}\int_{l_{0}}^{l}\gamma_{s}(l)dl+\frac{1}{l}\int_{l_{A_{1}}}^{l}\left[\frac{\eta_{t}(l)}{c}+c\frac{\gamma_{t}(l)-\gamma_{s}(l)}{v(l)}+\gamma_{s}(l)\right]dl\;(t_{A_{1}}<t<t_{A_{2}}),$$

where *v*(*l*) is given by (3.17), *t*_*A*_1__ and *l*_*A*_1__ are defined by (3.16), and, according to (3.24), *t*_*A*_2__ is defined by equations *c*(*t*_*A*_2__−*t*_*A*_1__)=*l*_*A*_2__+*l*_*A*_1__ and *t*_*A*_2__=*t*_1_(*l*_*A*_2__). Comparing (6.6) with (6.5), we observe that although the band growth velocity jumps at point *A*_1_ owing to the reflection of the discontinuity from the band tip (figure 4*b*), the slide velocity, $\bar{\eta }(l)$, remains continuous at *l*=*l*_*A*_1__.

Equation (6.4) is also valid for the arbitrary recurrence step (electronic supplementary material, appendix B). Together with (6.1), it represents the average slide velocity at the arbitrary time.

For homogeneous distribution (4.1) of *T*(*x*), expressions (6.5) and (6.6) can be combined as

6.7 $$\begin{array}{r} \bar{\Omega }(\lambda )=\frac{\bar{\eta }(l)}{c\lambda_{0}}=\left\{ \begin{array}{c} \frac{\lambda -1}{\lambda }\left(1+\frac{\lambda -1}{2\lambda_{*}}\right)\;(1<\lambda <\lambda_{A_{1}})\\ \frac{1}{\lambda }\int_{\lambda_{A_{1}}}^{\lambda }\left[\Omega_{t}(\lambda )-\Gamma_{s}(\lambda )-\frac{\Gamma_{t}(\lambda )-\Gamma_{s}(\lambda )}{V(\lambda )}\right]d\lambda +\frac{\lambda -1}{\lambda }\left(1+\frac{\lambda -1}{2\lambda_{*}}\right)\;(\lambda_{A_{1}}<\lambda <\lambda_{A_{2}}), \end{array}\right. \end{array}$$

where *Γ*_*t*_(λ)=−*γ*_*t*_(λ)/*γ*_0_, *Ω*_*t*_(λ)=*η*_*t*_/(*cγ*_0_), *V* (λ)=*v*/*c* and λ_*A*_1__=*l*_*A*_1__/*l*_0_ are defined by (3.27), (3.17), (3.20) and (3.16) with *γ*_*s*_(*l*) defined by (4.2). Dependence $\bar{\Omega }(\lambda )$ is shown in figure 7*b* for *n*=1.1 and λ_*_=0.1, 0.5 and 1. For these parameters, the discontinuity lags behind the tip after the first (for λ_*_=0.1 and 0.5) or second (for λ_*_=1) reflection from *x*=0. Before the discontinuity arrives at the band tip, *A*_1_, $\bar{\Omega }(\lambda )$ in (6.7) is independent of *n*, but the position of *A*_1_ itself (or the value of λ_*A*_1__) does depend upon *n* and so does $\bar{\Omega }(\lambda )$ in (6.7) (i.e. after the reflection from the tip). In figure 7*b*, for λ ranging from 1 to 10^2^ and λ_*_=0.1, 0.5 and 1, $\bar{\Omega }(\lambda )∼10^{-2}$ to 10^2^. For *γ*_0_∼0.001–0.01, this implies that by the time the shear band increases in length by one to two orders of magnitude, the slide velocity may become ∼0.1*c* or even ∼*c*, although it remains smaller than *c*. Indeed, as shown in §3, *η*_*t*_(*l*)<*v*(*l*)<*c* when −*γ*_*s*_(*l*)<1. Hence, because *η*(*x*,*t*)<*η*_*t*_(*l*(*t*)) for *x*<*l* (e.g. figure 6*a*), we also see that $\bar{\eta }(l)<c$. Slide velocity, $\bar{\eta }(l)$ does not always reach a value of ∼0.1*c*∼10 m s^−1^ (§7), but if it does, such a (relatively high) velocity of displaced material may contribute to the tsunamigenic potential of a submarine slide.

Finally, it should be noted that neglecting *τ*_w_ overestimates the value of τ_*_ in (2.4), which increases *T*_0_ and, therefore, the slide velocity. This can be seen in figure 7*b*, where a larger slide velocity corresponds to a smaller value of λ_*_, which per (3.31), corresponds to a larger *T*_0_.

## 7. Examples

### (a) Slides in normally consolidated sediments

Many, if not most, submarine slopes are composed of normally consolidated sediments. Hence, we first consider a landslide with *α*=6°, *h*=50 m, and material properties characteristic for normally consolidated sediments: *τ*_p_=0.25*σ*′_*v*_, *τ*_r_=0.4*τ*_p_, *E*=360*τ*_p_ and *ρ*_0_=1800 kg m^−3^, where *σ*′_*v*_=−*σ*′_*y*_=(*ρ*_0_−*ρ*_w_)*gh* cos *α* is the effective stress normal to the slope and the shear band (figure 2). Density of seawater *ρ*_w_≈1000 kg m^−3^. As *σ*′_*v*_ depends upon depth, so do *τ*_p_, *τ*_r_ and *E*. At the depth of *h*=50 m, *σ*′_*v*_=390 kPa, *τ*_p_=97.5 kPa and *τ*_r_=39.0 kPa. Below, thickness-averaged properties of the sliding layer, 0<*y*<*h*, are denoted by bars. We find ${\bar{\sigma }}_{v}^{\prime }=(1/2)\sigma_{v}^{\prime }=195.1\;$kPa, ${\bar{\tau }}_{p}=0.25{\bar{\sigma }}_{v}^{\prime }=48.8\;$kPa, $\bar{E}=360{\bar{\tau }}_{p}=17.6$ MPa, $c={\left(\bar{E}/\rho_{0}\right)}^{1/2}=98.8$ m s^−1^ and *τ*_g_(*h*)=(*ρ*_0_−*ρ*_w_)*gh* sin⁡α=41.0 kPa. In the landslide literature, the characteristic displacement, $\bar{\delta }$ in the tip zone ranges from 2 mm to 50 cm [1,9–11,16–19]. In the following, we use $\bar{\delta }=10$ cm. For these parameters, $J_{0}=(\tau_{p}-\tau_{r})\bar{\delta }=5.85$ kPa m, *γ*_0_=[2*J*_0_/(${h\bar{E})]}^{1/2}=0.365$%, and *T*_0_=(*τ*_g_−*τ*_r_)/$\bar{E}=1.13\times 10^{-4}$.

Failure of fully saturated, normally consolidated sediments under fast (dynamic) loading can be described by the Von Mises criterion, which results in $p_{a}={\bar{\sigma }}_{v}^{\prime }-2{\bar{\tau }}_{p}=97.5$ kPa and $p_{p}={\bar{\sigma }}_{v}^{\prime }+2{\bar{\tau }}_{p}=292.6$ kPa The corresponding average active and passive strains are *γ*_*a*_=(*p*_0_−*p*_*a*_)/$\bar{E}=0.11$% and *γ*_p_=(*p*_p_−*p*_0_)/$\bar{E}=1.00$%, respectively, where $p_{0}=K_{0}{\bar{\sigma }}_{v}^{\prime }\approx 0.6{\bar{\sigma }}_{v}^{\prime }=117.0$ kPa is the initial longitudinal stress in the sliding layer. The strain ratio, λ_*_=*γ*_0_/(*γ*_0_+*γ*_*a*_)=0.767, is found from (3.31), which also gives the critical length, *l*_0_=*h*(*γ*_0_+*γ*_*a*_)/*T*_0_=2.10 km.

As discussed in §4, a discontinuity reflected from *x*=0 would never catch up with the band tip for *n*≥1.4392 and the entire range of 0≤λ_*_≤1 (figure 4*a*). As the value of *n* only weakly affects band parameters and slide velocity at the time of slope failure (§8c), *n* can be replaced by any value greater than 1.4392, which simplifies the analysis. Hence, we chose *n*=1.5, and solution (5.3) for the failure length yields *l*_f_=9.11 km. Slide velocity, $\bar{\eta }=88.1$ cm s^−1^, is obtained from the first equation in (6.7), and it takes *t*_f_=97.4 s until the slope fails. In the static analysis (5.6), the failure length is only *L*_f_=4.90 km. Therefore, the dynamic-to-static length ratio, *l*_f_/*L*_f_=1.86. The ratio of *l*_0_/*h*=42.0 suggests that the condition of *l*_0_/*h*≫1 (§2a) is reasonably satisfied.

Thus, our dynamic analysis results in a slope failure length typical for many landslides [2–8,19]. It also shows that the static analysis underestimates this length by nearly a factor of two, which is in agreement with §5. This result is not significantly affected by the choice of $\bar{\delta }$. For example, changing $\bar{\delta }$ to 1 cm while keeping other parameters the same yields *l*_f_/*L*_f_=1.90. Increasing $\bar{\delta }$ to 0.5 m results in *l*_f_/*L*_f_=1.67, which also means a considerable underestimate of the slide size if the static approach is employed.

### (b) Gaviota and Humboldt slides

Gaviota slide (figure 1) [2,12,51–55] is located on a 4° slope composed of silty clay sediments. The depth of the Gaviota slide headwall is 365 m. The seafloor depression left by removing the sediment material is approximately 8 m deep, 1.65 km wide and 750 m long. Properties of the sediment from the Gaviota slide area were studied by Lee & Edwards [51] and Edwards *et al.* [52] based on the gravity cores taken in six locations. Their results suggest an overconsolidation ratio [56] of ≈1.5 [51]. Such a value is relatively low and indicates that the sediment is lightly overconsolidated [57]. The gravity cores, however, only sampled 1.5 m of the upper sediment layer, which is deeper than the 0.5-m thick drape that accumulated after the slide [53], but is much shallower than the 8-m thick Gaviota slide body. As noted by Lee & Edwards [51], in the absence of geological information, it is difficult to conclude that all 8 m of the displaced materials were overconsolidated (albeit lightly). We, therefore, consider the Gaviota slide sediment as being normally consolidated (typical for submarine deposits). This is consistent with the nearly constant regional sedimentation rate during the last 136 kyr [58], including the most recent 1000 years when the Gaviota slide occurred.

Adopting the same sediment properties as in the previous section, we find that because the slope angle is low (*α*=4°), the gravitational load, *τ*_g_=4.38 kPa, is now smaller than the maximal residual friction, *τ*_r_=6.26 kPa, so the slide would not take place at all. Submarine landslides, however, have occurred on slopes less than 1° [2–4]. This is commonly explained by the excess pore pressure, Δ*P*, that develops in the sediment (on the slip surface) by or at the time of the event [30,37]. Excess pore pressure is attributed to such factors as seismic load [2,27], methane-hydrate dissociation [4,28,29], fast sedimentation rates [3,11] and high artesian pressure [5]. Regardless of the physical nature of Δ*P* during the Gaviota slide event, we simply assume that the excess pore pressure acts only in the ‘weak’ plane where the shear band develops. To trigger the band growth and for the band to propagate the observed distance of *l*_f_≈750 m, an excess pressure Δ*P*=24.6 kPa is required in the shear band. In this case, *σ*′_*v*_ on the band place becomes *σ*′_*v*_=(*ρ*_0_−*ρ*_w_)*gh* cos *α*−Δ*P*=38.0 kPa, whereas Δ*P* does not affect *σ*′_*v*_=(*ρ*_0_−*ρ*_w_)*gh* cos *α*=62.6 kPa just above the band. Hence, ${\bar{\sigma }}_{v}^{\prime }=(1/2)(\rho_{0}-\rho_{w})gh\cos\alpha =31.3$ kPa, and we further compute *τ*_p_=0.25*σ*′_*v*_=9.50 kPa, *τ*_r_=0.4*τ*_p_=3.80 kPa, ${\bar{\tau }}_{p}=0.25\;{\bar{\sigma }}_{v}^{\prime }=7.83\;$kPa, $\bar{E}=360\;{\bar{\tau }}_{p}=2.82\;$MPa, $c={\left(\bar{E}/\rho_{0}\right)}^{1/2}=39.6$ m s^−1^, *τ*_g_=(*ρ*_0_−*ρ*_w_)*gh* sin *α*=4.38 kPa, $J_{0}=(\tau_{p}-\tau_{r})\bar{\delta }=0.570$ kPa m, *γ*_0_=[2*J*_0_/(${h\bar{E})]}^{1/2}=0.711$% and *T*_0_=(*τ*_g_−*τ*_r_)/$\bar{E}=2.05\times 10^{-4}$. Further, similar to §7a, $p_{a}={\bar{\sigma }}_{v}^{\prime }-2{\bar{\tau }}_{p}=15.7\;\mathrm{kPa}$, $p_{p}={\bar{\sigma }}_{v}^{\prime }+2{\bar{\tau }}_{p}=47.0\;\mathrm{kPa}$, $p_{0}=0.6{\bar{\sigma }}_{v}^{\prime }=18.8$ kPa, $\gamma_{a}=(p_{0}-p_{a})/\bar{E}=0.11$%, *γ*_p_=(*p*_p_−*p*_0_)/$\bar{E}=1.00$% and λ_*_=*γ*_0_/(*γ*_0_+*γ*_*a*_)=0.865. The critical length, *l*_0_=*h*(*γ*_0_+*γ*_*a*_)/*T*_0_=321 m. Equation (5.3) results in *l*_f_=752 m (for *n*=1.5) and the first equality in (6.7) yields $\bar{\eta }=28.6$ cm s^−1^. Our model suggests a time to slope failure of *t*_f_=20.2 s. Finally, in the static analysis, the band length (equation (5.6)) would only be *L*_f_=434 m. Hence, the dynamic-to-static failure length ratio is *l*_f_/*L*_f_=1.73, which shows that the static analysis considerably underestimates the failure length and the *in situ* volume of the removed material.

Adjacent to the Gaviota slide, a large fracture traverses the intact slope (figure 1). The fracture is approximately 8 km long eastward [12,53,55], resembles a headwall of the Gaviota slide [55], and probably formed concurrently with the Gaviota slide [54]. This fracture can be interpreted [13] as being produced by the developing rupture surface with the fracture representing the future location of the headscarp of the potential slide. A similar interpretation was offered by Bernander [5], who described the formation of long cracks on the ground surface with no generation of slope failure. Bernander [5] termed such cases ‘unfinished landslides’, which are also referred to as ‘confined failures’ [59]. We, therefore, hypothesize that this landslide did not take place at the time of the Gaviota slide because of a slight difference in the slope angle below the headscarp fracture. Assuming the same sediment properties in the two areas (as they are adjacent each other), we slightly change the slope angle from *α*=4° to 3.8°. This results in an increase of the critical length from *l*_0_=321 to 519 m. Hence, it is possible that when the initial rupture surface (shear band) reached the critical length, 321 m, under the sediment displaced by the Gaviota slide, it was still shorter than the critical length, 519 m, corresponding to the adjacent east slope. As a result, the unstable shear band growth did not occur to the east of the Gaviota slide. Therefore, the slight difference in slope angle may have caused a drastically different behaviour of the shear band (i.e. unstable, dynamic growth versus stable, static development). If the shear band keeps propagating progressively below the headscarp fracture, at some point, it may start propagating dynamically and will cause a considerably larger landslide of the length of *l*_f_=1.22 km (assuming the same Δ*P*). Thus, it may be beneficial to set up continuous monitoring of the east slope (between the Gaviota and Goleta slides [53]).

Another example of an ‘unfinished’ landslide is given by the Humboldt slide located in the Eel River basin on the Northern California continental margin [12,26]. Seismic profiling indicates a possible basal (sole) shear rupture that roughly parallels the slope of *α*≈2° at the depth of *h*≈65 m for *l*≈4.5 km in the downhill direction [26]. The slide origin has created a controversy because the slide has been interpreted, using the same data, as either a submarine slope failure deposit [26] or as a field of migrating, current-controlled sediment waves [12]. These hypotheses can be reconciled, however, by noting that the sediment waves in the Humboldt slide area are relatively shallow, whereas the basal rupture develops much deeper. Although the initial rupture took place prior to 10 000 years ago, sediment movement may still be occurring [26]. The corresponding incremental propagations of the basal rupture may be caused by abundant seismic activity in the area assisted by the widespread gas presence in the sediments. It is, therefore, plausible that the rupture (shear band) has been propagating progressively, and the catastrophic propagation is yet to take place. If the current length, *l*, of the basal shear rupture is close to the critical value, *l*_0_, and the excess pressure in the rupture is sufficiently high, the rupture may begin to propagate catastrophically. For the above sediment properties and Δ*P*=341.4 kPa, this would result in the sediment ‘slab’ of *l*_f_≈32 km long (and 65 m thick), which nearly doubles the anticipated value of *L*_f_≈17 km (obtained in the static approximation). The subsequent motion of such a slab may generate a considerable tsunami.

### (c) Slides in sensitive clays

In sensitive clays, gently sloping landslides may take place even in the absence of excess pore pressure. Sensitive clays are characterized by rapid strength decrease during deformation [60], which translates to a low friction coefficient. Quinn *et al.* [16], for example, suggest *τ*_r_/*τ*_p_=0.013 for subaerial sensitive clays in Quebec area (Canada). Sensitivity of the sediment material appears to be a major factor in the deformation-softening process [5], and promotes strain localization and propagation of shear bands [8]. As a result, many landslides occurred in slopes composed of sensitive clays [16,60–62]. Hence, we also considered a scenario when a landslide is caused by reducing the frictional resistance not by rising pressure, but by mobilizing soil sensitivity in a thin zone where the rupture surface (shear band) develops. For the parameters in §7a, reducing *τ*_r_/*τ*_p_ to 0.013 [16] allows the slide to develop when *α*=0.5°. This results in *l*_0_=1.07 km, *l*_f_=8.64 km, *t*_f_=87.4 s, $\bar{\eta }=92.9$ cm s^−1^ and *l*_f_/*L*_f_=1.89.

## 8. Discussion

### (a) Dynamic version of the Palmer & Rice [1] model

**Figure 8.**
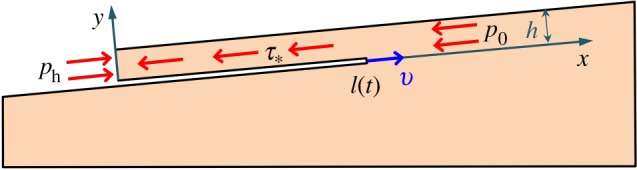
Dynamic version of the Palmer & Rice's [1] model for a slip surface in open-cut slope.

Palmer & Rice [1] analysed a static shear band developing uphill in an open-cut slope (figure 8) in an overconsolidated sediment. Here, we consider a dynamic band growing uphill in such a slope (figure 8). The atmospheric (for subaerial slides) or hydrostatic (for submerged slides) thickness-averaged pressure, *p*_*h*_>0, acts at the bottom of the cut, so that *σ*=−*p*_*h*_ at *x*=0. Per (2.2), *γ*_*h*_=*p*_0_/*E*>0 is the strain that corresponds to *p*_*h*_. Initially, *γ*_0_<*γ*_*a*_, and the band propagates until the strain at the tip reaches the (active) failure strain, which is now *γ*_*a*_=(*p*_0_−*p*_*a*_)/*E*. Because the strain magnitude increases uphill, condition 0<*γ*_*h*_<*γ*_0_<*γ*_*a*_ should be satisfied as in the static case [1].

Uphill (figure 8) and downhill (figure 2) propagations differ by the relative direction of τ_*_ and the *x*-axis, which now points uphill (figure 8), and by *p*_*h*_ acting at *x*=0 instead of *p*_*a*_. Hence, dynamic analysis of uphill growth follows directly from the results for downhill growth. Specifically, keeping both τ_*_ and *T* positive, we simply need to replace the signs before these quantities in (2.3), (2.12) and (2.16) and adjust other equations accordingly. In particular, the sign before *T* in (3.1) needs to be changed, and the static strain (2.10) becomes

8.1 $$\gamma_{s}(l)=\gamma_{h}+\frac{l}{h}\bar{T}(l)=\gamma_{0}+I(l),$$

with $\bar{T}(l)$ and *I*(*l*) given in (3.7). Expressions (3.5) and (3.17) for the band growth velocity, (3.8) and (3.20) for the tip strain and slip rate, and (6.5) and (6.6) for the slide velocity remain the same, but they should be used with the adjusted static strain (8.1). The position of the band tip is still defined by (3.6) and (3.17) (both used with (8.1) for *γ*_*s*_(*l*)). The location of point *A*_1_ of the discontinuity arrival at the band tip (figures 3*b*,*c*) is given by (3.16) or (3.24). Instead of (3.31), the strain ratio λ_*_ is now defined by

8.2 $$\lambda_{*}=\frac{\gamma_{0}h}{T_{0}l_{0}}=\frac{\gamma_{0}}{\gamma_{0}-\gamma_{h}}.$$

The recurrence relations (3.17), (3.20), (3.21) and (3.23) are also all valid if the definitions of *γ*_*s*_ and λ_*_ are adjusted using (8.1) and (8.2), respectively. Because 0<*γ*_*h*_<*γ*_0_, definition (8.2) affects the range of λ_*_, which is now λ_*_≥1 instead of 0≤λ_*_≤1 in the case of downhill growth.

For the homogeneous distribution (4.1) (i.e. $T=T_{0}=\bar{T}(l_{0})=\text{const.}>0)$, equations (4.3), (4.4) and (6.7) all remain valid, but with the static strain in (4.2) replaced by *γ*_*s*_(*l*)=*γ*_0_+*T*_0_ (*l*−*l*_0_)*h* (i.e. changing sign in (4.2)). The initial band length is now given by *l*_0_/*h*=(*γ*_0_−*γ*_*h*_)/*T*_0_, which agrees with Palmer & Rice [1] for *γ*_*h*_=0. The discontinuity reaches the tip for any *n*>1 when λ_*_>1.605 and for any λ_*_>1 when 1<*n*<1.440. As *γ*_*h*_>0, a shorter initial band length, *l*_0_, is required for uphill propagation (equation (8.2)) than for downhill propagation (equation (3.31)). In turn, for a shorter initial length, the discontinuity will travel more frequently between *x*=0 and the band tip, so more reflections are expected for an uphill developing band. Similar to downhill growth, the sliding layer above the shear band is loaded (strained) less in the dynamic than in the static condition. Hence, like in the downhill scenario, the dynamic failure length, *l*_f_, is again expected to be larger than for statically (progressively) growing band.

The dynamic version of the Palmer & Rice's [1] model can be used to analyse the uphill growth of the shear bands inferred from observations [5,9,16–18,63]. The analysis of the slide body size and velocity at slope failure is similar to that in §§5 and 6. Specifically, (5.3), (5.5) and (6.7) (with λ=λ_f_=*l*_f_/*l*_0_) remain valid if *γ*_p_ in these equations is replaced with *γ*_*a*_=(*p*_0_−*p*_*a*_)/*E*, and the static strain is replaced by *γ*_*s*_(*l*_f_)=*γ*_0_+*T*_0_(*l*_f_−*l*_0_)/*h*. Examples are given in the electronic supplementary material (appendix D).

### (b) Effect of water resistance

For a thin layer (figure 2), the slope surface velocity is equal to the slip rate, *η*. Hence, for submarine slides, the water resistance, *τ*_w_, on the slope surface is a function of the local value of *η*. To the first order, however, the effect of *τ*_w_ can be estimated by assuming instead that $\tau_{w}=\tau_{w}(\bar{\eta })$, where the length-averaged velocity, $\bar{\eta }(t)$, of the sliding layer is defined by (6.1). Function $\tau_{w}(\bar{\eta })$ depends upon several factors such as slide geometry, character of flow (turbulent or laminar) and slide surface material (e.g. sand or clay). Before the slope failure occurs and the slide body begins separating from the substrata, the seafloor surface is relatively flat, and the Stokes drag is not significant. In this case, $T_{w}(\bar{\eta })=\tau_{w}(\bar{\eta })/E=\beta {\bar{\eta }}^{2}/(\gamma_{0}^{2}c^{2})$ where $\beta =C_{S}\rho_{w}\gamma_{0}^{2}/(2\rho_{0}),$ and the surface (skin) friction coefficient, *C*_*S*_, depends upon the local Reynolds number. In the submarine slope analysis, however, such as submerged landslides and seabed wave loading, a constant value of *C*_*S*_ is commonly used. Depending upon the slope surface conditions, this value can be as low as 0.003 [64]. For scaling purposes, we use *C*_*S*_=0.01, which probably provides a conservative estimate of the frictional drag associated with the (turbulent) boundary layer near the slope surface.

For most soils and submarine sediments, *ρ*_0_/(2*ρ*_w_)∼1 and we expect *γ*_0_ does not exceed ∼10^−2^ (§7). Hence, *β*∼10^−6^ (or smaller), whereas the first equation in (6.7) suggests that at slope failure, *T*_w_ scales as *T*_w_=*β*((*l*_f_−*l*_0_)/*l*_f_)^2^(1+(*l*_f_−*l*_0_)/(2*l*_0_λ_*_))^2^. According to this estimate, for *β*∼10^−6^, *l*_f_/*l*_0_≤5 and λ_*_≥0.5, quantity $T_{w}≲1.6\times 10^{-5}$. On the other hand, the driving force scales as *T*=*T*_0_=(*γ*_*a*_+*γ*_0_)*h*/*l*_0_ (§4). In many observed landslides, *l*_f_/*h*∼10^2^ to 10^3^, so inequality *l*_0_<*l*_f_ implies that $h\text{/}l_{0}=(h/l_{f})(l_{f}/l_{0})≳10^{-2}.$ Hence, for *γ*_0_∼10^−2^ it is likely that $T≳10^{-4}.$ Consequently, there exists a realistic range of parameters when *T*_w_≪*T*.

For example, for Gaviota slide conditions (figure 1) and parameters given in §7b, *β*=1.41×10^−7^ and *T*_w_=1.45×10^−7^, which is three orders of magnitude smaller than the driving force *T*=*T*_0_=2.05×10^−4^ estimated without accounting for *τ*_w_ (§7b). We conclude, therefore, that the effect of water resistance during the failure stage of the Gaviota slide was probably negligible. For a typical slide in normally consolidated sediments (§7a), *β*=3.70×10^−8^ and *T*_w_=2.21×10^−7^≪*T*_0_=1.13×10^−4^. Hence, the effect of water resistance is insignificant, unless λ and λ_*_ are relatively large and small, respectively.

### (c) Effects of bluntness parameter and frictional weakening

The value of the bluntness parameter, $n=J_{0}/J_{c}=\gamma_{0}^{2}/\gamma_{c}^{2},$ is not well constrained for sediment materials. Hence, we tested its effect in the case of homogeneous loading (§4). For a representative value of *γ*_0_/*γ*_p_=0.1, the effect of value of *n* on the slide velocity, $\bar{\eta }$, the failure length, *l*_f_, and the shear band growth velocity, *v*, does not exceed several per cent as *n* changes from 1.001 to ∞ (electronic supplementary material, appendix D). For larger values of *γ*_0_/*γ*_p_, the effect is larger, but even for *γ*_0_/*γ*_p_=0.5, the effect of *n* in this range does not exceed 10%. When *n* is relatively close to 1, its value is important with respect to the initial shear band and slip velocities. For example, when *n* changes from 1.1 to 1.2, the factor of *n*−1 in (3.9) doubles and so do the initial band velocity and slip rate. Yet, this does not significantly affect the slide body velocity at failure or the failure length. Therefore, although it is difficult to constrain parameter *n* without explicitly addressing the specifics of the friction mechanisms in the band, the band length and slip rate at slope failure only weakly depend upon *n* and can be considered independent of *n* for *n*>1.001.

To understand the shear band development, we employed different fracture (surface) energies for static and dynamically propagating shear bands (§2c), which resulted in *γ*_0_≠*γ*_*c*_. The difference between *γ*_0_ and *γ*_*c*_ may also be due to the difference in elastic moduli during the quasi-static and dynamic phases of the band growth. If these phases are drained and undrained, respectively, this would result in $n=\gamma_{0}^{2}/\gamma_{c}^{2}=(1-v)/(1-v_{u})\approx 1.3-1.5>1$ even when *J*_0_=*J*_*c*_.

**Figure 9.**
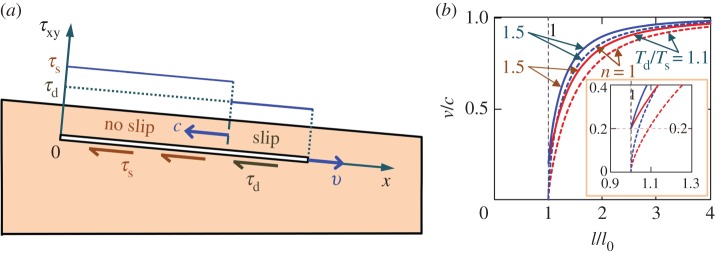
Shear band with frictional weakening. (*a*) The moving slip boundary (dashed line) separates regions with static, *τ*_*s*_, and dynamic, *τ*_*d*_, frictional tractions on the shear band sides. (*b*) Band growth velocity, *V* =*v*/*c*, as a function of band length, λ=l/l_0_, for *n*=1 (dashed lines) and 1.5 (solid lines) and *T*_*d*_/*T*_*s*_=1.1 (red lines) and 1.5 (blue lines). Orange and green labels indicate the values of *n* and *T*_*d*_/*T*_*s*_, respectively. In this example, the discontinuity does not reach the band tip (figure 3*a*).

An alternative way is to consider frictional weakening caused by the relative slip of the band sides. Such weakening can be envisioned, for example, on slip surfaces in rocks and rock-like (overconsolidated) sediments. To the first order, one can simply assume that the (residual) friction at a given place in the shear band reduces instantaneously when slip initiates at this place. Consequently, let *τ*_r_(*x*,*t*)=*τ*_*s*_(*x*) when $\bar{\eta }(x,t)=0$ and *τ*_r_(*x*,*t*)=*τ*_*d*_(*x*) when $\bar{\eta }(x,t)>0$, where *τ*_*s*_ and *τ*_*d*_ are the static and dynamic tractions, respectively, caused by the residual friction on the slip surface (figure 9*a*). For *τ*_*d*_<*τ*_*s*_, the driving load, *T*, increases, which, in essence, destabilizes the system and causes the dynamic growth. In this case, the solution, obtained in §3, needs only to be changed at the first step of the recurrence sequence. At the first step (figures 3*a*,*b*), the slip occurs at a part of the shear band (between the tip and a given point), whereas the other part of the band (between *x*=0 and this point) is still at rest. Although the initial condition *η*^−^(*B*)=0 (equation (3.2)) remains valid, quantities *γ*_*s*_(*x*_*B*_) and *γ*^−^(*B*) in (3.19) change. They are both defined by (3.7), but with *τ*_r_ in *T* (equation (2.4)) replaced by *τ*_*s*_ and *τ*_*d*_, respectively. Specifically, $\gamma_{s}(x)=\gamma_{a}-(1/h)\;\int_{0}^{x}T_{d}\;\text{d}x$ and *γ*^−^(*B*)=*ε*_*s*_(*x*_*B*_), where $\varepsilon_{s}(x)=\gamma_{a}-(1/h)\int_{0}^{x}T_{s}\;\text{d}x,\;T_{d}=(\tau_{g}-\tau_{b}-\tau_{d}-\tau_{w})\text{/}E$ and *T*_*s*_=(*τ*_g_−*τ*_b_−*τ*_*s*_−*τ*_w_)/*E*. In this case, *δ*^−^(*B*) is not zero anymore (equation (3.27)), but is defined by (3.19). All other steps of the recurrence solution (§3b) remain the same.

An example is shown in figure 9*b* for *T*_*d*_/*T*_*s*_=1.1 and 1.5, where *T*_*d*_ and *T*_*s*_ are the homogeneous driving loads that correspond to constant *τ*_*d*_ and *τ*_*s*_, respectively. As expected, there is no tip velocity jump at the initial moment if *n*=1 (or *J*_0_=*J*_*c*_) and *τ*_*d*_<*τ*_*s*_. For *n*>1, the tip velocity does jump, but the effect of this jump is only significant at relatively short times. It should be noted that although *v*(*l*) is a continuous function when *n*=1, the propagating discontinuity in *γ* and *η* is still present and has the same meaning as in the case of *n*>1.

### (d) Slides with varying slope angle and finite width

So far, we have considered slopes with constant angles (figure 2). If the slope angle, **α**, varies along the slope [65], but the angle is small (0≤*α*(*x*)≪1) and changes gradually (|*α*′(*x*)|≪1), the momentum balance condition can be written in the form of (2.3), or the second equation in (2.12), with *T*=τ_*_/*E*, τ_*_=(*ρ*_0_−*ρ*_w_)*gh* (tan*α*−*μ*)−*τ*_w_, and *x* understood as the curvilinear coordinate (length) along the shear band. The band parallels the slope at distance *h* in the direction perpendicular to the slope. The boundary and initial conditions (2.13)–(2.15) and the jump conditions (2.17) remain valid.

Because the solution obtained in §3 is valid for the general case of *T*(*x*), it is also valid for the above interpretation of *T*(*x*). As before, *p*_0_ is considered to be independent of *x*. Conditions 0≤*α*(*x*)≪1 and |*α*′(*x*)|≪1 can be formally realized, for example, by considering *α*(*x*)=*εβ*(*x*) and *α*′(*x*)=*εβ*′(*x*), where *ε* is a small parameter (say *ε*=*D*/*L*, where *D* is the vertical drop over a characteristic band length *L*) and function *β*(*x*)=*O*(1) defines the slope shape. Then, equation (2.3) is asymptotically accurate up to the (omitted) second-order terms *O*(*ε*^2^). In this approximation, varying slope angle affects only *T*(*x*) in (2.3) (or in (2.12)), where the first-order term tan*α*=*O*(*ε*) is kept. Keeping this term is significant, because for *α* reducing with *x*, the driving load τ_*_ may eventually become negative. Hence, the shear band may eventually stop before the slope failure takes place. This can be seen from (3.5), which suggests that the shear band stops growing when it reaches a length, *l*_s_, such that *γ*_s_(*l*_s_)=−*γ*_c_. Using (3.7), this condition can be written as

8.3 $$I(l_{s})=\frac{1}{h}\int_{l_{0}}^{l_{s}}T(x)\;\text{d}x=\gamma_{c}-\gamma_{0},$$

where *T*(*x*) decreases (and even becomes negative) with decreasing *α*. According to (3.8), both *η*_t_(*l*_s_)=0 and ∂*η*_t_(*l*_s_)/∂*t*=0 if (8.3) is satisfied. Therefore, both the slip rate and acceleration equal zero at the shear band tip when it reaches the length of *l*_s_. This indeed means the band growth has stopped, although it needs to be checked that the slide body motion also ceases and the growth would not restart later.

Using (3.7), equation (8.3) can be written as

8.4 $$l_{s}\bar{T}(l_{s})-l_{0}\bar{T}(l_{0})=(\gamma_{c}-\gamma_{0})h,$$

which further yields (for 0≤*α*(*x*)≪1 and |*α*′(*x*)|≪1)

8.5 $$H(l_{s})-H(l_{0})=\mu (l_{s}-l_{0})-\frac{(\gamma_{0}-\gamma_{c})E}{(\rho_{0}-\rho_{w})g},$$

where *H*(*x*) is the depth of the slope point, *x*, with respect to its highest (or any other) point. Because function $l\bar{T}(l)=\int_{0}^{1}T(s)\;ds$ monotonically grows with *l*, a lower bound of *l*_s_ is obtained by setting *γ*_0_=*γ*_c_ in (8.3) or (8.4). This bound is independent of *n* and is close to the actual value of *l*_s_ if *n* is close to 1 (i.e. if *γ*_c_ is close to *γ*_0_). Note that *I*(*l*_s_)=0 has another (trivial) solution, *l*_s_=*l*_0_, which is not of interest here.

Finally, many slides have an elongated shape [3,4,6,53], which typically depends upon the topographic features of the slide region. Yet the characteristic slide width, *b*, is usually much greater than the thickness, *h*, of the sliding layer. In most cases, the overall resistance to the slide movement (per unit area) at its margins (side scars) is comparable to the bottom (shear band) friction (also per unit area), although it may be much more complex in detail [13,22]. Therefore, if *b*≫*h*, the edge resistance at the side scars can be ignored to the first order, and if *b* does not change too much along the slope, the obtained solution can also be used for slides of finite width.

## 9. Conclusions

This work considers slides caused by a shear band that develops below the sliding mass along the potential slip (rupture) surface in long slopes. The material above the band slides downwards, causing the band to grow. This growth may first be stable (progressive), but eventually becomes dynamic (catastrophic). The corresponding dynamic problem for the shear band propagating subparallel to the slope surface is formulated within the framework of the Palmer & Rice's [1] approach, which is generalized to the dynamic case. Our results are applicable to both submarine and subaerial landslides of this type.

Using the method of characteristics, we found the exact, closed-form solution for the shear band velocity and slip rate distribution along the slip surface. The solution assesses when the displaced material separates from the substrata once a failure condition is satisfied near the tip of the propagating band. The solution is obtained for an arbitrary distribution of shear and gravitational forces along the slope. As an important example, the uniform distribution of these forces is considered in detail. In the case of a varying slope angle, we formulated a condition for cessation of the band growth.

To understand the shear band evolution, we employed different fracture energies or, alternatively, different residual frictions for static and dynamically propagating shear bands. This simplified approach captures, to the first order, the friction dependence on the slip rate. It results, however, in a strain discontinuity emitted from the band tip and travelling back and forth above the growing shear band. Yet, the magnitude of this discontinuity is small and quickly decreases with each reflection from the band tip. The developed model, therefore, appears to be physically acceptable. Using this model, we showed that the shear band accelerates, and the band tip velocity reaches the order of the speed, *c*, of elastic waves (yet remains smaller than *c*) after it propagates only approximately two or three lengths of the initial band. The slip rate also grows with the band length, yet remains smaller than the band growth velocity.

The band tip velocity, *v*, strain, *γ* and slip rate, *η*, are controlled by the ‘bluntness’ parameter, *n*, initial deformation, *γ*_0_, and strain ratio, λ_*_=*γ*_0_/(*γ*_*a*_+*γ*_0_). It turns out that *γ* and *η* are simply proportional to *γ*_0_, whereas the effect of *n* and λ_*_ is more complex. The value of λ_*_ is somewhat constrained by field measurements. The value of *n*, however, is currently uncertain. Yet, we showed that for *n*>1.001, dependency of the solution on *n* becomes very weak.

We also showed that neglecting dynamic (inertia) effects can lead to a significant underestimation of the slide size and that the volumes of catastrophic slides can exceed the anticipated volumes of progressive slides (estimated based on static consideration) by nearly a factor of two. These results may be useful for assessing the slide damage and tsunamigenic (in the case of a submerged slide) potentials. As an example, we considered Gaviota slide offshore Santa Barbara (California) and Humboldt slide on the Northern California continental margin. In particular, it appears conceivable that Humboldt slide is unfinished and may still displace a sediment slab 32 km long, which could generate a considerable tsunami. Finally, we discussed landslides in normally consolidated sediments and sensitive clays.

## Supplementary Material

Electronic Supplementary Material

## Data accessibility

All data used in this paper are published in the cited references.
