# Supplementary material for: Dynamic growth of slip surfaces in catastrophic landslides
Source: Proc Math Phys Eng Sci. 2016 Jan;472(2185):20150758. doi: 10.1098/rspa.2015.0758 (PMC4786049; doi:10.1098/rspa.2015.0758)
Supplement: Electronic Supplementary Material [file rspa20150758supp1.pdf]

Electronic supplementary material

Germanovich LN, Kim S, Puzrin AM. 2016 Dynamic growth of slip surfaces in catastrophic landslides. *Proc. R. Soc. A* 472: 20150758. http://dx.doi.org/10.1098/rspa.2015.0758

Appendix A. Nomenclature

SYMBOL	DEFINITION
	Latin symbols
A	point $(l_0, 0)$ where the discontinuity initiates (figures 3a, 3b)
A	point (l_A, t_A) where the discontinuity reflects from the band tip (figure 3c)
A_1	infinite point if the discontinuity does not intersect the tip curve $x = l(t)$ (figure 3a)
A, A_1, A_2	points of consecutive reflections of the discontinuity from the band tip (figure 3c)
B	intersection of characteristics AC and PQ (figure 3)
B_1, B_2	intersections of characteristics A_1C_1 and A_2C_2 with P_1Q_1 and P_2Q_2, respectively (figure 3c)
c	$(E/\rho_0)^{1/2}$, speed of longitudinal, plane-strain elastic waves
C, C_1, C_2	points of consecutive arrivals of the discontinuity at $x = 0$ (figures 3b, 3c)
C_S	surface water friction coefficient (for submerged slides)
d/dx	$\partial/\partial x + (\partial t/\partial x)\partial/\partial t$, total derivative
D	intersection of characteristics AC and DR (figures 3b, 3c)
D_1, D_2	intersection of characteristics A_1C_1 and A_2C_2 with D_1R_1 and D_2R_2, respectively (figure 3c)
E	$2G/(1 - \nu_u)$, elastic modulus
g	9.80665 m/s^2, standard acceleration due to gravity
G	shear modulus
G, G_1	intersections of characteristics PK and P_1K_1 with P_1Q_1 and P_2Q_2 (or B_2G_1), respectively, at $x = 0$ (figure 3c, where point Q_2 is not shown)
h	thickness of the sliding layer (depth of the shear band)
H, H_1	intersections of characteristics RQ and R_1Q_1 with R_1K and R_2K_1 (or D_2H_1), respectively, at $x = 0$ (figure 3c)
$I(l)$	integral in (3.7)
J	energy release rate
J_0	fracture (surface) energy for a static or quasi-static fracture
J_c	fracture (surface) energy for a dynamically-propagating fracture
K	arbitrary point above the discontinuity line, CA_1 (figure 3a)
K, K_1	arbitrary points in regions CA_1C_1 and $C_1A_2C_2$ (figures 3b, 3c), respectively
l	shear band length (figure 2)

rspa.royalsocietypublishing.org Proc. R. Soc. A 472: 20150758

l_0	initial shear band length just before it begins propagating dynamically; critical length
l_A	shear band length, which corresponds to point A (figure 3c)
L_{f}	length of static (or progressively propagating) shear band at slope failure
l_{f}	length of dynamic (or catastrophically propagating) shear band at slope failure
l_P	shear band length, which corresponds to point P (figure 3)
l_R	shear band length, which corresponds to point R (figure 3)
l_{s}	shear band length when it stops propagating
M	intersection of characteristics CA_1 and PK (figure 3)
n	$\gamma_0^2/\gamma_{\mathrm{c}}^2$, J_0/J_{c}, 'bluntness' parameter
p	thickness-averaged pore pressure in the sliding layer (above the shear band)
p	thickness-averaged pore pressure prior to shear band appearance
p_0	thickness-averaged, effective, longitudinal stress in the intact layer in the infinite slope
p_{a}	active, thickness-averaged failure stress at the upper end, $x = 0$, of the downhill-sliding layer (figure 2)
p_{h}	hydrostatic, thickness-averaged pressure at the lower end, $x = 0$, of the uphill-sliding layer (figure 8)
p_{p}	longitudinal, thickness-averaged stress at slope failure (passive failure stress) near the band tip, $x = l_{\mathrm{f}}$, for the downhill-sliding layer (figure 2)
P	pore pressure in the sliding layer (above the shear band) or in the infinite slope
P, P_1	points on the band tip line, $x = l(t)$; intersections of characteristics BQ and B_1Q_1 with KP and K_1P_1, respectively (figures 3b and 3c)
Q, Q_1	arbitrary points in regions $ACA_1$and $A_1C_1A_2$ (figure 3), respectively
R, R_1	points on the band tip line, $x = l(t)$; intersections of characteristics DR and D_1R_1 with QH and Q_1H_1, respectively (figures 3b, 3c)
t	time
$t(l)$	time of arrival (equation (3.6)) of the shear band tip at point $x = l$ during the first step of the recurrence process (line AA_1; figures 3a, 3b)
$t_1(l)$	time of arrival (equations (3.17) or (B.6)) of the shear band tip at point $x = l$ during the second step or during the general step of the recurrence process (lines AA_1 or A_1A_2, figure 3c)
t_{f}	time of the slope failure; time of the shear band arrival at $x = l_{\mathrm{f}}$ where failure takes place
t_B	time when the discontinuity arrives at $x = x_B$ (point B in figure 3)
T	τ_*/E, normalized longitudinal load in the slope
T_{w}	τ_{w}/E, dimensionless shear stress caused by the water resistance on the sediment-water interface (figure 2)
T_0	$\bar{T}(l_0)$, average value of $T(x)$ along the initial band length, l_0
T_0	normalized, homogeneous longitudinal load in the slope
$\bar{T}(l)$	average value of $T(x)$ along the shear band of length, l
u	thickness-averaged displacement along the slope
u_{s}	thickness-averaged, static displacement along the slope

u_{x}	displacement along the slope
v	dl/dt, velocity of the shear band tip (figure 2)
V	v/c, dimensionless band tip velocity
v_0	initial velocity of the shear band (at $t = +0$)
x	coordinate along the slope (figure 2).
x_B	physical location of point B on plane (x, t) along the shear band
y	coordinate perpendicular to the slope (figure 2)
	Greek symbols
α	slope angle with respect to the horizontal (figure 2)
β	normalized surface water friction coefficient (for submerged slides, (§8b)
β	slope shape function (§8c)
γ	$\partial u/\partial x$, thickness-averaged, longitudinal (along the slope) strain in the layer above the shear band
$\gamma^{\pm}$	limits of γ when a discontinuity line on the (x, t) plane is approached from different sides (figure 3)
γ_0	$[2J_0/(hE)]^{1/2}$, strain level required at the tip of a static band to start propagation
γ_{a}	$(p_0 - p_{\mathrm{a}})/E$, thickness-averaged strain at $x = 0$
γ_{c}	$[2J_{\mathrm{c}}/(hE)]^{1/2}$, minimal strain level at the tip of the growing, dynamic shear band
γ_{p}	$(p_{\mathrm{p}} - p_0)/E$, critical strain (of passive loading) at the band tip, $x = l_{\mathrm{f}}$, at slope failure
γ_{s}	thickness-averaged, static strain in the layer above the shear band
γ_{t}	tip strain (thickness-averaged strain at the tip of the shear band)
η_{t}	tip slip rate (thickness-averaged slip rate at the tip of the shear band)
Γ	$-\gamma/\gamma_0$, normalized strain
Γ_{s}	$-\gamma_{\mathrm{s}}/\gamma_0$, normalized static strain
Γ_{t}	$-\gamma_{\mathrm{t}}/\gamma_0$, normalized tip strain
$\Delta\Gamma_{\mathrm{t}}$	$-\Delta\gamma_{\mathrm{t}}/\gamma_0$, normalized change of the tip strain when the band tip is overtaken by the discontinuity
δt	limit of Δt when $x \to \infty$
$\delta\tau$	limit of $(c/l_0)\Delta t$ when $x \to \infty$
$\delta^-(B)$	auxiliary strain function in (3.19)
$\bar{\delta}$	Characteristic, averaged (along the tip zone) displacement (slip) in the shear band tip zone
δ_{s}	auxiliary function in (3.4)
Δ_0	discontinuity, $\Delta\gamma = \Delta\eta/c$, on lines AC and CA_1 (figure 3)
Δ_1	discontinuity, $\Delta\gamma = \Delta\eta/c$, on lines A_1C_1 and C_1A_2 (figure 3)
$\Delta\gamma_{\mathrm{t}}$	$\gamma_{\mathrm{t}}(l_A + 0) - \gamma_{\mathrm{t}}(l_A - 0)$, change of γ_{t} when the band tip is overtaken by the discontinuity at point A (figure 3c) or the discontinuity originates at point A (figures 3a, 3b)
$\Delta\gamma$	$\gamma^+ - \gamma^-$, discontinuity in γ
$\Delta\eta_{\mathrm{t}}$	$\eta_{\mathrm{t}}(l_A + 0) - \eta_{\mathrm{t}}(l_A - 0)$, change of η_{t} when the band tip is overtaken by the discontinuity at point A (figure 3c) or the discontinuity originates at point A (figures 3a, 3b)
$\Delta\eta$	$\eta^+ - \eta^-$, discontinuity in η

rspa.royalsocietypublishing.org Proc. R. Soc. A 472: 20150758

ΔP	excess pore pressure on the rupture (slip) surface
Δt	difference between the arrival times of the discontinuity and the band tip at the same location (figure 3a)
$\Delta\Omega_{\mathrm{t}}$	$\Delta\eta_{\mathrm{t}}/(c\gamma_0)$, normalized change of the tip slip rate when the band tip is overtaken by the discontinuity
η	$\partial u/\partial t$, slip rate
$\eta^{\pm}$	limits of η when a discontinuity line on the (x,t) plane is approached from different sides (figure 3)
$\bar{\eta}$	slide velocity; slip rate averaged along the band
λ	l/l_0, dimensionless length of the shear band
λ_*	$\gamma_0 h/(T_0 l_0) = \gamma_0/(\gamma_0 + \gamma_{\mathrm{a}})$, dimensionless strain parameter (figure 2, downhill propagation)
λ_*	$\gamma_0 h/(T_0 l_0) = \gamma_0/(\gamma_0 - \gamma_{\mathrm{h}})$, dimensionless strain parameter in the dynamic version of the Palmer and Rice model [1] (figure 8, uphill propagation)
μ	coefficient of residual friction
ν	drained Poisson's ratio (0.25 – 0.35 for many soils and sediments)
ν_{u}	undrained Poisson's ratio (≈0.5 for many saturated soils and sediments)
ξ	x/l_0, dimensionless coordinate along the slope
ρ_0	total material density (accounts for both solid matrix and pore fluid)
ρ_{w}	density of water (equal in the pore space and water column)
σ	x-component (along the slope) of the total, thickness-averaged stress in the sliding layer
σ'_y	effective stress normal to the slope
σ'_{v}	$-\sigma'_y$, negative of the effective stress normal to the slope
σ_{xx}	x-component (along the slope) of the total stress
τ	ct/l_0, dimensionless time
τ_{b}	$\rho_{\mathrm{w}} g \sin\alpha$, along-the-slope component of the buoyancy load
τ_{d}	dynamic traction caused by the residual friction on the slip surface (figure 9a).
τ_{g}	gravitational component of the shear traction on the slip surface (figure 2)
τ_{r}	residual shear stress (figure 2)
τ_{s}	static traction caused by the residual friction on the slip surface (figure 9a).
τ_{w}	shear stress caused by the water resistance on the sediment-water interface (figure 2)
τ_{xy}	shear resistance on the rupture plane or shear band sides
τ_*	$\tau_{\mathrm{g}} - \tau_{\mathrm{b}} - \tau_{\mathrm{r}} - \tau_{\mathrm{w}}$, combined distributed gravitational, frictional, buoyant, and viscous loads in the slope direction (net driving stress)
ω	size of the process zone at the shear band tip (figure 2)
Ω	$\eta/(c\gamma_0)$, dimensionless slip rate
$\bar{\Omega}$	$\bar{\eta}/(c\gamma_0)$, dimensionless slide velocity; dimensionless slip rate averaged along the slop
Ω_{t}	$\eta_{\mathrm{t}}/(c\gamma_0)$, dimensionless slip rate at the band tip

Appendix B. Recurrence and asymptotic solutions

(a) Recurrence solution

In this work, the shear band (rupture) growth prior to its dynamic propagation is considered sufficiently slow for the inertia effects to be negligible yet sufficiently fast for the overlying sediments to deform in the undrained manner. Equation (2.2) holds because the sediment deformation during the dynamic phase of the band growth is considered to be undrained as well. This equation also holds in the case of dry sediments or drained deformation (before and after the shear band appears) if the drained Poisson ratio, ν, is used instead of the undrained Poisson ratio , ν_{u}. The corresponding initial-boundary value problem is given by equations (2.12) – (2.17).

In another limiting case, not only are the inertia effects negligible before the onset of the dynamic growth, but the deformation of the sediments overlying the shear band is fully drained. In other words, the progressive and catastrophic stages of the landslide development are accompanied, respectively, by the fully drained and undrained deformations of the overlying sediment layer. A separate publication will be devoted to this case. In short, equation (2.2) needs to be modified, but the momentum balance condition (2.3) is still valid if T in the right hand side of (2.3) is modified by redefining τ_* in (2.4). Specifically, τ_* remains the same as in (2.4) for $l_0 < x \leq l$ but becomes $\tau_* = n_*(\tau_{\mathrm{g}} - \tau_{\mathrm{b}} - \tau_{\mathrm{r}}) - \tau_{\mathrm{w}}$ for $0 \leq x < l_0$, where $n_* = (1-\nu)/(1-\nu_{\mathrm{u}})$. Factor n_* represents the effective increase of the longitudinal loading due to the constrains associated with the loading rate (undrained versus drained). The solution obtained in this work is still applicable although the values of the controlling parameters, n and λ_*, will change. Notably, in this case, the condition of different fracture (surface) energies, $J_0 > J_c$, during the quasi-static and dynamic phases of the band growth is not required. The dynamic growth can be triggered even if $J_0 = J_{\mathrm{c}}$, which, owing to the difference in elastic moduli during these phases of the band growth, corresponds to $n = \gamma_0^2/\gamma_c^2 = n_* > 1$. For most soils and soft sediments, $\nu \approx 0.25 - 0.35$ and $\nu_{\mathrm{u}} \approx 0.5$, so that, $n \approx 1.3 - 1.5$. In the examples considered in §7, we used the value of $n = 1.5$.

This appendix summarizes the recurrence solution obtained in §3 and presents additional details. As in §3, point $A(l_A, t_A)$ can be viewed as the initial position of the band tip when $l_A = l_0$, $t_A = 0$ (figures 3a, 3b). It can also be viewed as one of the subsequent tip positions where the discontinuity reflects from the tip, $x = l(t)$, at $l_A > l_0$, $t_A > 0$ (figure 3c). Hence, we consider the general step of the recurrence process (region ACC_1A_1 in figure 3c where $l_A \geq l_0$, $t_A \geq 0$). Unless so stated, we do not use the initial values (3.2) and keep the general notation, $A(l_A, t_A)$.

Assuming τ_{w} independent of t or negligible (i.e., $T(x,t) = T(x)$), the band growth velocity (equation (3.17))

$$\frac{v}{c} \equiv \frac{1}{c}\frac{dl_P}{dt} = \frac{[\gamma_{\mathrm{s}}(l_P) + \delta^-(B)]^2 - \gamma_c^2}{[\gamma_{\mathrm{s}}(l_P) + \delta^-(B)]^2 + \gamma_c^2} \quad (l_A \leq l_P \leq l_{A_1}) \tag{B.1}$$

and strain and the slip rate (equations (3.20))

$$\gamma_{\mathrm{t}}(P) = \frac{\gamma_c^2 + [\gamma_{\mathrm{s}}(l_P) + \delta^-(B)]^2}{2[\gamma_{\mathrm{s}}(l_P) + \delta^-(B)]}, \quad \frac{\eta_{\mathrm{t}}(P)}{c} = \frac{\gamma_c^2 - [\gamma_{\mathrm{s}}(l_P) + \delta^-(B)]^2}{2[\gamma_{\mathrm{s}}(l_P) + \delta^-(B)]} \tag{B.2}$$

at the band tip, $P(l_P, t_P)$, which is the arbitrary point on line AA_1 of the tip positions, $x = l(t)$ (figure 3c). Hereafter, function values at the interval ends are understood as their one-sided limits.

Function $\delta^-(B)$ is defined by (equation (3.19))

$$\delta^-(B) \equiv \delta^-(x_B, t_B) = -\gamma_{\mathrm{s}}(x_B) + \gamma^-(B) - \eta^-(B)/c \tag{B.3}$$

while points B and P in (B.1) and (B.2) are related by (equations (3.18))

$$x_B(l_P, t_P) = \frac{l_A + l_P - c(t_P - t_A)}{2}, \quad t_B(l_P, t_P) = t_A + \frac{l_A - x_B(l_P, t_P)}{c} \tag{B.4}$$

where $0 \leq x_B \leq l_A$ and $t_A \leq t_B \leq t_A + l_A/c$.

Equation (B.1) implies that relation $t_P(l_P)$ between the tip quantities t_P and l_P is given by the solution of the initial value problem

$$\begin{cases} \dfrac{dt_P}{dl_P} = \dfrac{1}{c}\dfrac{[\gamma_s(l_P) + \delta^-(B)]^2 + \gamma_c^2}{[\gamma_s(l_P) + \delta^-(B)]^2 - \gamma_c^2} \quad (l_A \le l_P \le l_{A_1}) \\ t_P = t_A \quad (l_P = l_A) \end{cases} \tag{B.5}$$

where $\delta^-(B) = \delta^-(x_B(l_P, t_P), t_B(l_P, t_P))$ and functions $\gamma^-(B)$ and $\eta^-(B)$ in (B.3) are known either from the initial conditions (3.2) or the solution at the previous recurrence step.

The unique function that satisfies (B.5) is further denoted as

$$t_P = t_1(l_P) \tag{B.6}$$

Once it is found (by solving (B.5)), (B.4) can be rewritten as

$$x_B(l_P) = \frac{l_A + l_P - c(t_1(l_P) - t_A)}{2}, \quad t_B(l_P) = t_A + \frac{l_A - x_B(l_P)}{c} \tag{B.7}$$

so that per (B.3),

$$\delta^-(B(l_P)) = \delta^-(x_B(l_P), t_B(l_P)) \tag{B.8}$$

Equations (B.1) and (B.2) then result in dependencies

$$\frac{v(l_P)}{c} = \frac{[\gamma_s(l_P) + \delta^-(B(l_P))]^2 - \gamma_c^2}{[\gamma_s(l_P) + \delta^-(B(l_P))]^2 + \gamma_c^2} \tag{B.9}$$

and

$$\gamma_t(l_P) = \frac{\gamma_c^2 + [\gamma_s(l_P) + \delta^-(B(l_P))]^2}{2[\gamma_s(l_P) + \delta^-(B(l_P))]}, \quad \frac{\eta_t(l_P)}{c} = \frac{\gamma_c^2 - [\gamma_s(l_P) + \delta^-(B(l_P))]^2}{2[\gamma_s(l_P) + \delta^-(B(l_P))]} \tag{B.10}$$

of the band velocity, tip strain, and tip slip rate on the band length, l_P, for $l_A \le l_P < l_{A_1}$.

Point $A_1(l_{A_1}, t_{A_1})$ where the discontinuity arrives at the band tip is defined by (equation (3.24))

$$c(t_{A_1} - t_A) = l_{A_1} + l_A, \quad t_{A_1} = t_1(l_{A_1}) \tag{B.11}$$

The band length l_P at time t_P is given by the inverse of (B.6).

The strain, γ, and slip rate, η, at the arbitrary point, $Q(l_Q, t_Q)$, in domain ACA_1 (figure 3c) are given by (equations (3.21))

$$\gamma(Q) = \frac{\eta(Q)}{c} + \delta^-(B) + \gamma_s(x_Q), \quad \frac{\eta(Q)}{c} = \frac{\eta_t(l_R)}{c} + \frac{\delta^-(D) - \delta^-(B)}{2} \tag{B.12}$$

where $\eta_t(l_R)$ is defined in (B.10) with l_P replaced by l_R. Because RQ $(t - t_R = (l_R - x)/c)$, AB $(t - t_A = (l_A - x)/c)$, BQ $(t - t_B = (x - x_B)/c)$, and DR $(t - t_R = (x - l_R)/c)$ are characteristic lines with R being at the band tip (hence, (B.6) applies), points Q and B in (3.21) are related by

$$\begin{cases} x_B(x_Q, t_Q) = \dfrac{l_A + x_Q - c(t_Q - t_A)}{2}, \quad t_B(x_Q, t_Q) = t_A + \dfrac{l_A - x_B(x_Q, t_Q)}{c} \\ x_D(l_R, t_R) = \dfrac{l_A + l_R - c(t_R - t_A)}{2}, \quad t_D(l_R, t_R) = t_A + \dfrac{l_A - x_D(l_R, t_R)}{c} \\ l_R(x_Q, t_Q) = x_Q - c(t_R - t_Q), \quad t_R = t_1(l_R) \end{cases} \tag{B.13}$$

For given x_Q and t_Q, $l_R(x_Q, t_Q)$ and $t_R(x_Q, t_Q)$ are defined by the last two equations in (B.13). These functions are then substituted in the previous two equations in (B.13) to find $x_D(x_Q, t_Q)$ and $t_D(x_Q, t_Q)$. Finally, $x_B(x_Q, t_Q)$ and $t_B(x_Q, t_Q)$ are obtained from the first two equations in (B.13) (or (3.22)). Note that the second equation in (B.12) implies that inside ACA_1, the slip rate $\eta(x, t)$ is constant on characteristics $dt/dx = -1/c$ since l_R does not

change as Q moves along these characteristics (figure 3). It is not constant, however, on $dt/dx = 1/c$ because R does move with Q in this case.

Strain and slip rate at the arbitrary point, $K(l_K, t_K)$, in domain CA_1C_1 (figure 3c) are given by (equation (3.23))

$$\begin{cases} \gamma(x_K, t_K) = \dfrac{\eta_t(l_P) - \eta_t(l_R)}{c} + \dfrac{\delta^-(B) - \delta^-(D)}{2} + \gamma_s(x_K) \\ \dfrac{\eta(x_K, t_K)}{c} = \dfrac{\eta_t(l_P) + \eta_t(l_R)}{c} + \dfrac{\delta^-(B) + \delta^-(D)}{2} \end{cases} \tag{B.14}$$

where $\eta_t(l_P)$ and $\eta_t(l_R)$ are both defined by (B.10) written for P and R, respectively. Similar to (B.13), points B and D in (B.14) are related to point K (figure 3c) by

$$\begin{cases} x_B(l_P, t_P) = \dfrac{l_A + l_P - c(t_P - t_A)}{2}, \quad t_B(l_P, t_P) = t_A + \dfrac{l_A - x_B(l_P, t_P)}{c} \\ x_D(l_R, t_R) = \dfrac{l_A + l_R - c(t_R - t_A)}{2}, \quad t_D(l_R, t_R) = t_A + \dfrac{l_A - x_D(l_R, t_R)}{c} \\ l_P(x_K, t_K) = x_K - c[t_P(x_K, t_K) - t_K], \quad t_P = t_1(l_P) \\ t_H(x_K, t_K) = t_K - \dfrac{x_K}{c}, \quad l_R(t_H) = c[t_H - t_R(t_H)], \quad t_R = t_1(l_R) \end{cases} \tag{B.15}$$

For given x_K and t_K, the last three equations give the auxiliary unknown $t_H(x_K, t_K)$ and then $l_R(x_K, t_K)$ and $t_R(x_K, t_K)$. The previous two equations define $l_P(x_K, t_K)$ and $t_P(x_K, t_K)$. Once these quantities are found, the remaining equations in (B.15) result in t_B, x_B, t_D, and x_D as functions of x_K and t_K.

The recurrence process is closed by noting that $\delta^-(B_1)$ and $\delta^-(D_1)$ are the limits of $\delta(K)$ as $K \to B_1$ and $K \to D_1$, respectively (figure 3c). Specifically, $K \to D_1$ corresponds to the limits of $l_P \to l_{A_1}$ and $x_B \to 0$. Hence, for the arbitrary points $D_1(x_{D_1}, t_{D_1})$ and $B_1(x_{B_1}, t_{B_1})$ on the discontinuity line A_1C_1 (figure 3c), we find from (B.14) that

$$\begin{cases} \gamma^-(D_1) = \dfrac{\eta_t(l_{A_1}) - \eta_t(l_R)}{c} + \dfrac{\delta^-(B) - \delta^-(D)}{2} + \gamma_s(x_{D_1}) \\ \dfrac{\eta^-(D_1)}{c} = \dfrac{\eta_t(l_{A_1}) + \eta_t(l_R)}{c} + \dfrac{\delta^-(B) + \delta^-(D)}{2} \end{cases} \tag{B.16}$$

Taking into account that according to (B.3),

$$\delta^-(D_1) = -\gamma_s(x_{D_1}) + \gamma^-(D_1) - \frac{\eta^-(D_1)}{c} \tag{B.17}$$

and using (B.16), we obtain

$$\delta^-(D_1) = -\delta^-(D) - \frac{2\eta_t(l_R)}{c} \tag{B.18}$$

Function

$$\delta^-(B_1) = -\delta^-(B) - \frac{2\eta_t(l_P)}{c} \tag{B.19}$$

is obtained similarly. Expressions (B.18) and (B.19) are combined in (3.25) in the main text.

In (B.18), points D, R, and D_1 are connected by the characteristic lines DR, RH, and HD_1, which can be expressed as

$$\begin{cases} x_{D_1} = l_{A_1} - c(t_{D_1} - t_{A_1}) \\ t_H(x_{D_1}, t_{D_1}) = t_{D_1} - \dfrac{x_{D_1}}{c}, \quad l_R(t_H) = c[t_H - t_R(t_H)], \quad t_R = t_1(l_R) \\ x_D(l_R, t_R) = \dfrac{l_A + l_R - c(t_R - t_A)}{2}, \quad t_D(l_R, t_R) = t_A + \dfrac{l_A - x_D(l_R, t_R)}{c} \end{cases} \tag{B.20}$$

with l_{A_1} and t_{A_1} defined by (B.11). The first equation in (B.20) specifies the relation between x_{D_1} and t_{D_1}, the next three equations give $t_H(x_{D_1}, t_{D_1})$, $l_R(x_{D_1}, t_{D_1})$, and $t_R(x_{D_1}, t_{D_1})$, and the last two equations yield $x_D(x_{D_1}, t_{D_1})$ and $t_D(x_{D_1}, t_{D_1})$ in (B.18). Expressions (B.20) are also valid for $\delta^-(B_1)$ in (B.19) if D_1, H, R, and D are replaced with B_1, G, P, and B respectively (figure 3c). Hence,

$$\begin{cases} x_{B_1} = l_{A_1} - c(t_{B_1} - t_{A_1}) \\ t_G(x_{B_1}, t_{D_1}) = t_{B_1} - \dfrac{x_{B_1}}{c}, \quad l_P(t_G) = c[t_G - t_P(t_G)], \quad t_P = t_1(l_P) \\ x_B(l_P, t_P) = \dfrac{l_A + l_P - c(t_P - t_A)}{2}, \quad t_B(l_P, t_P) = t_A + \dfrac{l_A - x_B(l_P, t_P)}{c} \end{cases} \tag{B.21}$$

Equations (B.18) and (B.19) can now be utilized as the ‘initial’ conditions to find the solution in $A_1C_1C_2A_2$ (figure 3c) directly from (B.6) – (B.15) and (B.18) – (B.21) simply by renaming the unknowns. When $l_A = l_0$, $t_A = 0$ (figures 3a, 3b), (B.3) and (3.2) result in

$$\delta^-(B) = \delta^-(D) = 0 \tag{B.22}$$

which coincides with (3.27) and sets up the recurrence solution discussed above and in §3.

It should be noted that elastic waves in the thin layer above the shear band are nearly one-dimensional. It is the plain-strain, longitudinal wave velocity, $c = (E/\rho_0)^{1/2}$, that controls the energy flux along the sliding layer towards the band tip. Only near the tip, more complex 2-D and 3-D mechanisms take place, but for a thin layer, their contribution is small and asymptotically negligible. Hence, c is the only relevant wave parameter, and the band growth velocity is controlled by c rather than by transverse (shear or Raleigh) waves, which are not present in the 1-D layer under consideration. The band remains subsonic ($v < c$) at all times.

(b) First step in the recurrence solution

At the first step, A is the initial point where the band starts propagating and the discontinuity initiates (i.e., $t_A = 0$, $l_A = l_0$ as in figures 3a, 3b, and B.1). To find the solution in ACC_1A_1 (figure B.1), we substitute (B.22) into (B.1) and (B.2) (or (B.9) and (B.10)). We have

$$\frac{v(l_P)}{c} = \frac{\gamma_s^2(l_P) - \gamma_c^2}{\gamma_s^2(l_P) + \gamma_c^2} \quad (l_0 \le l_P \le l_{A_1}) \tag{B.23}$$

and

$$\gamma_t(l_P) = \frac{\gamma_s^2(l_P) + \gamma_c^2}{2\gamma_s(l_P)}, \quad \frac{\eta_t(l_P)}{c} = -\frac{\gamma_s^2(l_P) - \gamma_c^2}{2\gamma_s(l_P)} \tag{B.24}$$

which are identical with (3.5) and (3.8), respectively. Applying (B.22), equations (B.5) and (B.6) yield

$$t(l) = \int_{l_0}^{l} \frac{dl}{v(l)} = \frac{l + l_0}{c} - \Delta t(l), \quad \Delta t(x) = \frac{2}{c}\left[l_0 - \gamma_c^2 \int_{l_0}^{x} \frac{dl}{\gamma_s^2(l) - \gamma_c^2}\right] \tag{B.25}$$

while (B.12) and (B.14) become

$$\gamma(x_Q, t_Q) = \frac{\eta_t(l_R)}{c} + \gamma_s(x_Q), \quad \eta(x_Q, t_Q) = \eta_t(l_R) \tag{B.26}$$

and

$$\gamma(x_K, t_K) = \frac{\eta_t(l_P) - \eta_t(l_R)}{c} + \gamma_s(x_K), \quad \eta(x_K, t_K) = \eta_t(l_P) + \eta_t(l_R) \tag{B.27}$$

which coincide with (3.11) and (3.14), respectively. In (B.25), we simplified notations by using l instead of l_P.

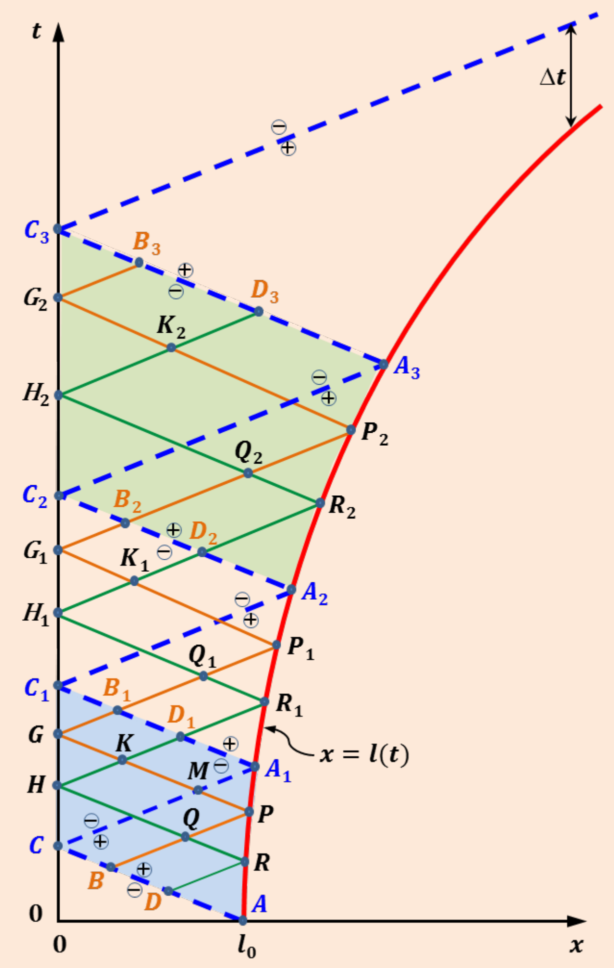

Figure B.1. Location $x = l(t)$ of the shear band tip (bold, solid, curved lines), discontinuity (dashed lines), and some waves (thin, solid, straight lines) traveling between the slide end, $x = 0$, and band tip, $x = l(t)$. Pluses and minuses correspond to the limits of functions $\gamma(x,t)$ and $\eta(x,t)$ when approaching the discontinuity lines from the regions where these functions and their derivatives of the first order are continuous. The first three steps of the recurrence process are shown. The recurrence process truncates if $\Delta t \geq 0$ as $l \to \infty$. This figure is applicable for a shear band propagating both downhill (§§3 and 4) and uphill (§8a).

For a given point Q, point R in (B.26) is defined by (B.13) with $t_A = 0$, $l_A = l_0$ as

$$\begin{cases} x_B(x_Q,t_Q) = \dfrac{l_0 + x_Q - ct_Q}{2}, \quad t_B(x_Q,t_Q) = \dfrac{l_0 - x_B(x_Q,t_Q)}{c} \\ x_D(l_R,t_R) = \dfrac{l_0 + l_R - ct_R}{2}, \quad t_D(l_R,t_R) = \dfrac{l_0 - x_D(l_R,t_R)}{c} \\ l_R(x_Q,t_Q) = x_Q - c\big(t_R - t_Q\big), \quad t_R = t(l_R) \end{cases} \tag{B.28}$$

Similarly, for a given point K, points P and R in (B.27) are defined by (B.15) with $t_A = 0$, $l_A = l_0$ as

$$\begin{cases} x_B(l_P,t_P) = \dfrac{l_0 + l_P - ct_P}{2}, \quad t_B(l_P,t_P) = \dfrac{l_0 - x_B(l_P,t_P)}{c} \\ x_D(l_R,t_R) = \dfrac{l_0 + l_R - ct_R}{2}, \quad t_D(l_R,t_R) = \dfrac{l_0 - x_D(l_R,t_R)}{c} \\ l_P(x_K,t_K) = x_K - c[t_P(x_K,t_K) - t_K], \quad t_P = t(l_P) \\ t_H(x_K,t_K) = t_K - \dfrac{x_K}{c}, \quad l_R(t_H) = c[t_H - t_R(t_H)], \quad t_R = t(l_R) \end{cases} \tag{B.29}$$

Finally, inserting (B.22) into (B.18) and (B.19) results in

$$\delta^-(D_1) = -\frac{2\eta_t(l_R)}{c} = \frac{\gamma_s^2(l_R) - \gamma_c^2}{\gamma_s(l_R)}, \quad \delta^-(B_1) = -\frac{2\eta_t(l_P)}{c} = \frac{\gamma_s^2(l_P) - \gamma_c^2}{\gamma_s(l_P)} \tag{B.30}$$

rspa.royalsocietypublishing.org Proc. R. Soc. A 472: 20150758

which agrees with (3.28). For given points D_1 and B_1, points P and R in (B.30) are given by (B.20) and (B.21) with $t_A = 0$, $l_A = l_0$ as

$$\begin{cases} x_{D_1} = l_{A_1} - c\left(t_{D_1} - t_{A_1}\right) \\ t_H(x_{D_1}, t_{D_1}) = t_{D_1} - \dfrac{x_{D_1}}{c}, \quad l_R(t_H) = c[t_H - t_R(t_H)], \quad t_R = t(l_R) \\ x_D(l_R, t_R) = \dfrac{l_0 + l_R - ct_R}{2}, \quad t_D(l_R, t_R) = \dfrac{l_0 - x_D(l_R, t_R)}{c} \end{cases} \tag{B.31}$$

and

$$\begin{cases} x_{B_1} = l_{A_1} - c\left(t_{B_1} - t_{A_1}\right) \\ t_G(x_{B_1}, t_{B_1}) = t_{B_1} - \dfrac{x_{B_1}}{c}, \quad l_P(t_G) = c[t_G - t_P(t_G)], \quad t_P = t(l_P) \\ x_B(l_P, t_P) = \dfrac{l_0 + l_P - ct_P}{2}, \quad t_B(l_P, t_P) = \dfrac{l_0 - x_B(l_P, t_P)}{c} \end{cases} \tag{B.32}$$

respectively. Note that at the first recurrence step, function $t_1(l)$ is denoted by $t(l)$. Point A_1 is defined by equation (B.11) (or 3.24) with $t_A = 0$, $l_A = l_0$ as

$$ct_{A_1} = l_{A_1} + l_0, \quad t_{A_1} = t\left(l_{A_1}\right) \tag{B.33}$$

which is the same as (3.16).

(c) Second step in the recurrence solution

The second recurrence step (region $A_1C_1C_2A_2$ in figure B.1) is given directly by (B.6) – (B.15) and (B.18) – (B.21), where we only need to rename the variables. We have

$$\frac{v}{c} \equiv \frac{1}{c}\frac{dl_{P_1}}{dt} = \frac{\left[\gamma_s\left(l_{P_1}\right) + \delta^-(B_1)\right]^2 - \gamma_c^2}{\left[\gamma_s\left(l_{P_1}\right) + \delta^-(B_1)\right]^2 + \gamma_c^2} \quad \left(l_{A_1} \le l_{P_1} \le l_{A_2}\right) \tag{B.34}$$

and

$$\gamma_t(P_1) = \frac{\gamma_c^2 + \left[\gamma_s\left(l_{P_1}\right) + \delta^-(B_1)\right]^2}{2\left[\gamma_s\left(l_{P_1}\right) + \delta^-(B_1)\right]}, \quad \frac{\eta_t(P_1)}{c} = \frac{\gamma_c^2 - \left[\gamma_s\left(l_{P_1}\right) + \delta^-(B_1)\right]^2}{2\left[\gamma_s\left(l_{P_1}\right) + \delta^-(B_1)\right]} \tag{B.35}$$

at the band tip, $P_1(l_{P_1}, t_{P_1})$, which is the arbitrary point on the tip position line A_1A_2 (figure B.1). Function $\delta^-(B_1)$ is given in (B.30). Similar to (B.13) or (B.21), points B_1 and P_1 are related by

$$x_{B_1}(l_{P_1}, t_{P_1}) = \frac{l_{A_1} + l_{P_1} - c\left(t_{P_1} - t_{A_1}\right)}{2}, \quad t_{B_1}(l_{P_1}, t_{P_1}) = t_{A_1} + \frac{l_{A_1} - x_{B_1}(l_{P_1}, t_{P_1})}{c} \tag{B.36}$$

where $0 \le x_{B_1} \le l_{A_1}$ and $t_{A_1} \le t_{B_1} \le t_{A_1} + l_{A_1}/c$.

According to (B.5), relation $t_{P_1}(l_{P_1})$ at the band tip is given by the solution of

$$\begin{cases} \dfrac{dt_{P_1}}{dl_{P_1}} = \dfrac{1}{c}\dfrac{\left[\gamma_s\left(l_{P_1}\right) + \delta^-(B_1)\right]^2 + \gamma_c^2}{\left[\gamma_s\left(l_{P_1}\right) + \delta^-(B_1)\right]^2 - \gamma_c^2} \quad \left(l_{A_1} \le l_{P_1} \le l_{A_2}\right) \\ t_{P_1} = t_{A_1} \quad \left(l_{P_1} = l_{A_1}\right) \end{cases} \tag{B.37}$$

Following (B.6), the unique function that satisfies (B.37) is denoted again as

$$t_{P_1} = t_1(l_{P_1}) \tag{B.38}$$

Once it is found (by solving (B.38)), (B.36) defines $x_{B_1}(l_{P_1})$ and $t_{B_1}(l_{P_1})$ while $v(l_{P_1})$, $\gamma_t(l_{P_1})$, and $\eta_t(l_{P_1})$ are given by (B.34) and (B.35). Point $A_2(l_{A_2}, t_{A_2})$, where the discontinuity arrives at the band tip, is defined by modifying (B.11) (or (3.24)) as

$$c(t_{A_2} - t_{A_1}) = l_{A_1} + l_{A_2}, \quad t_{A_2} = t_1(l_{A_2}) \tag{B.39}$$

At the arbitrary point, $Q_1(x_{Q_1}, t_{Q_1})$, in $A_1C_1A_2$ (figure B.1), equations (B.12) yield

$$\gamma(Q_1) = \frac{\eta(Q_1)}{c} + \delta^-(B_1) + \gamma_s(x_{Q_1}), \quad \frac{\eta(Q_1)}{c} = \frac{\eta_t(l_{R_1})}{c} + \frac{\delta^-(D_1) - \delta^-(B_1)}{2} \tag{B.40}$$

where $\eta_t(l_{R_1})$ is defined in (B.35). For a given point, Q_1, point B_1 in (B.40) is defined by (B.13) as

$$\begin{cases} x_{B_1}(x_{Q_1}, t_{Q_1}) = \dfrac{l_{A_1} + x_{Q_1} - c(t_{Q_1} - t_{A_1})}{2}, \quad t_{B_1}(x_{Q_1}, t_{Q_1}) = t_{A_1} + \dfrac{l_{A_1} - x_{B_1}(x_{Q_1}, t_{Q_1})}{c} \\ x_{D_1}(l_{R_1}, t_{R_1}) = \dfrac{l_{A_1} + l_{R_1} - c(t_{R_1} - t_{A_1})}{2}, \quad t_{D_1}(l_{R_1}, t_{R_1}) = t_{A_1} + \dfrac{l_{A_1} - x_{D_1}(l_{R_1}, t_{R_1})}{c} \\ l_R(x_{Q_1}, t_{Q_1}) = x_{Q_1} - c(t_{R_1} - t_{Q_1}), \quad t_{R_1} = t_1(l_{R_1}) \end{cases} \tag{B.41}$$

According to (B.14), for the arbitrary point, $K_1(x_{K_1}, t_{K_1})$, in $C_1A_2C_2$ (figure B.1),

$$\begin{cases} \gamma(x_{K_1}, t_{K_1}) = \dfrac{\eta_t(l_{P_1}) - \eta_t(l_{R_1})}{c} + \dfrac{\delta^-(B_1) - \delta^-(D_1)}{2} + \gamma_s(x_{K_1}) \\ \dfrac{\eta(x_{K_1}, t_{K_1})}{c} = \dfrac{\eta_t(l_{P_1}) + \eta_t(l_{R_1})}{c} + \dfrac{\delta^-(B_1) + \delta^-(D_1)}{2} \end{cases} \tag{B.42}$$

where $\eta_t(l_{P_1})$ and $\eta_t(l_{R_1})$ are given by (B.35). For a given point K_1 (figure B.1a), points B_1 and D_1 in (B.42) are defined by rewriting (B.15) as

$$\begin{cases} x_{B_1}(l_{P_1}, t_{P_1}) = \dfrac{l_{A_1} + l_{P_1} - c(t_{P_1} - t_{A_1})}{2}, \quad t_B(l_{P_1}, t_{P_1}) = t_{A_1} + \dfrac{l_{A_1} - x_{B_1}(l_{P_1}, t_{P_1})}{c} \\ x_{D_1}(l_{R_1}, t_{R_1}) = \dfrac{l_{A_1} + l_{R_1} - c(t_{R_1} - t_{A_1})}{2}, \quad t_{D_1}(l_{R_1}, t_{R_1}) = t_{A_1} + \dfrac{l_{A_1} - x_{D_1}(l_{R_1}, t_{R_1})}{c} \\ l_{P_1}(x_{K_1}, t_{K_1}) = x_{K_1} - c[t_P(x_{K_1}, t_{K_1}) - t_{K_1}], \quad t_{P_1} = t_1(l_{P_1}) \\ t_{H_1}(x_{K_1}, t_{K_1}) = t_{K_1} - \dfrac{x_{K_1}}{c}, \quad l_{R_1}(t_{H_1}) = c[t_{H_1} - t_{R_1}(t_{H_1})], \quad t_R = t_1(l_{R_1}) \end{cases} \tag{B.43}$$

In conclusion, following (B.18) and (B.19) (or (3.25)),

$$\delta^-(D_2) = -\delta^-(D_1) - \frac{2\eta_t(l_{R_1})}{c}, \quad \delta^-(B_2) = -\delta^-(B_1) - \frac{2\eta_t(l_{P_1})}{c} \tag{B.44}$$

where points D_1, R_1, and D_2 are connected by the characteristic lines D_1R_1, R_1H_1, and H_1D_2 (figure B.1). Hence, (B.20) can be expressed as

$$\begin{cases} x_{D_2} = l_{A_2} - c(t_{D_2} - t_{A_2}) \\ t_{H_1}(x_{D_2}, t_{D_2}) = t_{D_2} - \dfrac{x_{D_1}}{c}, \quad l_{R_1}(t_{H_1}) = c[t_{H_1} - t_R(t_{H_1})], \quad t_{R_1} = t_1(l_{R_1}) \\ x_{D_1}(l_{R_1}, t_{R_1}) = \dfrac{l_{A_1} + l_{R_1} - c(t_{R_1} - t_A)}{2}, \quad t_{D_1}(l_{R_1}, t_{R_1}) = t_A + \dfrac{l_{A_1} - x_{D_1}(l_{R_1}, t_{R_1})}{c} \end{cases} \tag{B.45}$$

with l_{A_1}, t_{A_1} and l_{A_2}, t_{A_2} defined by (B.31) and (B.39), respectively. Similarly, relation between points B_1, P_1, and B_2 is given by (B.21) written as

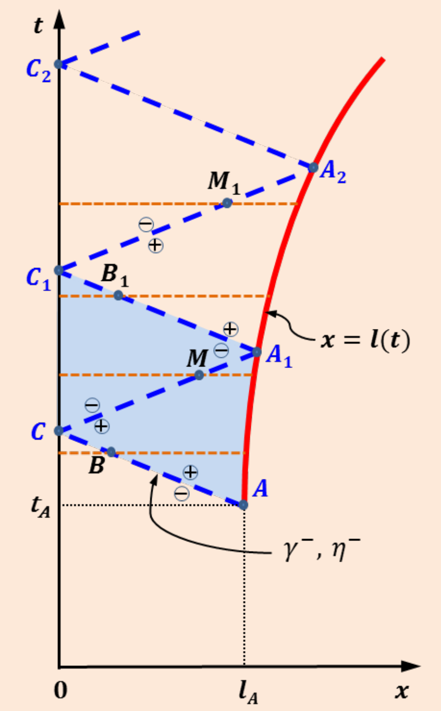

Figure B.2. Positions of the shear band and the layer overlying the shear band (horizontal lines) in the physical space. Points B, M, B_1, and M_1 are locations of the discontinuity. The discontinuity, initiates (if $l_A = l_0$, $t_A = 0$) or reflects (if $l_A > l_0$, $t_A > 0$) at point A and consequently reflects at points C, A_1, C_1, A_2, C_2, and so on.

$$\begin{cases} x_{B_2} = l_{A_2} - c(t_{B_2} - t_{A_2}) \\ t_{G_1}(x_{B_2}, t_{B_2}) = t_{B_2} - \dfrac{x_{B_1}}{c}, \quad l_{P_1}(t_{G_1}) = c[t_{G_1} - t_{P_1}(t_{H_1})], \quad t_{R_1} = t_1(l_{R_1}) \\ x_{B_1}(l_{P_1}, t_{P_1}) = \dfrac{l_{A_1} + l_{P_1} - c(t_{P_1} - t_A)}{2}, \quad t_{B_1}(l_{P_1}, t_{P_1}) = t_A + \dfrac{l_{A_1} - x_{B_1}(l_{P_1}, t_{P_1})}{c} \end{cases} \tag{B.46}$$

(d) Recurrence relations for slide velocity

To find the recurrence relations for the slide velocity, $\bar{\eta}$ (§6). Consider point $A(l_A, t_A)$ where the discontinuity initiates, when the band begins growing at $t = 0$ (figures 3a, 3b, and B.1), or reflects from the tip at time t_A, when it has the length of l_A (figures 3c and B.2). The objective is to express the slide velocity in domain ACC_1A_1 (figure B.2) through the solution valid below (or on) the discontinuity line AC. Then, the slide velocity in domain $A_1C_1C_2A_2$ (at the next step of the recurrence process) is found by using the solution in ACC_1A_1, which is just below $A_1C_1C_2A_2$ (figures 3c and B.2). Since the initial slide velocity, $\bar{\eta}(l_0) = 0$ at $l_A = l_0$ and $t_A = 0$ is known, this will establish the recurrence sequence for $\bar{\eta}$.

Slide velocity is defined as the average slip rate (6.1). This velocity is evaluated similar to (6.2). We first integrate the second equation in (2.12) over x for constant t from interval $t_A < t < t_A + l_A/c$ (figures 3c and B.2). In other words, we integrate this equation along the horizontal line passing through point B (figure B.2). As a result, we obtain

$$\frac{1}{c^2}\int_0^{x_B(t)} \frac{\partial \eta}{\partial t} dx + \frac{1}{c^2}\int_{x_B(t)}^{l(t)} \frac{\partial \eta}{\partial t} dx = \int_0^{x_B} \frac{\partial \gamma}{\partial x} dx + \int_{x_B}^{l} \frac{\partial \gamma}{\partial x} dx + \frac{1}{h}\int_0^l T(x) dx \tag{B.47}$$

where $x_B(t) = l_A - c(t - t_A)$. Changing the order of integration and differentiation in the left hand side integrals and taking into account that both l and x_B are functions of time, we rewrite (B.47) as

$$\frac{\partial}{\partial t}\int_0^l \eta dx = v(l)\eta_t(l) - c[\eta^+(x_B, t) - \eta^-(x_B, t)] + c^2[\gamma_t(l) - \gamma_s(l) - \gamma^+(x_B, t) + \gamma^-(x_B, t)] \tag{B.48}$$

where we used (2.10), (3.7), and $v(l)$ defined by (3.17). We also used $\gamma(l,t) = \gamma_{\mathrm{t}}(l)$ and $\eta(l.t) = \eta_{\mathrm{t}}(l)$ given by (3.20). Using the jump condition (2.17) and integrating (B.48) with respect to time yields

$$\int_0^l \eta dx = \int_0^{l_A} \eta dx + \int_{l_A}^l \eta_{\mathrm{t}}(l) dl + c^2 \int_{l_A}^l \frac{\gamma_{\mathrm{t}}(l) - \gamma_{\mathrm{s}}(l)}{v(l)} dl \tag{B.49}$$

where we took into account that $dl = vdt$. Substituting (B.49) into (6.1), we obtain

$$\frac{\bar{\eta}(l)}{c} = \frac{l_A}{l}\frac{\bar{\eta}(l_A)}{c} + \frac{1}{l}\int_{l_A}^l \left[\frac{\eta_{\mathrm{t}}(l)}{c} + c\frac{\gamma_{\mathrm{t}}(l) - \gamma_{\mathrm{s}}(l)}{v(l)}\right] dl \quad (l_A < l < l_{A_1}) \tag{B.50}$$

Determining $\bar{\eta}(l)$ for $t_A + l_A/c < t < t_A + (l_A + l_{A_1})/c$ is similar, but with point M (on line CA_1 in figures 3c and B.2) instead of B and l_{A_1} defined by (B.11) (or (3.24)). Taking into account that $x_M(t) = l_A + c(t - t_A)$ and that the discontinuity travels towards $x = 0$ with the speed of c, we obtain

$$\frac{\partial}{\partial t}\int_0^l \eta dx = v(l)\eta_{\mathrm{t}}(l) - c[\eta^+(x_M, t) - \eta^-(x_M, t)] + c^2[\gamma_{\mathrm{t}}(l) - \gamma_{\mathrm{s}}(l) - \gamma^+(x_M, t) + \gamma^-(x_M, t)] \tag{B.51}$$

instead of (B.48). Using the jump condition (2.17) and integrating (B.51) with respect to time, yields

$$\int_0^l \eta dx = \int_0^{l_C} \eta dx + \int_{l_C}^l \eta_{\mathrm{t}}(l) dl + c^2 \int_{l_C}^l \frac{\gamma_{\mathrm{t}}(l) - \gamma_{\mathrm{s}}(l)}{v(l)} dl \tag{B.52}$$

where $l_C = l(t_C)$ is the shear band length when the discontinuity is at point $C(0, t_C)$ at the time, t_C, of the discontinuity reflection from $x = 0$.

Although equation (B.52) is nearly identical to (B.49), the integration interval now involves l_C. The first integral in (B.52) can be expressed from (B.49) as

$$\int_0^{l_C} \eta dx = \int_0^{l_A} \eta dx + \int_{l_A}^{l_C} \eta_{\mathrm{t}}(l) dl + c^2 \int_{l_A}^{l_C} \frac{\gamma_{\mathrm{t}}(l) - \gamma_{\mathrm{s}}(l)}{v(l)} dl \tag{B.53}$$

With (B.53) in (B.52), the average slide velocity for $t_A + l_A/c < t < t_A + (l_A + l_{A_1})/c$ becomes the same as (B.50). Including the ends of the interval (since $\bar{\eta}(l)$ is a continuous function), we finally have

$$\frac{\bar{\eta}(l)}{c} = \frac{l_A}{l}\frac{\bar{\eta}(l_A)}{c} + \frac{1}{l}\int_{l_A}^l \left[\frac{\eta_{\mathrm{t}}(l)}{c} + c\frac{\gamma_{\mathrm{t}}(l) - \gamma_{\mathrm{s}}(l)}{v(l)}\right] dl \quad (l_A \le l \le l_{A_1}) \tag{B.54}$$

Solution (B.54) is valid from time $t = t_A$ through time $t = t_{A_1}$ when the discontinuity reaches the band tip at point A_1. The initial slide velocity,

$$\bar{\eta}(l_0) = 0 \quad (l_A = l_0, \ \ t_A = 0) \tag{B.55}$$

Hence, (B.54) and (B.55) establish the recurrence sequence to find the slide velocity, $\bar{\eta}(l)$, for $t \ge t_A$ since at each "step" (figures 3c and B.2), the first term, $\bar{\eta}(l_A)$, in the right side of (B.54) is found during the previous "step".

Substituting strain and slip rate (3.20) at the tip together with the band tip velocity (3.17), $\delta^-(B) = \delta^-(D) = 0$, and $\bar{\eta}(l_0) = 0$ into (B.54) results in (6.5). Therefore, (6.5) is not only valid for $t < l_0/c$, but also for $l_0/c < t < (l_0 + l_{A_1})/c$ (since (B.54) is applicable for $t_A < t < t_A + (l_A + l_{A_1})/c$).

For the integration interval of $A_1C_1C_2A_2$ above point A_1 but below point A_2 (figure B.2), that is, for $(l_0 + l_{A_1})/c < t < (l_0 + 2l_{A_1} + l_{A_2})/c$, the slide velocity is obtained directly from (B.54) by replacing l_A with l_{A_1}. Including the interval ends yields

$$\frac{\bar{\eta}(l)}{c} = \frac{l_{A_1}}{l}\frac{\bar{\eta}(l_{A_1})}{c} + \frac{1}{l}\int_{l_{A_1}}^l \left[\frac{\eta_{\mathrm{t}}(l)}{c} + c\frac{\gamma_{\mathrm{t}}(l) - \gamma_{\mathrm{s}}(l)}{v(l)}\right] dl \quad (l_{A_1} \le l \le l_{A_2}) \tag{B.56}$$

where $v(l)$, $\gamma_t(l)$, and $\eta_t(l)$ are defined by (B.9) and (B.10) with $\delta^-(B_1)$ and $\delta^-(D_1)$ given in (B.30). In the case of $l_A = l_0$, $t_A = 0$, (B.56) is reduced to (6.6). Equations (B.54) and (B.56) (as well as (6.5) and (6.6)) give the same value of $\bar{\eta}(l_{A_1})$ when $l \to l_{A_1}$, which shows that the slide velocity is a continuous function of t (or l).

It should be noted that the recurrence steps given by (B.54) and (B.56) can be combined in a single expression as follows. Using (B.55), we first write (B.54) for the first recurrence step as

$$\frac{\bar{\eta}(l)}{c} = \frac{1}{l}\int_{l_0}^{l}\left[\frac{\eta_t(l)}{c} + c\frac{\gamma_t(l) - \gamma_s(l)}{v(l)}\right]dl \quad (l_0 = l_A \le l \le l_{A_1}) \tag{B.57}$$

For $l = l_{A_1}$, (B.57) yields $\bar{\eta}(l_{A_1})$, which we substitute in (B.56) to obtain

$$\frac{\bar{\eta}(l)}{c} = \frac{l_{A_1}}{l}\frac{1}{l_{A_1}}\int_{l_0}^{l_{A_1}}\left[\frac{\eta_t(l)}{c} + c\frac{\gamma_t(l) - \gamma_s(l)}{v(l)}\right]dl + \frac{1}{l}\int_{l_{A_1}}^{l}\left[\frac{\eta_t(l)}{c} + c\frac{\gamma_t(l) - \gamma_s(l)}{v(l)}\right]dl \tag{B.58}$$

so that

$$\frac{\bar{\eta}(l)}{c} = \frac{1}{l}\int_{l_0}^{l}\left[\frac{\eta_t(l)}{c} + c\frac{\gamma_t(l) - \gamma_s(l)}{v(l)}\right]dl \quad (l_{A_1} \le l \le l_{A_2}) \tag{B.59}$$

gives the slide velocity at the second recurrence step. Equations (B.57) and (B.59) has the same form and can now be combined as

$$\frac{\bar{\eta}(l)}{c} = \frac{1}{l}\int_{l_0}^{l}\left[\frac{\eta_t(l)}{c} + c\frac{\gamma_t(l) - \gamma_s(l)}{v(l)}\right]dl \quad (l_0 \le l \le l_{A_2}) \tag{B.60}$$

Consideration of the other steps is similar, and we see that, in general, the slide velocity is defined by

$$\frac{\bar{\eta}(l)}{c} = \frac{1}{l}\int_{l_0}^{l}\left[\frac{\eta_t(l)}{c} + c\frac{\gamma_t(l) - \gamma_s(l)}{v(l)}\right]dl \quad (l \ge l_0) \tag{B.61}$$

which, therefore, is valid at the arbitrary recurrence step.

In conclusion, (B.61) can be written in the form of

$$\int_0^l \frac{\eta(x,t)}{c}dx = \int_{l_0}^{l}\left[\frac{\eta_t(l)}{c} + c\frac{\gamma_t(l) - \gamma_s(l)}{v(l)}\right]dl \quad (l \ge l_0) \tag{B.62}$$

Therefore, (6.4) in the main text is valid not only for $l_0 \le l \le l_{A_1}$, but for the arbitrary $l \ge l_0$.

(e) Asymptotic solution

A shear band typically acquires a velocity of propagation comparable to the speed c of elastic waves after it propagates the distance of approximately two or three times its original length (§4). If we are interested in lengths of $l \gtrsim 2l_0$, it is natural to simplify the solution by assuming the band velocity, v, reaches c already at $t = 0$ and remains constant after that. For $v = c$, the band length at time t is

$$l(t) = l_0 + ct \tag{B.63}$$

and the boundary condition (2.6) yields

$$\frac{\eta(l)}{c} + \gamma(l) = 0 \tag{B.64}$$

In this approximation, the discontinuity, after it reflects at $x = 0$, never reaches the band tip (figure B.3) since the band propagates with the same speed as the discontinuity. This approximation is asymptotic for large band sizes

rspa.royalsocietypublishing.org Proc. R. Soc. A 472: 20150758

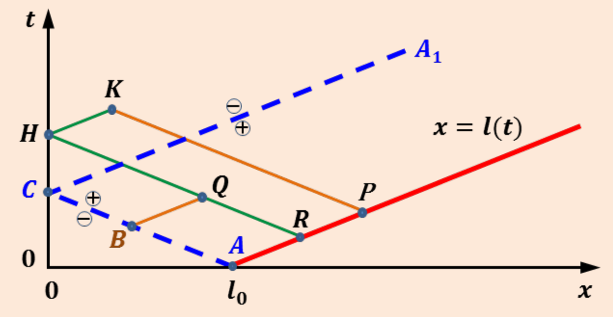

Figure B.3. Shear band propagation with the speed, c, of elastic waves. The tip location line, $x = l(t) = l_0 + ct$, and characteristic line, CA_1, of the propagating discontinuity have the same slopes $dl/dx = 1/c$ and do not intersect. Q and K are arbitrary points below and above CA_1, respectively. They correspond to points Q and K, respectively, in figures 3 and B.1.

because in the exact solution (B.9) (or (3.17)), $v(l)/c \to 1$ as $l \to \infty$ (even if the discontinuity arrives at the band tip one or several times).

It follows from (B.23) (or (3.5)), $v(l)/c \to 1$ also when $\gamma_c \to 0$ or, which is the same for any given n, when $\gamma_0 \to 0$ (equation (2.11)). This implies that the asymptotes of a large l and small γ_0 (or small γ_c) are the same, which suggests that in the absence of the resistance to the fracture growth (since $\gamma_c \to 0$ or $\gamma_0 \to 0$), the band velocity jumps to c (but does not exceed c) immediately at the onset of growth. Similarly, $\gamma_c \to 0$ if $n \to \infty$ for any given γ_0 (equation (2.11)). So the asymptotes of $l \to \infty$ and $n \to \infty$ are equivalent as well. Therefore, even when the resistance to the band propagation due to the friction near in the tip zone is small compared to the resistance due to the residual friction on the band sides (because the band sides are much larger than the tip zone), the effect of the tip zone resistance may be important since it is responsible for the increase of the band velocity in the beginning of its dynamic growth. Ignoring the tip resistance is acceptable if the time scale of band acceleration is much less than the time scale of interest.

Because the asymptotes of $\gamma_c \to 0$ and of $n \gg 1$ (or $l \gg l_0$) are the same, similar to (B.26) (or (3.11)), for the arbitrary point $Q(x_Q, t_Q)$ below the discontinuity line CA_1 ($x = ct - l_0$) in figure B.3,

$$\gamma(x_Q, t_Q) = \gamma_s(x_Q) + \frac{\eta(x_Q, t_Q)}{c}, \quad \frac{\eta(x_Q, t_Q)}{c} = -\frac{\gamma_s(l_R)}{2} \quad (n \gg 1) \tag{B.65}$$

and similar to (B.27) (or (3.14)), for the arbitrary point $K(x_K, t_K)$ above line CA_1 in figure B.3,

$$\gamma(x_K, t_K) = \gamma_s(x_K) + \frac{\gamma_s(l_R) - \gamma_s(l_P)}{2}, \quad \frac{\eta(x_K, t_K)}{c} = -\frac{\gamma_s(l_P) + \gamma_s(l_R)}{2} \quad (n \gg 1) \tag{B.66}$$

According to (B.63), $ct_P = l_P - l_0$, so quantities l_P and l_R are related to $Q(x_Q, t_Q)$ and $K(x_K, t_K)$ by

$$l_P = x_K - c(t_P - t_K) = \frac{ct_K + l_0 + x_K}{2}, \quad l_R = x_Q - c(t_P - t_Q) = \frac{ct_Q + l_0 + x_Q}{2} \tag{B.67}$$

At the band tip, $x = l_f$, the boundary condition (2.6) (for $v = c$) combined with the slope failure condition, $\gamma(l_f, t_f) = -\gamma_p$ (§5), results in the normalized slip rate, $\eta(l_f, t_f)/c = \gamma_p$ at the band tip at failure. With $Q \to R$ (figure B.3) and $l_R = l_f$, (B.65) yields condition

$$\gamma_s(l_f) = -\gamma_p \tag{B.68}$$

for the band length, l_f, at global slope failure.

In the case of a homogeneous load (4.1), the static strain is given by (4.2). Using (3.30), (4.2), and (B.67), the material strain and slip rate are obtained from (B.65) (i.e., below line CA_1 in figure B.3) as

$$\begin{cases} \Gamma = -\dfrac{\gamma(x,t)}{\gamma_0} = \dfrac{1}{2} + \dfrac{1}{4\lambda_*}\left(\dfrac{3x - ct}{l_0} - 3\right) \\ \Omega = \dfrac{\eta(x,t)}{c\gamma_0} = \dfrac{1}{2} + \dfrac{1}{4\lambda_*}\left(\dfrac{ct + x}{l_0} - 1\right) \end{cases} \quad (t \le (l_0 + x)/c, \ n \gg 1) \tag{B.69}$$

and from (B.66) (i.e., above line CA_1 in figure B.3) as

rspa.royalsocietypublishing.org Proc. R. Soc. A 472: 20150758

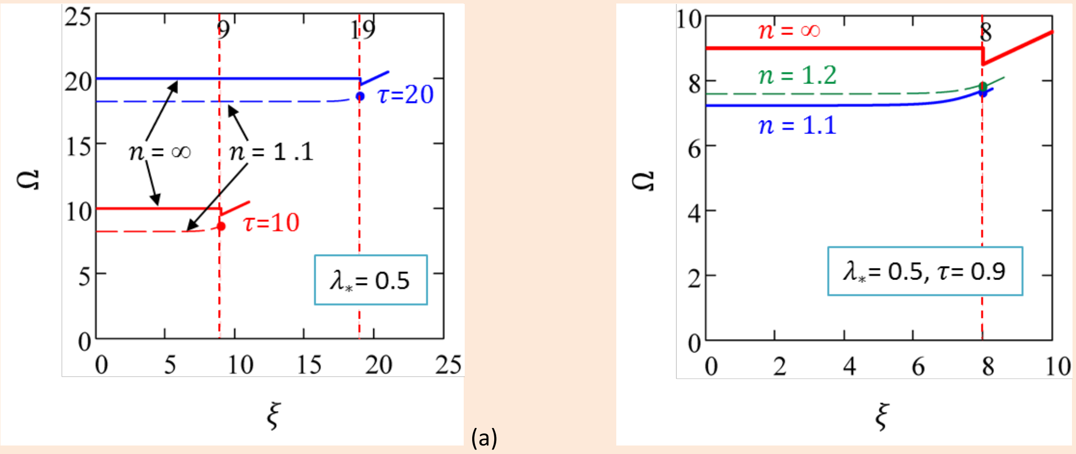

Figure B.4. (a) Dimensionless slip rate, $\Omega = \eta/(c\gamma_0)$, along the slope as a function of dimensionless longitudinal coordinate, $\xi = x/l_0$, at dimensionless times, $\tau = ct/l_0$, of 10 and 20. Solid lines show the asymptotic solution (B.69) and (B.70) (when $v = c$), while dashed lines correspond to the exact solution (where $v = v(l)$) presented in §3 and Appendix Bb. This exact solution is plotted for $T(x) = T_0 = \text{const}$ (§5), $\lambda_* = 0.5$, and $n = 1.1$ (when the discontinuity never reaches the band tip). The magnitude of the discontinuity is $\Delta_0 = 0.5$ in asymptotic (solid line) and 4.545×10^{-2} in the exact (dashed line) solutions. (b) Effect of the value of n on Ω. The exact solution (§3 and Appendix Bb) for $T(x) = T_0 = \text{const}$ (§5) is plotted for $n = 1.1$ (blue, solid line) and $n = 1.2$ (green, dashed line) at $\tau = 9$. In both cases, $\lambda_* = 0.5$. The corresponding magnitudes of discontinuity are $\Delta_0 = 4.545 \times 10^{-2}$ and 8.333×10^{-2}, respectively. The asymptotic solution, given by (B.65) and (B.66) (red, bold line), corresponds to $n \to \infty$. In both figures (a) and (b), dots indicate locations of the discontinuities, which are not visible at the figure scales.

$$\begin{cases} \Gamma = -\dfrac{\gamma(x,t)}{\gamma_0} = 1 + \dfrac{1}{2\lambda_*}\left(\dfrac{x}{l_0} - 2\right) \\ \Omega = \dfrac{\eta(x,t)}{c\gamma_0} = 1 + \dfrac{1}{2\lambda_*}\left(\dfrac{ct}{l_0} - 1\right) \end{cases} \quad (t \geq (l_0 + x)/c, \;\; n \gg 1) \tag{B.70}$$

As expected, for $t = (l_0 + x)/c$, (B.69) and (B.70) give different results, which represents the discontinuity moving along this line (i.e., line CA_1 in figure B.3). The discontinuity magnitude $\Delta\Gamma(M) = \Gamma^+(M) - \Gamma^-(M) = \Delta\Omega(M) = \Omega^+(M) - \Omega^-(M) = -1/2$ is independent of the location of point M on CA_1, which is consistent with the general result presented in Appendix C. Furthermore, in (B.70), η is independent of x, although it is changing with time. Note that a part of the slide (along the slope) where $\eta(x,t)$ is spatially constant becomes larger with time (as the shear bands grows).

Because the discontinuity does not ever reach the band tip for $n \geq 1.4392$ (figure 4a), the limit of $n \to \infty$ (or $l \to \infty$) can also be applied directly to (B.26) and (B.27) (or to (3.11) and (3.14)), which, together with (4.2), results again in (B.69) and (B.70), respectively, if $T(x) = T_0 = \text{const}$. At the moment of slope failure, $l_f = l_0 + ct_f$. Hence, the failure condition $\gamma(l_f, t_f) = -\gamma_p$, used with the first equation in (B.69), yields the shear band length at failure

$$\frac{l_f}{l_0} = 1 + \lambda_*\left(\frac{2\gamma_p}{\gamma_0} - 1\right) \tag{B.71}$$

Given that the expected value of γ_p is at least several times greater than γ_0 (§7) and that $n > 1$, l_f in (B.71) is only slightly larger than in the exact solution (5.3).

Therefore, for $T(x) = T_0 = \text{const}$, the normalized strain, Γ, and slip rate, Ω, are given by (B.69) and (B.70). Distributions of Ω along the slope are shown in figure B.4a (solid lines) for two dimensionless times, $\tau = 10$ and 20, and $\lambda_* = 0.5$. For comparison, the exact solution (§3) is also plotted (dashed lines) in figure B.4a for the same times and λ_*. The exact solution, however, depends upon n, so we used $n = 1.1$ when the discontinuity does not reach the band tip. As can be seen, the patterns of Ω are similar for the exact and asymptotic solutions, although

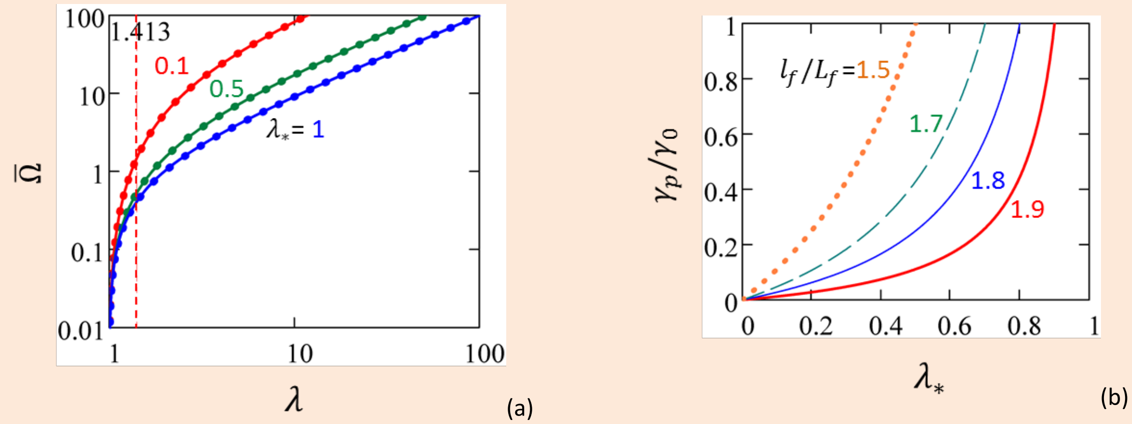

Figure B.5. (a) Dimensionless slide velocity, $\bar{\Omega} = \bar{\eta}/(c\gamma_0)$, as a function of dimensionless band length, $\lambda = l/l_0$, in the case of $T(x) = T_0 = \text{const}$ (§5). Solid lines correspond to the exact solution (6.7) (where $v = v(l)$) with $n = 1.1$, while dotted lines represent the asymptotic solution (B.73) (when $v = c$ and $n = \infty$). Red, green, and blue lines correspond to $\lambda_* = 0.1$, 0.5 and 1, respectively. For $\lambda_* = 0.1$ and 0.5 with $n = 1.1$, the discontinuity does not reach the band tip in the exact solution (solid, red and green lines). For $\lambda_* = 1$ and $n = 1.1$, the blue, solid line is plotted for both solutions in (6.7), that is, before and after the discontinuity visits the tip at $\xi = 1.413$ $(\tau = 2.413)$. (b) Contour lines $l_f/L_f = 1.9$ (red, bold line), 1.8 (blue, thin line), 1.7 (green, dashed line), and 1.5 (orange, dotted line) l_f/L_f in λ_*-γ_p/γ_0 coordinates for $n \to \infty$.

the magnitudes of the discontinuity jump differ by an order (0.5 and 0.045, respectively). These magnitudes do not change with time, but their relative values decrease (figure B.4a). The difference between the asymptotic, (B.71), and exact, (5.3), solutions is insignificant for large enough values of τ (figure B.4a).

Effect of n on the distribution of Ω along the slope can be seen in figure B.4b for $n = 1.1$ (blue, thin line) and 1.2 (green, dashed line) for $\tau = 9$. The physical locations of the discontinuity are the same, but the magnitude, Δ_0, of the discontinuity of Ω increases with n. For example, $\Delta_0 = 4.545 \times 10^{-2}$ and 8.333×10^{-2} for $n = 1.1$ and $n = 1.2$, respectively. Note that at any given time, the band length (solid lines in figure B.4a) in the asymptotic solution is slightly longer than in the exact one (dashed lines in figure B.4a) because the band tip velocity is larger in the asymptotic solution.

The normalized slide velocity, $\bar{\Omega} = \bar{\eta}/(\gamma_0 c)$, can be obtained either by averaging the slip rate in (B.65) and (B.66) at a given time or directly from (6.5). This results in

$$\frac{\bar{\eta}(l)}{c} = -\frac{1}{l}\int_{l_0}^{l} \gamma_s(l)dl \quad (l = l_0 + ct, \;\; t > 0) \tag{B.72}$$

which is valid for any time, since the discontinuity does not reach the band tip if $v = c$. In the case of the homogeneous distribution (4.1), (4.2) and (B.72) yield

$$\bar{\Omega}(\lambda) = \frac{\bar{\eta}(l)}{c\gamma_0} = \frac{\lambda - 1}{\lambda}\left(1 + \frac{\lambda - 1}{2\lambda_*}\right) \tag{B.73}$$

where $\lambda = l/l_0$ and (B.73) is the same as the first equation in (6.7).

Dependence (B.73) for $\bar{\Omega}(\lambda)$ is shown in figure B.5a for $\lambda_* = 0.1$, 0.5, 1 and $n = 1.1$. For $\lambda_* = 0.1$ and 0.5 and $n = 1.1$, the discontinuity does not reach the tip and the exact and asymptotic solutions are nearly identical. But even for $\lambda_* = 0.1$ and $n = 1.1$ (blue solid and dotted lines), when the discontinuity arrives at the tip at $\tau = 2.413$ and $\lambda = 1.413$ (in the exact solution), the asymptotic solution still provides a close match to the exact solution.

The dynamic failure length (B.71) in the asymptotic solution can be written as

$$\frac{l_f}{l_0} = \frac{L_f}{l_0} + \lambda_* \frac{\gamma_p}{\gamma_0} \tag{B.74}$$

where the static failure length, L_{f}, is given by (5.6). Hence, the asymptotic dynamic-to-static failure ratio is

$$\frac{l_{\mathrm{f}}}{L_{\mathrm{f}}} = 1 + \left[1 - \frac{\gamma_0}{\gamma_{\mathrm{p}}}\left(1 - \frac{1}{\lambda_*}\right)\right]^{-1} \tag{B.75}$$

Because $\gamma_0/\gamma_{\mathrm{p}} < 1$ and $0 \le \lambda_* \le 1$, ratio $l_{\mathrm{f}}/L_{\mathrm{f}}$ is always greater than 1, which is similar to the exact solution (§5).

Isoclines of $l_{\mathrm{f}}/L_{\mathrm{f}}$ are shown in figure B.5b. It can be seen that for small values of $\gamma_0/\gamma_{\mathrm{p}}$, $l_{\mathrm{f}}/L_{\mathrm{f}}$ becomes relatively close to 2. For example, $l_{\mathrm{f}}/L_{\mathrm{f}} > 1.8$ if $\gamma_0/\gamma_{\mathrm{p}} < 0.2$ and $\lambda_* > 0.444$ or $\gamma_0/\gamma_{\mathrm{p}} < 0.1$ and $\lambda_* > 0.286$. Such behavior of the asymptotic ratio $l_{\mathrm{f}}/L_{\mathrm{f}}$ is again close to the exact (§5). Indeed, in the exact solution, we would use (5.3) instead of (B.74), which results in

$$\frac{l_{\mathrm{f}}}{L_{\mathrm{f}}} = 1 + \sqrt{1 - \frac{1}{n}\left(\frac{\gamma_0}{\gamma_{\mathrm{p}}}\right)^2}\left[1 - \frac{\gamma_0}{\gamma_{\mathrm{p}}}\left(1 - \frac{1}{\lambda_*}\right)\right]^{-1} \tag{B.76}$$

Although expression (B.76) now includes parameter *n*, it still shows that for small values of $\gamma_0/\gamma_{\mathrm{p}}$, $l_{\mathrm{f}}/L_{\mathrm{f}}$ is close to 2. For example, now $l_{\mathrm{f}}/L_{\mathrm{f}} > 1.8$ if $\gamma_0/\gamma_{\mathrm{p}} < 0.2$ and $\lambda_* > 0.468$ or $\gamma_0/\gamma_{\mathrm{p}} < 0.1$ and $\lambda_* > 0.290$.

Appendix C. Discontinuity

(a) Discontinuity magnitude

At the general step of the recurrence process (region ACC_1A_1 in figures 3c and B.1), consider the arbitrary point B on line AC. For point Q in ACA_1 such that QB is a characteristic line (i.e., $dt/dx = -1/c$), (B.12) (or (3.21)) reads

$$\begin{cases} \gamma(Q) = \dfrac{\eta_{\mathrm{t}}(l_R)}{c} + \dfrac{\delta^-(D) + \delta^-(B)}{2} + \gamma_{\mathrm{s}}(x_Q) \\ \dfrac{\eta(Q)}{c} = \dfrac{\eta_{\mathrm{t}}(l_R)}{c} + \dfrac{\delta^-(D) - \delta^-(B)}{2} \end{cases} \quad (Q \in ACA) \tag{C.1}$$

where we consider the limit of $Q \to B$ and take into account the corresponding limits of $D \to A$ and $R \to A$ (figures 3c and B.1). Then (C.1) yields functions $\gamma^+(B)$ and $\eta^+(B)$, which, in turn, results in the values

$$\begin{cases} \Delta\gamma(B) = \gamma^+(B) - \gamma^-(B) = \dfrac{\eta_{\mathrm{t}}(l_A + 0)}{c} + \dfrac{\delta^-(A) + \delta^-(B)}{2} + \gamma_{\mathrm{s}}(x_B) - \gamma^-(B) \\ \Delta\eta(B) = \eta^+(B) - \eta^-(B) = \eta_{\mathrm{t}}(l_A + 0) - \eta^-(B) + c\,\dfrac{\delta^-(A) - \delta^-(B)}{2} \end{cases} \tag{C.2}$$

of the discontinuity on AC. Using (3.19) in (C.2), we see that

$$\Delta\gamma(B) = \frac{\Delta\eta(B)}{c} = \frac{\eta_{\mathrm{t}}(l_A + 0)}{c} + \frac{\delta^-(A) + \gamma_{\mathrm{s}}(x_B) - \gamma^-(B) - \eta^-(B)/c}{2} \tag{C.3}$$

We now make use of the first equation in (3.10), which implies that on line AC,

$$\gamma^\pm(B) + \frac{\eta^\pm(B)}{c} - \gamma^\pm(A) - \frac{\eta^\pm(A)}{c} = -\frac{1}{h}\int_{l_A}^{x_B} T(x)dx \tag{C.4}$$

Employing (2.10) to represent the integral in (C.4), we obtain the expression

$$\gamma^\pm(B) + \frac{\eta^\pm(B)}{c} - \gamma^\pm(A) - \frac{\eta^\pm(A)}{c} = \gamma_{\mathrm{s}}(x_B) - \gamma_{\mathrm{s}}(l_A) \tag{C.5}$$

According to (3.19),

$$\delta^-(A) = -\gamma_s(l_A) + \gamma^-(A) - \frac{\eta^-(A)}{c} \tag{C.6}$$

Hence, utilizing (C.5) and (C.6) in (C.3) yields

$$\Delta\gamma(B) = \frac{\Delta\eta(B)}{c} = \frac{\eta_t(l_A + 0) - \eta^-(A)}{c} \qquad (B \in AC) \tag{C.7}$$

where $\eta_t(l_A + 0)$ can be expressed from (B.10) (or (3.20)), with $P \to A$ and $B \to A$, as

$$\frac{\eta_t(l_A + 0)}{c} = \frac{\gamma_c^2 - [\gamma_s(l_A) + \delta^-(A)]^2}{2[\gamma_s(l_A) + \delta^-(A)]} \tag{C.8}$$

Therefore, the values of the discontinuity on line AC (figures 3c and B.1) are independent of B. These values on line CA_1 can be obtained similarly using (C.1) for point Q in region ACA_1 and (B.14) (or (3.23)) for point K in region CC_1A_1 (figures 3c and B.1). We consider the limit where points Q and K approach the same point M on line CA_1, that is, point M is approached from the opposite sides of CA_1 (figures 3c and B.1).

The limits of $Q \to M$ and $K \to M$ correspond to the sets of limits of $\{ B \to C, P \to A_1, R \to P, D \to B \}$ and $\{ R \to A, D \to A \}$, respectively (figures 3c and B.1). Hence, (C.1) and (B.14) (or (3.23)) yield

$$\begin{cases} \gamma^+(M) = \dfrac{\eta_t(l_P)}{c} + \dfrac{\delta^-(B) + \delta^-(C)}{2} + \gamma_s(x_M) \\ \dfrac{\eta^+(M)}{c} = \dfrac{\eta_t(l_P)}{c} + \dfrac{\delta^-(B) - \delta^-(C)}{2} \end{cases} \tag{C.9}$$

and

$$\begin{cases} \gamma^-(M) = \dfrac{\eta_t(l_P) - \eta_t(l_A + 0)}{c} + \dfrac{\delta^-(B) - \delta^-(A)}{2} + \gamma_s(x_M) \\ \dfrac{\eta^-(M)}{c} = \dfrac{\eta_t(l_P) + \eta_t(l_A + 0)}{c} + \dfrac{\delta^-(B) + \delta^-(A)}{2} \end{cases} \tag{C.10}$$

respectively. We, therefore, obtain the values

$$\Delta\gamma(M) = -\frac{\Delta\eta(M)}{c} = \frac{\eta_t(l_A + 0)}{c} + \frac{\delta^-(C) + \delta^-(A)}{2} \qquad (M \in CA_1) \tag{C.11}$$

of the discontinuity on CA_1. These values, $\Delta\gamma(M) = \gamma^+(M) - \gamma^-(M)$ and $\Delta\eta(M) = \eta^+(M) - \eta^-(M)$, are independent of M.

To compare (C.7) and (C.11), we note that according to (3.19),

$$\delta^-(C) = -\gamma_s(x_C) + \gamma^-(C) - \frac{\eta^-(C)}{c} = -\frac{\eta^-(C)}{c} \tag{C.12}$$

where we took into account that $\gamma_s(x_C) = \gamma_s(0) = \gamma_a$ and $\gamma^-(C) = \gamma_a$ (due to the first boundary condition in (2.14)). On the other hand, according to (C.5) (with $B \to C$)

$$\frac{\eta^-(C)}{c} = -\gamma_s(l_A) + \gamma^-(A) + \frac{\eta^-(A)}{c} \tag{C.13}$$

Hence,

$$\delta^-(C) = \gamma_s(l_A) - \gamma^-(A) - \frac{\eta^-(A)}{c} \tag{C.14}$$

and using (C.11) with (C.6) and (C.14) results in

$$\Delta\gamma(M) = -\frac{\Delta\eta(M)}{c} = \frac{\eta_t(l_A+0) - \eta^-(A)}{c} \quad M \in CA_1) \tag{C.15}$$

which is the same as (C.7).

Combining equations (C.7) and (C.15) yields

$$\Delta_0 = \Delta\gamma(B) = \frac{\Delta\eta(B)}{c} = \Delta\gamma(M) = -\frac{\Delta\eta(M)}{c} = \frac{\eta_t(l_A+0) - \eta^-(A)}{c} \quad (B \in AC,\ \ M \in CA_1) \tag{C.16}$$

which is the same as equation (3.29) in the main text. This equation shows that the discontinuity magnitude does not change between reflections at the band tip, although the discontinuity in η changes sign as a result of reflection from $x = 0$. Both $\eta_t(l)$ and $|\gamma_t(l)|$ abruptly increase at each tip reflection and increase continuously (as l grows) between the reflections. That $\Delta\gamma = \Delta\eta/c$ and $\Delta\gamma = -\Delta\eta/c$ at points B and M, respectively, can be seen directly from (2.17). However, that $\Delta\gamma$ and $\Delta\eta$ are independent of points B or M is established by (C.16). Taking into account the sign change in normalization $\Gamma = -\gamma/\gamma_0$ in (3.30), equation (C.16) (or (3.29)) is fully consistent with the discontinuity plots in figures 6 (§4) and D.2 (Appendix D).

Our next goal is to represent the discontinuity magnitude at each subsequent step (region $A_1C_1C_2A_2$ in figures 3c and B.1) in the recurrence process through quantities obtained at the previous step (region ACC_1A_1 in figures 3c and B.1). In this case, (C.7) and (C.15) (or (3.29)) become

$$\Delta_1 = \Delta\gamma(B_1) = \frac{\Delta\eta(B_1)}{c} = \Delta\gamma(M_1) = -\frac{\Delta\eta(M_1)}{c} = \frac{\eta_t(l_{A_1}+0) - \eta^-(A_1)}{c} \tag{C.17}$$

Employing (C.10) with $M \to A_1$, $P \to A_1$, and $B \to C$ we have

$$\begin{cases} \gamma^-(A_1) = \dfrac{\eta_t(l_{A_1}-0) - \eta_t(l_A+0)}{c} + \dfrac{\delta^-(C) - \delta^-(A)}{2} + \gamma_s(x_{A_1}) \\ \dfrac{\eta^-(A_1)}{c} = \dfrac{\eta_t(l_{A_1}-0) + \eta_t(l_A+0)}{c} + \dfrac{\delta^-(C) + \delta^-(A)}{2} \end{cases} \tag{C.18}$$

Using (C.18) in (C.17) yields

$$\Delta_1 = \frac{\Delta\eta_t(l_{A_1})}{c} - \frac{\eta_t(l_A+0)}{c} - \frac{\delta^-(C) + \delta^-(A)}{2} \tag{C.19}$$

where

$$\frac{\Delta\eta_t(l_{A_1})}{c} = \frac{\eta_t(l_{A_1}+0) - \eta_t(l_{A_1}-0)}{c} \tag{C.20}$$

is the jump of the slip rate at the band tip when the tip is overtaken by the discontinuity. Utilizing (C.6), (C.14), and (C.16) in (C.19), we obtain

$$\Delta_1 = \frac{\Delta\eta_t(l_{A_1})}{c} - \Delta_0 \tag{C.21}$$

According to (B.10) (or (3.20)), written for P (with $P \to A_1$, $B \to C$) and P_1 (with $P_1 \to A_1$, $B_1 \to A_1$), in the vicinity of A_1 (figures 3c and B.1),

$$\begin{cases} \dfrac{\eta_t(l_{A_1}-0)}{c} = \dfrac{\gamma_c^2 - [\gamma_s(l_{A_1}) + \delta^-(C)]^2}{2[\gamma_s(l_{A_1}) + \delta^-(C)]}, \quad \dfrac{\eta_t(l_{A_1}+0)}{c} = \dfrac{\gamma_c^2 - [\gamma_s(l_{A_1}) + \delta^-(A_1)]^2}{2[\gamma_s(l_{A_1}) + \delta^-(A_1)]} \\ \gamma_t(l_{A_1}-0) = \dfrac{\gamma_c^2 + [\gamma_s(l_{A_1}) + \delta^-(C)]^2}{2[\gamma_s(l_{A_1}) + \delta^-(C)]}, \quad \gamma_t(l_{A_1}+0) = \dfrac{\gamma_c^2 + [\gamma_s(l_{A_1}) + \delta^-(A_1)]^2}{2[\gamma_s(l_{A_1}) + \delta^-(A_1)]} \end{cases} \tag{C.22}$$

where quantity $\delta^-(A_1)$ is found from (B.18) and (B.19) (or from (3.25)), with $B_1 \to A_1$, $P \to A$, and $B \to A$, by

utilizing again (C.6), (C.14), and (C.16). As a result, we have

$$\delta^-(A_1) = -\delta^-(A) - \frac{2\eta_t(l_A+0)}{c} = \delta^-(C) - 2\Delta_0 \tag{C.23}$$

Using (C.20) with the first two equations in (C.22) yields

$$\frac{\Delta\eta_t(l_{A_1})}{c} = \frac{\gamma_c^2\Delta_0}{[\gamma_s(l_{A_1}) + \delta^-(C) - 2\Delta_0][\gamma_s(l_{A_1}) + \delta^-(C)]} + \Delta_0 \tag{C.24}$$

Together with (C.21) and (2.11), this equation results in

$$\Delta_1 = \frac{\gamma_0^2\Delta_0}{n[2\Delta_0 - \gamma_s(l_{A_1}) - \delta^-(C)][-\gamma_s(l_{A_1}) - \delta^-(C)]} \tag{C.25}$$

Therefore, expressions (C.16) and (C.25) define the discontinuity values on lines A_1C_1 and C_1A_2 through quantities defined at point A (figures 3c and B.1) at the general step of the recurrence sequence. At the first step ($l_A = l_0$, $t_A = 0$), $\eta_t(l_A+0)/c = \gamma_0(n-1)/(2n)$ is given by (3.9) while $\eta^-(A) = 0$ due to the second initial condition in (3.2). We, therefore, obtain from (C.16) the value

$$\Delta_0 = \gamma_0\frac{n-1}{2n} \tag{C.26}$$

of the discontinuity emitted from the band tip when it begins growing dynamically at $t = 0$. Using $\delta^-(C) = 0$ (per (3.27) with $B \to C$) in (C.25) gives the value

$$\Delta_1 = \frac{\gamma_0^2\Delta_0}{n[\gamma_s(l_{A_1}) - 2\Delta_0]\gamma_s(l_{A_1})} \tag{C.27}$$

of the discontinuity after the first reflection from the tip. Here l_{A_1} is defined by (B.33) (or (3.16)).

In general, equation (C.25) defines the discontinuity value, Δ_1, at each step through its value, Δ_0, at the previous step. Because at the first step, $\Delta_0 > 0$ (per (C.26)) and because $\delta^-(C) \le 0$ and $\gamma_s(l_{A_1}) < 0$ at each step, (C.25) implies that $\Delta_1 > 0$. We further observe from (C.25) that

$$\Delta_1 < \frac{\gamma_0^2\Delta_0}{n\gamma_s^2(l_{A_1})} \tag{C.28}$$

Finally, because $\gamma_s^2(l_{A_1}) > \gamma_0^2$ and $n > 1$, we see that

$$\Delta_1 < \Delta_0 \tag{C.29}$$

In other words, as stated in §3b, the magnitude of the discontinuity reduces with each reflection from the band tip.

Because n can be considered being relatively close to 1 (§8c), the discontinuity magnitude is only a small fraction of γ_0 even prior to the first reflection from the tip (equation (C.26)). As the discontinuity propagates, it retains constant magnitude (equations (C.16) or (C.17)) if it does not reach the band tip. However, each reflection from the tip results in a reduction of the magnitude (equation (C.29)). This justifies the discontinuities in our model. This can also be seen in figure C.1, where the relative discontinuity change, Δ_1/Δ_0, after the first reflection from the band tip (equations (C.26) and (C.27)) is shown as a function of λ_*. The discontinuity change is more significant for smaller λ_* and for greater n, but in all cases $\Delta_1 < \Delta_0$, which is consistent with (C.29).

(b) Arrival of the discontinuity at the band tip

As discussed in §3b, the truncation of the recurrence process is characterized by the difference $\Delta t(x)$ (figures 3a and B.1a) between the arrival times of the discontinuity and the band tip to a given location x. Before the disconti-

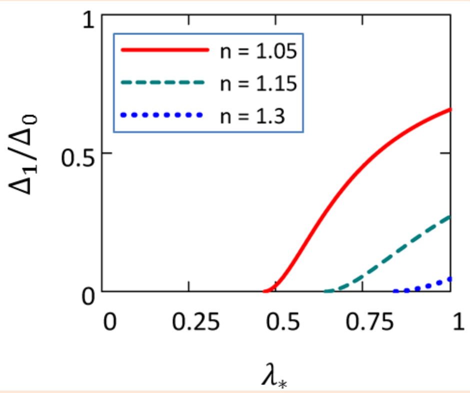

Figure C.1. Relative discontinuity magnitude Δ_1/Δ_0 as a function of λ_* for $n = 1.05$. (red, solid line), $n = 1.5$ (green, dashed line), and $n = 1.3$ (blue, dotted line). Although curves seemingly end at some λ_*, they were computed for the entire range of $0 \le \lambda_* \le 1$, but cannot be seen at the figure scale for smaller λ_*.

nuity reaches the band tip for the first time (first recurrence step), this difference is given by (equation (3.6))

$$\Delta t(x) = \frac{2}{c}\left[l_0 - \gamma_c^2 \int_{l_0}^{x} \frac{dl}{\gamma_s^2(l) - \gamma_c^2}\right] \tag{C.30}$$

Using (B.9), a similar representation is possible at the general recurrence step. Indeed, rewriting (B.9) as

$$\frac{dt_P}{dl_P} = \frac{1}{c}\frac{[\gamma_s(l_P) + \delta^-(B(l_P))]^2 + \gamma_c^2}{[\gamma_s(l_P) + \delta^-(B(l_P))]^2 - \gamma_c^2} \quad (l_A \le l_P < l_{A_1}) \tag{C.31}$$

and integrating (C.31) with condition $t_P(l_A) = t_A$, we represent $t_1(l)$ in (B.6) as

$$t_1(x) = t_A + \frac{x - l_A}{c} + \frac{2\gamma_c^2}{c}\int_{l_A}^{x} \frac{dl}{\left[\gamma_s(l) + \delta^-\left(B(l)\right)\right]^2 - \gamma_c^2} \tag{C.32}$$

Then

$$\Delta t(x) = t_A + \frac{l_A + x}{c} - t_1(x) = \frac{2}{c}\left[1 - \gamma_c^2 \int_{l_A}^{x} \frac{dl}{\left[\gamma_s(l) + \delta^-\left(B(l)\right)\right]^2 - \gamma_c^2}\right] \tag{C.33}$$

which reduces to (C.30) at the first recurrence step (when $\delta^-(B(l)) = 0$ per (3.27)).

Because $\delta^-(B(l)) \le 0$ and $\gamma_s(l) \le -\gamma_0 < -\gamma_c < 0$, the denominator in the integrand in (C.33) is positive. In turn, this suggests that $\Delta t(x)$ in (C.33) monotonically decreases with $x > l_A$. The discontinuity line CA_1 intersects the band tip line AA_1 (figure 3b) if $\Delta t(x)$ becomes negative. If $\Delta t(x)$ never becomes negative, these lines would not intersect and the discontinuity will always lag behind the band tip. Therefore, the necessary and sufficient condition for the recurrence sequence to truncate is

$$\delta t = \lim_{x\to\infty} \Delta t(x) \ge 0 \tag{C.34}$$

This consideration is particularly transparent at the first recurrence step. When the discontinuity propagating along CA_1 reaches the shear band tip (figures 3b and B.1a), it reflects and propagates back towards $x = 0$. Whether this takes place already at the first step (when $l_A = l_0$) can be determined from (C.30). Because $\gamma_s^2(l) - \gamma_c^2 > 0$, $\Delta t(x)$ in (C.30) monotonically decreases with x. If condition $\delta t = \lim_{x\to\infty} \Delta t(x) < 0$ is not satisfied (i.e., $\delta t \ge 0$), the discontinuity will never reach the band tip (figure 3a). In this case, while the tip remains slower than the discontinuity ($v < c$), its velocity, v, approaches the discontinuity velocity, c, and it has the head start, $x = l_0$, which is large enough for the band tip to always remain ahead the discontinuity (figure 3a). For this to occur, the integral in (C.30) must converge as $x \to \infty$, which essentially depends on the distribution of $T(x,t)$. For example, it is sufficient if $|\gamma_s(l)|$ increases with $l \to \infty$ faster than $\sqrt{l}$. This is probably typical for many practical cases, but not sufficient for $\delta t < 0$ to be satisfied. Then $\delta t \ge 0$, and the discontinuity always lags behind the band tip.

In the case of the homogeneous distribution (4.1), condition (C.34) simplifies to

rspa.royalsocietypublishing.org *Proc. R. Soc. A* 472: 20150758

$$\frac{c\delta t}{l_0} = 2 - \frac{\lambda_*}{\sqrt{n}} \ln\left(1 + \frac{2}{\sqrt{n} - 1}\right) \geq 0 \tag{C.35}$$

which was used for the plot in figure 4a.

Appendix D. Strain and slip rate distributions

(a) Downhill propagation

This appendix presents additional results for the case of homogeneous loading and downhill propagation of the shear band (figure 2) discussed in §4. As the shear band grows, the strain and slip rate near the band tip increase as can be seen in figures 5b and D.1a for $n = 1.1$ and $n = 1.2$, respectively ($\lambda_* = 0.75$ in both cases). After the discontinuity reaches the band tip (when $\lambda = \xi_{A_1} = 1.881$ for $n = 1.1$ and $\lambda = \xi_{A_1} = 8.430$ for $n = 1.2$), both tip strain, $\Gamma_t(\lambda)$, and tip slip rate, $\Omega_t(\lambda)$, in these figures monotonically grow and approach the same asymptote. figure 5b also shows $\Gamma_t(\lambda)$ and $\Omega_t(\lambda)$ for $n = 1.1$ and $\lambda_* = 0.25$. In this case, the discontinuity is always behind the band tip, but $\Gamma_t(\lambda)$ and $\Omega_t(\lambda)$ also grow with $\lambda = l/l_0$ and they also approach the same asymptote.

Figures D.1b and D.2 show similar patterns for $\lambda_* = 0.75$, $n = 1.2$ as those presented in figures 5a and 6 for $\lambda_* = 0.75$, $n = 1.1$. For example, the band tip location trends in figures 5a and D.1b are similar and so are the distributions of Γ and Ω along the slope displayed in figures D.2 and 6 for $\tau = 0.5$ (i.e., before the discontinuity reflects from $x = 0$ at $\tau = 1$) and for $\tau = 1.7$ (i.e., after it reflects from $x = 0$).

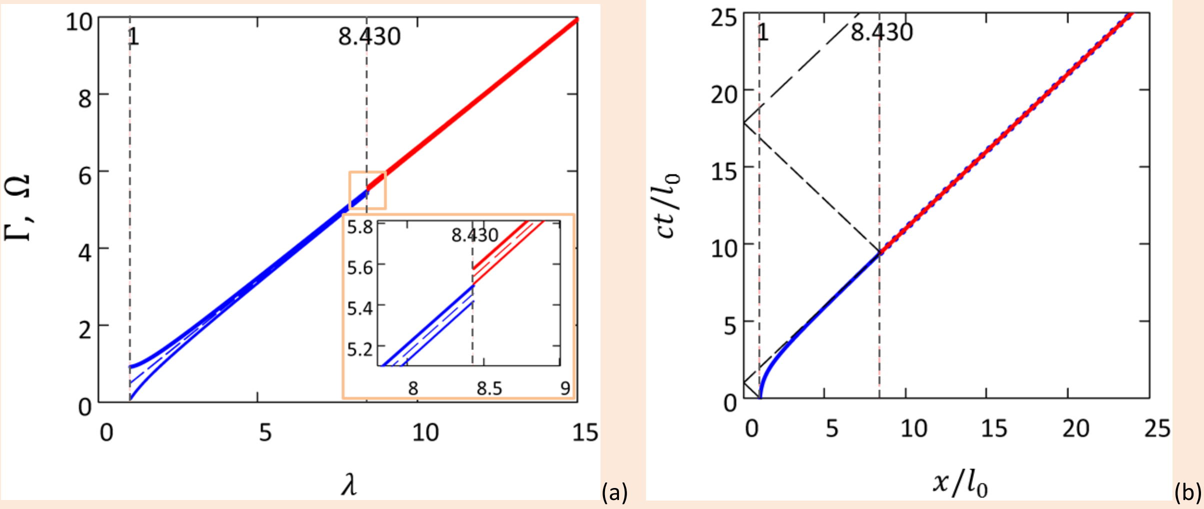

Figure D.1. (a) Normalized strain, $\Gamma_t = -\gamma_t/\gamma_0$ (solid, bold lines), and normalized slip rate, $\Omega_t = \eta_t/(\gamma_0 c)$ (solid, thin lines), at the shear band tip as functions of the normalized band length, $\lambda = l/l_0$, for $\lambda_* = 0.75$ and $n = 1.2$ before (blue lines) and after (red lines) the discontinuity arrives at the band tip, $\xi_{A_1} = 8.430$, at $\tau_{A_1} = 9.430$. Asymptotes of Γ_t and Ω_t at $\lambda \to \infty$ are shown by the dashed lines. (b) Normalized band size, $\lambda = l/l_0$, versus dimensionless time, $\tau = ct/l_0$, for $\lambda_* = 0.75$ and $n = 1.2$ before (blue, solid line) and after (red, solid line) the discontinuity (black, dashed lines) arrives at the band tip, $\xi_{A_1} = 8.430$, at $\tau_{A_1} = 9.430$. The solid blue line continues as a blue, dotted line. In this case, the effect of the discontinuity is negligible.

There are differences, however, with respect to the location of point A_1, where the discontinuity reflects from the band tip. For $\lambda_* = 0.75$ and $n = 1.1$, the reflection takes place at $\xi_{A_1} = \lambda_{A_1} = 1.881$, while for $\lambda_* = 0.75$ and $n = 1.24$, the discontinuity does not reach the band tip at all (figure 4a). Hence, for $\lambda_* = 0.75$, when n changes from 1.1 to 1.24, λ_{A_1} changes from 1.881 to ∞. In figure D.1b, plotted for $n = 1.2$, the point where the band tip is overtaken by the discontinuity is extended to $\xi_{A_1} = \lambda_{A_1} = 8.430$. As a result, strain distributions in figures D.1a and D.2 can be observed at a larger scale compared to figures 5a and 6, respectively.

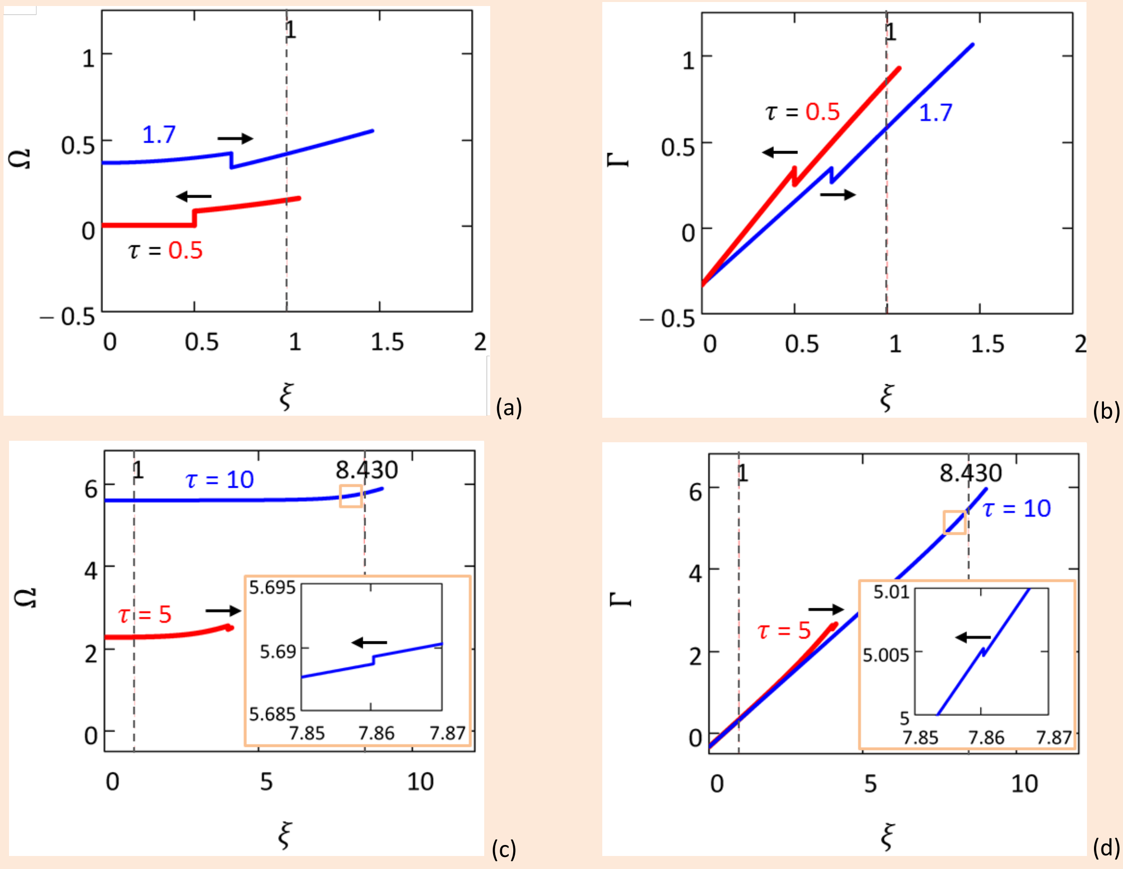

Figure D.2. Distributions of normalized (a, c) tip slip rate, $\Omega_\mathrm{t} = \eta_\mathrm{t}/(\gamma_0 c)$, and (b, d) tip strain, $\Gamma_\mathrm{t} = -\gamma_\mathrm{t}/\gamma_0$, along the slope as functions of dimensionless coordinate, $\xi = x/l_0$, plotted for dimensionless times, $\tau = ct/l_0$, of (a, b) 0.5 (when the discontinuity has not reflected yet from $x = 0$) and 1.7 (soon after it reflected from $x = 0$) and (c, d) 5 and 10 (before and after it reflected at the band tip, respectively) for $\lambda_* = 0.75$ and $n = 1.2$. The shear band propagates downhill. The magnitude $\Delta_1 = 5.741\times10^{-4}$ of the discontinuity after it reflects from the tip (blue lines in (c) and (d)) is two orders smaller than magnitude $\Delta_0 = 8.333\times10^{-2}$ before the reflection (red lines in (c) and (d)). The latter magnitude is the same as in (a) and (b). At $\tau = 0.5$ and $\tau = 10$ the discontinuity moves (as indicated by arrows) towards the end, $x = 0$, while at $\tau = 1.7$ and 5, it moves towards the band tip, $x = l(t)$.

For $n = 1.2$, the discontinuity is still relatively far from the tip at the dimensionless times $\tau = 0.5$ and 1.7 (figures D.2a, D.2b) since $\tau_{A_1} = 9.430$. For the dimensionless time $\tau = 5$ in figures D.2c, D.2d, the discontinuity is less visible because the magnitudes of strain and slip rate became considerably larger than before (i.e., for $\tau = 0.5$ and 1.7). The physical location of the discontinuity is closer to the propagating band tip for $\tau = 5$ than for $\tau = 0.5$ and 1.7 (figure 6). Figures D.2c and D.2d also display the distributions of Γ and Ω along the slope for an even larger time of $\tau = 10$ and the same parameters $\lambda_* = 0.75$ and $n = 1.2$.

Time $\tau = 10$ is after the discontinuity reflects from the band tip at point A_1, but before it reflects from $x = 0$ again at point C_1 (figure D.2d). Because of the reflection from the tip, the magnitude of the discontinuity decreases by two orders (from $\Delta_0 = 8.333\times10^{-2}$ before the reflection to $\Delta_1 = 5.741\times10^{-4}$ after), while the magnitudes of both strain and slip rate further increase with time. This again is a common trend (figure C.1), which justifies allowing discontinuities in the developed model.

Although in the beginning of the band propagation, the value of n may significantly affect the band length as well as the magnitudes of strain and the slip rate (e.g., equations (3.9)), the effect of the value of n on these parameters at slope failure is not significant. For a band propagating downhill, this can be seen from Tables D.1 and D.2 computed for $\gamma_0/\gamma_\mathrm{p} = 0.1$ and $\gamma_0/\gamma_\mathrm{p} = 0.5$, respectively.

Table D.1. Effect of n on the dynamic shear band, propagating downhill, for $\gamma_0/\gamma_\mathrm{p} = 0.1$ and different values of λ_*.

n	1.001	1.01	1.05	1.1	1.5	2	10	∞
				$\lambda_* = 0.1$				
$\bar{\Omega} = \bar{\eta}/(\gamma_0 c)$	6.857	6.857	6.858	6.859	6.864	6.868	6.877	6.879
$l_\mathrm{f}/L_\mathrm{f}$	1.524	1.524	1.524	1.524	1.525	1.525	1.526	1.526
v/c at $l = 3l_0$	0.995	0.996	0.996	0.996	0.997	0.998	0.999	1
				$\lambda_* = 0.25$				
$\bar{\Omega} = \bar{\eta}/(\gamma_0 c)$	8.649	8.649	8.650	8.651	8.657	8.662	8.671	8.674
$l_\mathrm{f}/L_\mathrm{f}$	1.765	1.765	1.766	1.766	1.767	1.767	1.769	1.769
v/c at $l = 3l_0$	0.976	0.976	0.977	0.978	0.984	0.988	0.998	1
				$\lambda_* = 0.5$				
$\bar{\Omega} = \bar{\eta}/(\gamma_0 c)$	9.446*	9.444*	9.447*	9.477	9.483	9.487	9.497	9.500
$l_\mathrm{f}/L_\mathrm{f}$	1.899*	1.899*	1.899*	1.905	1.906	1.907	1.909	1.909
v/c at $l = 3l_0$	0.924*	0.925*	0.927	0.930	0.948	0.961	0.992	1
				$\lambda_* = 1$				
$\bar{\Omega} = \bar{\eta}/(\gamma_0 c)$	9.805***	9.802**	9.808*	9.801*	9.958	9.962	9.972	9.975
$l_\mathrm{f}/L_\mathrm{f}$	1.969***	1.968**	1.969*	1.968*	1.997	1.997	1.999	2.000
v/c at $l = 3l_0$	0.819***	0.821**	0.827*	0.836*	0.862	0.895	0.978	1

No asterisk corresponds to the times before the first arrival of the discontinuity to the band tip.
*Computed between the first and second arrivals of the discontinuity to the band tip.
**Computed between the second and third arrivals of the discontinuity to the band tip.
***Computed after the third arrival of the discontinuity to the band tip (figure B.1).

Table D.2. Effect of n on the dynamic shear band propagating downhill for $\gamma_0/\gamma_\mathrm{p} = 0.5$ and different values of λ_*.

n	1.001	1.01	1.05	1.1	1.5	2	10	∞
				$\lambda_* = 0.1$				
$\bar{\Omega} = \bar{\eta}/(\gamma_0 c)$	0.508	0.508	0.511	0.514	0.532	0.543	0.570	0.577
$l_\mathrm{f}/L_\mathrm{f}$	1.157	1.158	1.159	1.160	1.166	1.170	1.180	1.182
v/c at $l = 3l_0$	0.995	0.996	0.996	0.996	0.997	0.998	0.999	1
				$\lambda_* = 0.25$				
$\bar{\Omega} = \bar{\eta}/(\gamma_0 c)$	0.960	0.961	0.966	0.971	0.999	1.018	1.061	1.071
$l_\mathrm{f}/L_\mathrm{f}$	1.346	1.347	1.349	1.352	1.365	1.374	1.395	1.400
v/c at $l = 3l_0$	0.976	0.976	0.977	0.978	0.984	0.988	0.998	1
				$\lambda_* = 0.5$				
$\bar{\Omega} = \bar{\eta}/(\gamma_0 c)$	1.341*	1.343*	1.373	1.379	1.413	1.435	1.487	1.500
$l_\mathrm{f}/L_\mathrm{f}$	1.566*	1.566*	1.582	1.586	1.609	1.624	1.658	1.667
v/c at $l = 3l_0$	0.924*	0.925*	0.927	0.930	0.947	0.961	0.992	1
				$\lambda_* = 1$				
$\bar{\Omega} = \bar{\eta}/(\gamma_0 c)$	1.591***	1.605**	1.597*	1.603*	1.782	1.806	1.862	1.875
$l_\mathrm{f}/L_\mathrm{f}$	1.778***	1.777**	1.780*	1.778*	1.913	1.935	1.987	2.000
v/c at $l = 3l_0$	0.819***	0.821**	0.827*	0.836*	0.862	0.895	0.978	1

No asterisk corresponds to the times before the first arrival of the discontinuity to the band tip.
*Computed between the first and second arrivals of the discontinuity to the band tip.
**Computed between the second and third arrivals of the discontinuity to the band tip.
***Computed after the third arrival of the discontinuity to the band tip (figure B.1).

rspa.royalsocietypublishing.org Proc. R. Soc. A 472: 20150758

(b) Uphill propagation (§8a)

For a shear band propagating downhill (figure 2) and uphill (figure 8), patterns of the band tip location (figures D.1b and D.3a, respectively) and growth velocity (figures 4b and D.3b, respectively) are similar and so are the trends of the tip strain and tip slip rate (figure 5b, or D.1a, and figure D.4, respectively). For $\lambda_* = 2$ and $n = 1.1$, the discontinuity in the band propagating uphill experiences two reflections from the tip, when $\lambda = 1.185$ ($\tau = 2.185$) and $\lambda = 3.733$ ($\tau = 7.103$). When the band tip is overtaken by the discontinuity for the first time, tip strain, Γ_t, slip rate at the tip, Ω_t, and the tip velocity, V, experience jumps, which may not necessarily be small. For example, $\Delta V = V(\xi_{A_1} + 0) - V(\xi_{A_1} - 0) = 7.746\times10^{-2}$, so that $\Delta V/V(\xi_{A_1} - 0) = 57.2\%$. These jumps, however, become practically negligible (in the relative sense) already at the second reflection of the discontinuity from the tip. For example, $\Delta V = V(\xi_{A_2} + 0) - V(\xi_{A_2} - 0) = 1.365\times10^{-2}$ while $\Delta V/V(\xi_{A_1} - 0) = 1.78\%$.

Similar to the downhill propagation (Tables D.1 and D.2), parameters of the shear band at failure are not very sensitive to the value of n (Table D.3). As in the downhill growth, the sliding layer above the shear band is loaded (strained) less in the dynamic than in static condition (figure D.4). Hence, the failure length is again underestimated by the static analysis (Table D.3).

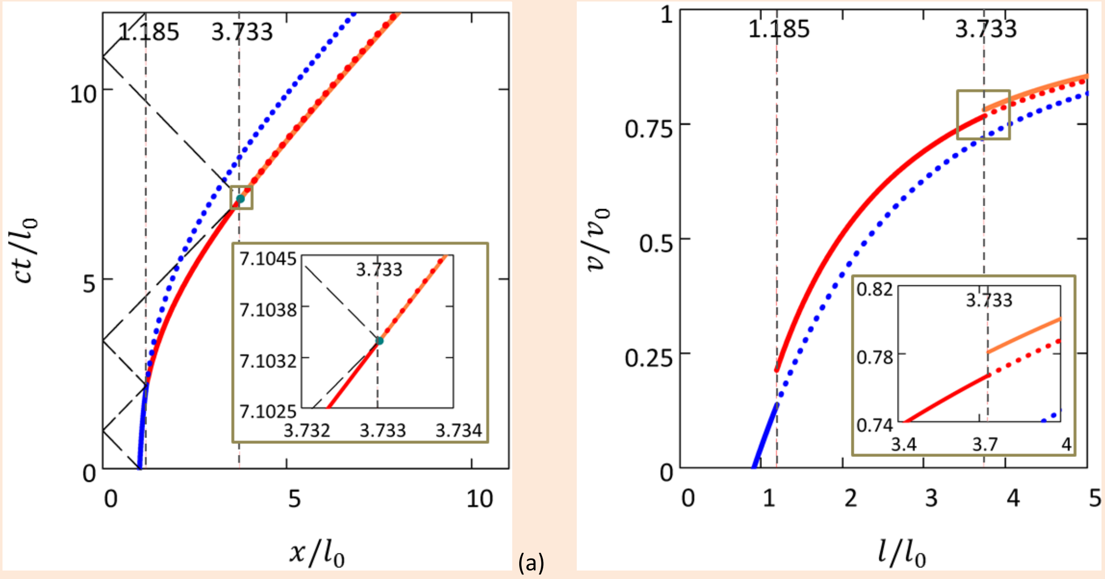

Figure D.3. Uphill propagation of the shear band. (a) Time, $\tau = ct/l_0$, of arrival of the band tip at $\xi = x/l_0$ and (b) dimensionless velocity, $V = v/c$, of the shear band as a function of its dimensionless length, $\lambda = l/l_0$, plotted (with λ_* and l_0 redefined in §8a) for $\lambda_* = 2$ and $n = 1.1$. The discontinuity first reaches the band tip at point $\xi_{A_1} = 1.185$ (when $\tau_{A_1} = 2.185$). The discontinuity reaches the band tip for the second time at point $\xi_{A_2} = 3.733$ (when $\tau_{A_2} = 7.103$). Dependencies are plotted before the discontinuity reflects from the tip for the first time (blue, solid and dotted lines), between the first and second tip reflections (red, solid and dotted lines), and after the second tip reflection (orange line). For the sake of comparison, dotted lines are plotted (with the same color as solid lines) after the corresponding tip reflection using equations valid before the reflection. Insets show magnified views near the second reflection of the discontinuity from the band tip.

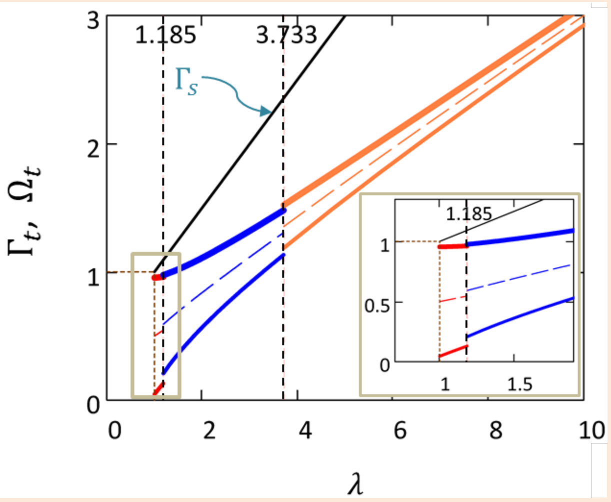

Figure D.4. Strain, Γ_t, and slip rate, Ω_t, at the shear band tip (solid lines, bold and of intermediate thickness, respectively) versus band length, $\lambda = l/l_0$, for $n = 1.1$, uphill propagation (figure 8), and $\lambda_* = 2$ (with λ_* defined by (8.2)). Γ_t and Ω_t are plotted before the discontinuity arrives at the tip (solid red lines), between the first and second arrivals at the tip (blue, solid lines), and after the second tip arrival (orange, solid lines). Asymptotes of Γ_t and Ω_t at $\lambda \to \infty$ are shown by the dashed lines. Thin, black, solid line shows static strain $\Gamma_s = -\gamma_s/\gamma_0$ with γ_s defined by (8.1).

Table D.3. Effect of n on the dynamic shear band propagating uphill for $\gamma_0/\gamma_p = 0.1$ and different values of λ_*.

n	1.01	1.05	1.1	1.5	2	10	∞
			$\lambda_* = 1.1$				
$\bar{\Omega} = \bar{\eta}/(\gamma_0 c)$	9.828**	9.841*	9.831*	10.004	10.008	10.018	10.021
l_f/L_f	1.973**	1.976*	1.974*	2.006	2.007	2.009	2.009
v/c at $l = 3l_0$	0.804**	0.808*	0.819*	0.845	0.882	0.975	1
			$\lambda_* = 1.25$				
$\bar{\Omega} = \bar{\eta}/(\gamma_0 c)$	9.859**	9.879**	9.868*	9.861*	10.063	10.073	10.076
l_f/L_f	1.980**	1.982**	1.981*	1.977*	2.018	2.020	2.020
v/c at $l = 3l_0$	0.778**	0.782*	0.794*	0.820	0.862	0.971	1
			$\lambda_* = 1.5$				
$\bar{\Omega} = \bar{\eta}/(\gamma_0 c)$	9.898***	9.906**	9.931**	9.891*	10.132	10.142	10.144
l_f/L_f	1.988***	1.988**	1.991**	1.984*	2.032	2.034	2.034
v/c at $l = 3l_0$	0.739***	0.751**	0.757*	0.782	0.832	0.964	1
			$\lambda_* = 2$				
$\bar{\Omega} = \bar{\eta}/(\gamma_0 c)$	9.946***	9.938**	9.951**	9.935*	9.931*	10.228	10.231
l_f/L_f	1.998***	1.996**	1.997**	1.993*	1.991*	2.052	2.053
v/c at $l = 3l_0$	0.718***	0.693**	0.692*	0.787*	0.778	0.951	1

No asterisk corresponds to the times before the first arrival of the discontinuity to the band tip.
*Computed between the first and second arrivals of the discontinuity to the band tip.
**Computed between the second and third arrivals of the discontinuity to the band tip.
***Computed after the third arrival of the discontinuity to the band tip (figure B.1).
